# Morphological phylogeny on the unnatural grouping of *Demidospermus*-like species (Monopisthocotyla, Dactylogyridae) with the proposal of new genera, genera resurrections, and descriptions of new species

**DOI:** 10.1051/parasite/2025034

**Published:** 2025-08-05

**Authors:** Julio Cesar Cenci de Aguiar, Patrick D. Mathews, Marcus Vinicius Domingues, Reinaldo J. da Silva

**Affiliations:** 1 Department of Biodiversity and Biostatistics, Section of Parasitology, Institute of Biosciences, São Paulo State University (UNESP) 18618-689 Botucatu São Paulo Brazil; 2 Instituto de Estudos Costeiros, Federal University of Pará (UFPA) 68600–000 Bragança Pará Brazil

**Keywords:** Biodiversity, Fish, Homoplasy, Neodermata, Neotropical, Parasitism

## Abstract

Dactylogyrids are flatworms of ecological and economic significance, parasitizing fish worldwide. In recent years, there has been a surge in the description of Neotropical dactylogyrids, particularly those infecting siluriform fishes. While these studies have contributed to the organization of some genera and refined species boundaries through integrative taxonomy, certain groups within the family, such as *Demidospermus*, remain taxonomically unstable. This study focuses on *Demidospermus*, aiming to reclassify species of uncertain status into appropriate genera and establish a morphological framework to support future evolutionary analyses and taxonomic revisions within the *Demidospermus*-like species group. Supported by morphological phylogenetic analysis, we propose the new genera *Rhabdolachosus* n. gen., *Martorellius* n. gen., *Magnanchistrius* n. gen., and *Sicohencotyle* n. gen., along with the resurrection of *Omothecium* Kritsky, Thatcher & Boeger, 1987, and *Paramphocleithrium* Suriano & Incorvaia, 1995. Additionally, two new species are described: *Sicohencotyle antoniomaiai* n. gen. n. sp. and *Ameloblastella sakulocirra* n. sp. Also, *Demidospermus centromochi* Mendoza-Franco & Scholz, 2009 is classified as *sedis mutabilis*, while *D. annulus* Marcotegui & Martorelli, 2011, *D. brevicirrus* Mendoza-Palmero *et al.*, 2012, *D. cornicinus* Kritsky & Gutierrez, 1998, *D. idolus* Kritsky & Gutierrez, 1998, *D. armostus* Kritsky & Gutierrez, 1998, *D. mortenthaleri* Mendoza-Palmero *et al.*, 2012, *D. osteomystax* Tavernari *et al.*, 2010, *D. tocantinensis* Cohen *et al.*, 2020, *D. doncellae* Morey *et al.*, 2024, *D. bifurcatus* Justo, Martins & Cohen, 2024, *D. juruaensis* Justo, Martins & Cohen, 2024, and *D. takemotoi* Justo, Martins & Cohen, 2024 are considered *incertae sedis*. Lastly, *Urocleidoides amazonensis* Mizelle & Kritsky, 1969 remains classified as *incertae sedis*.

## Abstract (fr)

Les Dactylogyridae sont des vers plats d’importance écologique et économique, parasitant les poissons du monde entier. Ces dernières années, on a assisté à une forte augmentation de la description de Dactylogyridae néotropicaux, en particulier ceux qui infectent les poissons siluriformes. Si ces études ont contribué à l’organisation de certains genres et à l’affinement des frontières entre espèces grâce à une taxonomie intégrative, certains groupes de la famille, comme *Demidospermus*, demeurent taxonomiquement instables. Cette étude se concentre sur *Demidospermus* et vise à reclasser les espèces au statut incertain dans des genres appropriés et à établir un cadre morphologique pour étayer les futures analyses évolutives et révisions taxonomiques au sein du groupe d’espèces apparentées à *Demidospermus*. En nous appuyant sur une analyse phylogénétique morphologique, nous proposons les nouveaux genres *Rhabdolachosus* n. gen., *Martorellius* n. gen., *Magnanchistrius* n. gen. et *Sicohencotyle* n. gen., ainsi que la résurrection d’*Omothecium* Kritsky, Thatcher & Boeger, 1987 et de *Paramphocleithrium* Suriano & Incorvaia, 1995. De plus, deux nouvelles espèces sont décrites : *Sicohencotyle antoniomaiai* n. gen. n. sp. et *Ameloblastella sakulocirra* n. sp. *Demidospermus centromochi* Mendoza-Franco & Scholz, 2009 est classé comme *sedis mutabilis*, tandis que *D. annulus* Marcotegui & Martorelli, 2011, *D. brevicirrus* Mendoza-Palmero *et al.*, 2012, *D. cornicinus* Kritsky & Gutierrez, 1998, *D. idolus* Kritsky & Gutierrez, 1998, *D. armostus* Kritsky & Gutierrez, 1998, *D. mortenthaleri* Mendoza-Palmero *et al.*, 2012, *D. osteomystax* Tavernari *et al.*, 2010, *D. tocantinensis* Cohen *et al.*, 2020, *D. doncellae* Morey *et al.*, 2024, *D. bifurcatus* Justo, Martins & Cohen, 2024, *D. juruaensis* Justo, Martins & Cohen, 2024 et *D. takemotoi* Justo, Martins & Cohen, 2024, sont considérés comme *incertae sedis*. *Urocleidoides amazonensis* Mizelle & Kritsky, 1969 reste classé comme *incertae sedis*.

## Introduction

**Figure S1:**
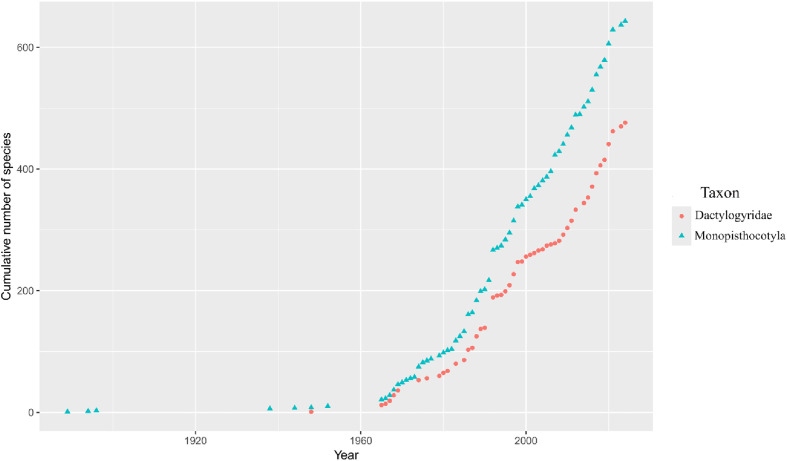
Accumulation curve of described species of Monopisthocotyla and Dactylogyridae in South America.

In the Neotropical Region, although the first reports of monopisthocotyls date back to the 19th century, it was only from the 1960s onward that records and descriptions of unknown species became more frequent [10, 20]. Since then, solely on the South American continent, around 650 species have been reported (Figure S1). Much of this diversity belongs to a single lineage, the Dactylogyridae, which accounts for 476 species (73%) described so far (for references, see S1). However, this knowledge is incipient since less than 10% of South American fish species have been examined for parasitological studies [56]. These data suggest that on the continent, less than 5% of dactylogyrid species have been recognized, considering the specificity, subsampling, and taxonomic entanglements that can impact these values.

Dactylogyridae, recognized as a monophyletic group, is composed of nine major lineages (*i.e.*, Anacanthorinae, Ancylodiscoidinae, Ancyrocephalinae, Dactylogyrinae, Hareocephalinae, Heterotesiinae, Linguadactylinae, Linguadactyloidinae and Pseudodactylogyrinae), which were identified through phylogenetic inference based on morphological characters [47]. However, more recently, Pseudodactylogyrinae and part of Ancyrocephalinae have been synonymized with Dactylogyrinae, and certain Neotropical freshwater and marine ancyrocephaline species have been transferred to Dactylogyrinae based on both molecular and morphological data [46]. With approximately 1,950 species [6], these parasites are globally distributed and found in association with fish across various taxonomic groups [10, 46, 85]. They inhabit diverse environments, including freshwater, estuarine, and marine ecosystems, and occupy infection sites such as the gills, nasal cavities, skin, fins, ureters, and the urinary bladder [10, 46, 85]. While often overlooked, these parasites can occasionally cause severe inflammatory reactions, tissue lesions [13, 60, 100], and hematological alteration [43] in their hosts, potentializing host mortalities in the wild [41] and captivity [49], with consequent environmental damage and economic losses.

Recognizing the evolutionary history of parasite lineages is key to identifying which ones have maintained long-standing associations with their hosts and which may, under certain conditions (*e.g.*, habitat loss and destruction, overexploitation of natural resources, pollution, and climate changes), colonize new hosts, triggering outbreaks of infectious diseases [7, 11, 42]. However, accurately assessing these dynamics depends on the proper delimitation of species within these lineages. Within Dactylogyridae, certain taxonomic impasses can hinder understanding its natural history [46], as evidenced for *Demidospermus* Suriano, 1983.

Originally, *Demidospermus* was erected to accommodate *Demidospermus anus* Suriano, 1983, a gill parasite of *Loricariichthys anus* (Valenciennes, 1835) from Laguna de Chascomús, Buenos Aires, Argentina. This genus was characterized to have gonads in tandem, testes posterior to the germarium, a coiled or J-shaped male copulatory organ (MCO), a V- or U-shaped ventral and/or dorsal bar, and packets of sperm inside the testicle, inspiring the name of the genus [96]. Since its initial description, subsequent studies have aimed to refine the genus diagnosis and expand our understanding of this group. Gutiérrez & Suriano (1992) [39] described three additional species and amended the diagnosis, emphasizing body size and the presence of a left sclerotized vagina as key characteristics. However, they overlooked potentially informative features such as the presence of dilated hooks, recurved anchor tips, and a medial projection on the dorsal bar. Later, Kritsky and Gutiérrez (1998) [48], described five more species, synonymized *Paramphocleithrium* Suriano & Incorvaia, 1995 and *Omothecium* Kritsky, Thatcher & Boeger, 1987 with *Demidospermus*, and further amended the diagnosis. They incorporated variability in MCO shape (coiled or not coiled) and the presence of U-shaped bars as diagnostic traits and expanded the known host range to include Auchenipteridae. Despite these valuable contributions, certain potentially informative characters, such as the presence or absence of articulation between the accessory piece and the male copulatory organ, were not considered to be diagnostic features in *Demidospermus* species.

With the descriptions of additional *Demidospermus* species in Peru [64, 68, 69, 75], Brazil [2, 16, 19, 26, 45, 74, 99], and Argentina [59], and concomitant reclassifications and synonym recognition [2, 21, 69], the morphological boundaries characterizing the genus have expanded. All these studies resulted in the current gathering of 36 *Demidospermus* species, parasites of auchenipterids, doradids, heptapterids, loricariids, and pimelodids (*i.e.*, Siluriformes) distributed throughout the South American rivers [2, 19, 23, 26, 45, 75].

With unusual morphological and parasitological variation for a Dactylogyridae lineage, it became increasingly clear that *Demidospermus* had become a catch-all taxon. Mendoza-Palmero *et al.* (2015) [65], in their phylogenetic inference using partial 28S rDNA with *Demidospermus* species (among other Neotropical species) as terminals, suggested that the genus is not monophyletic. However, the authors did not have access to fresh material of the type species or closely related to the type species, making it impossible for a formal proposition to reorganize the clade. Nevertheless, other studies using the same molecular marker would throw light on this situation and help to circumscribe *Demidospermus*.

Franceschini *et al.* (2018) [26] sequenced *D. anus*, recovered parasitizing *Loricariichthys platymetopon* Isbrücker & Nijssen, 1979, from the Upper Paraná River in Brazil. This location and host differ from those of the type specimens, and the sequenced specimen exhibited some morphological variations compared to the original description of the type species. Furthermore, they described other morphologically similar species, all parasitic on loricariids, sharing similarities with *D. anus*, particularly in the morphology of the ventral and dorsal bars. Their phylogenetic analysis suggested that this *Demidospermus* subclade, comprising loricariid parasites, forms a monophyletic group. The *Demidospermus sensu stricto* was subsequently corroborated by Acosta *et al.* (2018) [2] using the same molecular marker when the authors described another species of this group. However, a resolution for the remaining, here termed *Demidospermus*-like species has yet to be achieved, even in the most recent molecular phylogenetic analyses.

Despite recent research describing numerous Neotropical dactylogyrids parasitic on catfishes and inferring their molecular phylogenies, the taxonomic problem surrounding *Demidospermus* requires a focused effort to organize the remaining species. To address this gap, we reviewed a representative collection of Neotropical dactylogyrids, supplemented by newly collected samples from the Amazon River basin, Brazil, focusing on a morphological phylogenetic approach. The results presented here, while provisional, are consistent and support a hypothesis of relationships for the majority of *Demidospermus*-like species. This work necessitated the erection of new genera, the resurrection of previously synonymized genera, the description of new species, and the designation of some species as *sedis mutabilis* and *incertae sedis*.

## Material and methods

### Ethical approval

This research was approved by the State University of Campinas-UNICAMP Ethics Committee (CEUA No. 3179-1), and specimen collection was authorized by the Brazilian Ministry of the Environment through the Biodiversity Authorization and Information System (SISBIO No. 42427-3).

### Taxa sampling and morphological investigations

Dactylogyrids examined were selected considering their similarity with *Demidospermus* species, particularly those with a sinistral vagina aperture and those previously classified within the genus, all parasitic on Neotropical catfishes. Morphological investigations were conducted through direct analysis and/or literature review. Specimens deposited in the “Coleção Helmintológica do Instituto Oswaldo Cruz” (CHIOC) and “Instituto Nacional de Pesquisa da Amazônica” (INPA), both from Brazil, and, at Invertebrate Zoology Collections of the National Museum of Natural History, Smithsonian (USNM), from the United States, were consulted (Table S1). Additional material, including that of two newly described species, was collected from the “redtail catfish”, *Phractocephalus hemioliopterus* (Bloch & Schneider, 1801) captured from the Tapajós River Basin (Table 1).

**Table 1:** Summary of field samplings, with geographic locality of capture (longitude and latitude) of *Phractocephalus hemioliopterus* and the number of fish caught (*n*).

Sample area	Long.; Lat.	*n*	Total length (cm)	Weight (g)	Catch dates
Tapajós River, Pimental, Itaituba, PA	−56.264613°; −4.568505°	2	85 (72.5–97)	8000	October 2011
Tapajós River, National Park of Amazonia, Itaituba, PA	−56.299889°; −4.552694°	3	60 (40–75)	3004 (713–5400)	June 2012
Igarapé Jari, Tapajós River Basin, Santarém, PA	−54.876133°; −2.334050°	4	50 (37.5–73)	962.5 (550–1500)	October 2014

When dealing with fresh material, the helminths were mounted with Gray and Wess’s medium [36] to study their sclerotized structures, while other specimens were stained with Gomori’s trichrome [34] and mounted in Dammar gum to examine soft internal structures. Specimens from the Laboratory of Wildlife Parasitology (LAPAS) collection, such as *Demidospermus anus* Suriano, 1983 from *Loricariichthys platymetopon* (Paraná River, Porto Rico locality, state of Paraná, Brazil), underwent proteolytic digestion following Aguiar *et al.* [5] to investigate haptoral structures. Measurements, taken from digitally processed images using ImageJ 1.43 [82] and expressed in micrometers, represent direct linear distances between the two farthest points of structures, according to Mizelle and Klucka [71], with exceptions for the male copulatory organ (MCO) and bars, which were fully measured. Some specimens were observed and photographed using differential interference contrast (DIC) and phase-contrast optics through an Axioplan 2 Zeiss microscope. Illustrations were made through a drawing tube attached to a Leica DM 2500 or an Olympus BX 51 microscope, both equipped with DIC.

Scanning electron microscopy (SEM) was performed on specimens of *Vancleaveus cicinnus* Kritsky, Thatcher & Boeger, 1986 to verify the position of its vaginal aperture. For this analysis, specimens were fixed in 4% formalin, then transferred to 70% ethanol, and subsequently hydrated in distilled water. After washes and cleaning, specimens were immersed in 1% osmium tetroxide for 2 h, dehydrated in an ethanol series, critical point dried, and gold-coated. They were then examined using a JEOL JSM 35 scanning electron microscope (10 kV) at the Electron Microscopy Laboratory, Institute of Biology, State University of Campinas.

Type specimens and vouchers were deposited in the collection of Platyhelminthes of the Adão José Cardoso Museum of Zoology of the State University of Campinas, São Paulo (ZUEC PLA), the Museum of Zoology of the University of São Paulo (MZUSP), the Helminthological Collection of the Oswaldo Cruz Institute (CHIOC), and in the Helminthological Collection at the Institute of Biosciences at the São Paulo State University (CHIBB). Quantitative descriptors of the parasitic population follow Bush *et al.* [12].

### Character analysis and dataset construction

A matrix of characters consisting of 46 taxa (44 from the ingroup and 2 used as outgroup) was built. Rooting was made in *Vancleaveus janauacaensis* Kritsky, Thatcher & Boeger, 1986, though *Vancleaveus cicinnus* Kritsky, Thatcher & Boeger, 1986 has also been used as an outgroup. These species are morphologically similar to the species of *Demidospermus*, beyond parasitizing catfishes. However, they share synapomorphies, such as the presence of a fold in the superficial root of the dorsal anchor and a ventral vagina, which distinguish them from other *Demidospermus*-like species that do not have such a fold and bear a sinistral vagina.

The ingroup comprises the following *taxa*: 4 members of *Demidospermus sensu stricto*, 24 of *Demidospermus lato sensu*, 2 *incertae sedis* species of *Urocleidoides*, 6 species of *Ameloblastella* Kritsky, Mendoza-Franco e Scholz, 2000, one of them described here, 3 species of *Aphanoblastella* Kritsky, Mendoza-Franco & Scholz, 2000, 4 of *Nanayella* Acosta, Mendoza-Palmero, Silva & Scholz, 2019, and 1 of a newly proposed genus. A total of 60 morphological characters were obtained from specimens deposited in reference collections, aiming to extract the greatest possible morphological variation, therefore most characters were directly observed. To complete the range of meristic characters, *i.e.*, intra and interspecific variation, published data were also used, provided they were recent and/or contained adequate descriptions for the interpretation of the characters.

Most of the characters were obtained from sclerotized structures, but soft tissue structures were also included. The 60 characters were extracted from haptoral structures, including bars, anchors, and hooks (27 characters), peduncle length, used as discrete character (1 character), MCO and accessory piece (11 characters), vagina and vaginal canal (14 characters), epidermal structures (3 characters), head organs (1 character), and internal soft tissue organs (3 characters). When morphological variations were observed, they were coded as polymorphic. The data matrix was constructed in WinClada ver. 1.00.08 [77] and it is provided herein as supplementary material concerning a list of examined taxa (Table S1), a spreadsheet (Table S2), and nexus (Table S3) matrices, as well as a commented list of characters (S1.1). This list offers valuable insights into character states, justifications for particular traits, the number of steps each character undergoes (with a higher number indicating greater homoplasy), and indices such as the consistency index (CI) and retention index (RI). These indices measure the degree of homoplasy for each character, where a value of 0 signifies maximum homoplasy, and a value of 1 indicates no homoplasy.

### Phylogenetic inferences

Phylogenetic inferences were made using TNT ver. 1.6 [29]. Tree searches were set out through new technologies that implement more algorithms compared to simple branch-swapping [28]. A max ram was set up to 1,000 (*mxram 1000;*), characters were read as alpha-numeric (*nstates num;*), and gaps were considered missing data (*nstates nogaps;*). Extended implied weighting [30, 32] was settled out to downweigh characters according to their homoplasy [27]. This function was enabled (*piwe = ;*) before the reading of the dataset (*proc ‘dataset’;*), and activated with the definition of character partitions (*xpiwe] = 10;*) [33], upon which, the average homoplasy was used to determine the equivalence in constant-weight (*xpiwe&homoplasy;*) [32]. Values of concavities were automatically calculated for each character (xpiwe (*0.25 < 5/12;) [32] and the max tree was configured (*hold 100000;*).

The outgroup was determined (*outgroup ‘species’;*), the internal memory was expanded to accommodate a higher percentage of extra cells initially allocated for sectorial searches (*sect: slack 15;*), and runs were set out (*xmult = hits 10 noupdate nocss replic 10 ratchet 10 fuse 1 drift 5 hold 100 noautoconst keepall;*), to identify the optimal trees through 10 sets (*hits*), each initiated from 10 starting points (*replics*), ensuring the best length is found 10 times independently, using a combination of sectorial searches, drifting, ratchet, and fusing algorithms [31, 101].

The best-scored trees were sought running random addition sequences (RAS), with TBR branch-swapping (*bbreak = tbr;*) using pre-existing trees as the starting point, and between the most parsimonious tree, a strict consensus tree was selected (nelsen *; tchoose {strict};). To calculate the support of the branches, suboptimal trees were randomly searched (*rseed[;*), saving successively an increasing number of them in each step (*hold 1000; sub 1; bbreak: fillonly tbr; hold 10000; sub 2; bbreak: fillonly tbr; hold 20000; sub 20; bbreak: fillonly tbr;*)*.* Posteriorly, combined (absolute and relative) Bremer support was calculated using TBR swapping (*bsupport &;*). Bremer values were printed in the tree (*ttags); ttags;*) which was plotted (*export dataset. tree*;) and edited in Inkscape version 1.3 [9]. Characters were optimized on the consensus tree using TNT ver. 1.6 [29] and Mesquite ver. 3.70 [57, 58] (S1.2).

### Morphometric analyses

Specimens of an undescribed *Ameloblastella* species were subjected to morphometric analysis and compared with the two most closely related species of the genus. A database comprising ten morphometric variables [67] from a total of 32 specimens (11 from Peru, 4 from Argentina, and 17 from Brazil) was utilized. The analysis was conducted using RStudio version 2024.04.2+764 [86] integrated with R version 4.3.1[83]. The dataset was analyzed using a multivariate classification technique, the Linear Discriminant Analysis (LDA), which aims to predict the probability of an entity belonging to a mutually exclusive given class, group, or category. The tidyverse package [102] was employed for data manipulation and visualization, while MASS [84] facilitated LDA functions and caret [54] for easy machine learning workflow.

The dataset was partitioned into two subsets: a training set (60% of the dataset), used to build a predictive model, and a testing set (40% of the data set), used to evaluate the accuracy of that model. The data were parametrized employing estimated parameters, applying the method “center”, which subtracts the mean of the predictor’s data (again from the data in *x*) from the predictor values, and the method “scale”, which divides by the standard deviation [54].

## Results

The findings of this study, derived from a morphological phylogenetic analysis, strongly advocate for a thorough taxonomic revision of *Demidospermus*. This revision requires the establishment of four new genera, the resurrection of two existing genera, and the description of two new species. The specific characters used in the phylogenetic tree are detailed in the provided character list (Supplementary material S1).

### Taxonomic acts

Class Monopisthocotyla Brabec, Salomaki, Kolísko, Scholz, Kuchta, 2023

Order Dactylogyridea Bychowsky, 1937

Family Dactylogyridae Bychowsky, 1933

### *Vancleaveus* Kritsky, Thatcher & Boeger, 1986

Type species: *Vancleaveus janauacaensis* Kritsky, Thatcher & Boeger, 1986 *ex Pterodoras granulosus* (Valenciennes, 1821) (Doradidae) from Brazil, State of Amazonas, Solimões River, Janauacá Lake [51], Argentina, Corrientes, Paraná River [95], Peru, Iquitos, Itaya River [68], Brazil, Castilho, State of São Paulo, Aguapeí River [3], *ex Hoplias aff. malabaricus* (Bloch, 1794), Brazil, States of Paraná and Mato Grosso do Sul, Paraná River [35].

Other species: *Vancleaveus cicinnus* Kritsky, Thatcher & Boeger, 1986, *ex Phractocephalus hemioliopterus* (Bloch & Schneider, 1801) (Pimelodidae), Brazil, State of Amazonas, Solimões River [51], *ex Pimelodus albicans* (Valenciennes, 1840) (Pimelodidae), Argentina, Buenos Aires, de La Plata River [95], *ex Franciscodoras marmoratus* (Lütken, 1874) (Doradidae), Brazil, Três Marias, São Francisco River [88]; *Vancleaveus fungulus* Kritsky, Thatcher & Boeger, 1986, *ex Pseudoplatystoma tigrinum* (Valenciennes, 1840) (Pimelodidae), *ex Pseudoplatystoma fasciatum* (Linnaeus, 1766) (Pimelodidae), Brazil, State of Amazonas, Solimões River, Janauacá Lake [51], *ex P. fasciatum* (Pimelodidae), Peru, Iquitos, Amazon River Basin [68]; *Vancleaveus platyrhynchi* Kritsky, Thatcher & Boeger, 1986 *ex Hemisorubim platyrhynchos* (Valenciennes, 1840) (Pimelodidae), Brazil, State of Amazonas, Solimões River [51], Peru, Iquitos, Amazon River Basin [68]; *Vancleaveus klasseni* Soares, Neto & Domingues, 2018, *ex Hassar orestis* (Steindachner, 1875) (Doradidae) from Brazil, Pará, Xingu River and *Hassar gabiru* Birindelli, Fayal & Wosiacki, 2011 from Brazil, State of Pará, Bacajá River and Xingu River [94].

### *Vancleaveus cicinnus* Kritsky, Thatcher & Boeger, 1986 (Fig. 1)

Type Host: *Phractocephalus hemioliopterus* (Bloch & Schneider, 1801).

**Figure 1:**
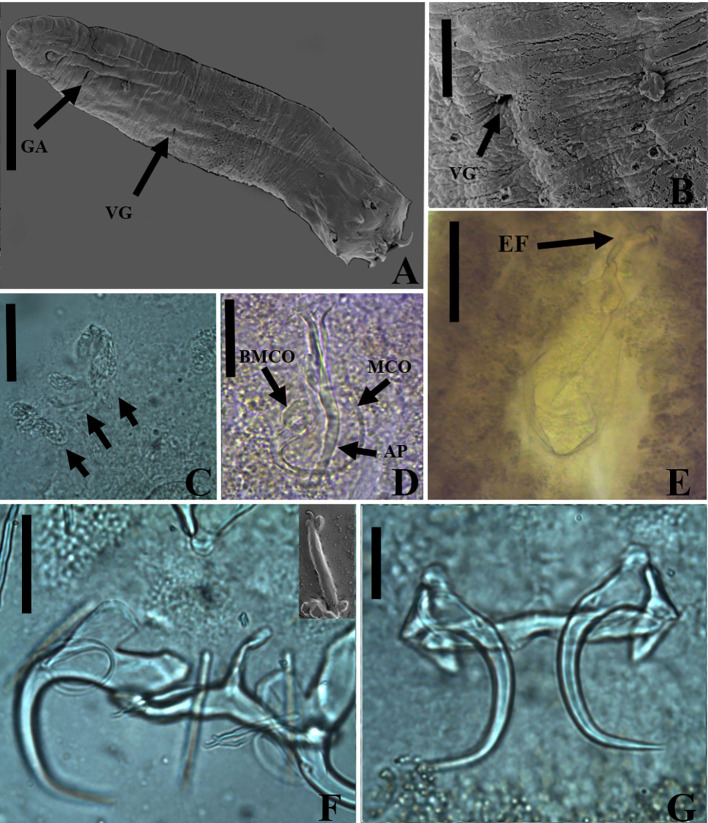
SEM and light photomicrographs of *Vancleaveus cicinnus*, a parasite of *Phractocephalus hemioliopterus*, from the Tapajós river basin, Pará, Brazil. A – SEM of whole worm in ventral view showing the vagina (VG) and genital atrium (GA). B – detail showing the vagina (VG). C – head organs (arrows). D – copulatory complex: male copulatory organ (MCO), base of the MCO (BMCO), and accessory piece (AP). E – egg: egg filament (EF). F – ventral bar and anchors (in detail, the hook by SEM). G – dorsal bar and anchors. Scale bar = 50 µm (C, E), 25 µm (D, F, G).

Type locality: Brazil, State of Amazonas, Solimões River, Janauacá Lake [51].

Other hosts and localities: *Phractocephalus hemioliopterus*, Tapajós River, National Park of Amazonia (−56.299889°; −4.552694°), municipality of Itaituba, State of Pará; Tapajós River, Pimental (−56.264613°; −4.568505°), municipality of Itaituba; and Igarapé Jari (−54.876133°; −2.334050°), Tapajós River Basin, municipality of Santarém, State of Pará [5], *Pimelodus albicans* (Valenciennes, 1840) (Pimelodidae), Argentina, Buenos Aires, de La Plata River [95], *Franciscodoras marmoratus* (Lütken, 1874) (Doradidae), Brazil, Três Marias, São Francisco River [88].

Site of infection: gills.

Present record on: prevalence = 2/4 (50%), 3/4 (75%), 1/2 (50%), mean intensity = 24, 6 and 2, and mean abundance of infection = 1, 4 and 12, from Igarapé Jari, Tapajós River Basin, municipality of Santarém; Tapajós River, National Park of Amazonia, municipality of Itaituba; and Tapajós River, Pimental, municipality of Itaituba, State of Pará, Brazil, respectively.

Present deposited material: voucher 11 slides CHIOC (40613a–b, 40612a–i), voucher 11 slides CHIBB (850L–860L).

#### Measurements

Based on 19 specimens: 9 stained on Gomori’s Trichrome and mounted on Canada balsam, 5 mounted on Gray and Wess’s medium, 5 mounted on Hoyer’s medium.

Body 453 (210–1032; *n* = 19) long, 142 (48–221; *n* = 19) maximum wide. Pharynx 29 (11–65; *n* = 12) in diameter. Haptor 72 (33–124; *n* = 19) long, 104 (51–151; *n* = 19) wide. Ventral anchor 49 (41–62; *n* = 12) long, 30 (21–36; *n* = 12) base; dorsal anchor 42.5 (35–57; *n* = 12) anchor, 19 (11–27; n = 12) base. Ventral bar 55 (37–72; *n* = 17) long, distance between ends 52 (32–71; *n* = 17); dorsal bar 49 (34–68; *n* = 14) long. Hook pair 1 and 3: 27 (12–37; *n* = 21) long, pair 2 and 4: 25 (15–36; *n* = 22) long, pair 5: 18 (11–24; *n* = 8) long, pair 6: 28 (16–35; *n* = 9) long, pair 7: 29 (18–36; *n* = 6) long. MCO 66 (28–94; *n* = 11) long; accessory piece 57 (34–81; *n* = 14) long. Testis 50 (*n* = 10) long, 25 (*n* = 1) wide. Germarium 103 (61–165; *n* = 9) long, 46 (32–79; *n* = 9) wide. Egg 128 (100–145; *n* = 3) long, 39 (33–38; *n* = 3) wide.

#### Remarks

*Vancleaveus* was proposed to accommodate species of Dactylogyridae parasites of siluriform fishes. The genus was defined as exhibiting the following characteristics: overlapping gonads, counterclockwise coiled MCO, ventral vagina, and superficial roots of the dorsal anchor with a fold [64]. The characteristics observed in the specimens of the present study, such as the morphology of the MCO (Fig. 1) and the morphometric data, correspond to those earlier described for *V. cicinnus* [51, 95]. However, some variations were observed in measurements and haptoral structures, mainly bars and anchors. The report of this species in the Tapajós River, in the municipalities of Itaituba and the municipality of Santarém, represents new locality records.

In the present study, specimens of *V. cicinnus* were found with a larger range of width, pharynx, and hook pairs compared to those recovered by Kritsky *et al.* (1986) [51] and Suriano and Incorvaia (1995) [95]. However, in contrast to Suriano and Incorvaia (1995) [95] and in agreement with Kritsky *et al.* (1986) [51], we observed that the fifth pair of hooks in the specimens of the present study were smaller than the remaining hooks. Furthermore, our analysis revealed additional morphological variations not described in the previous descriptions: a medial projection of the ventral bar with a distal dilatation (absent from the original description); a triangular shape of the medial projection of the dorsal bar (compared to the rectangular shape in the original description); longer superficial and deep roots of the ventral bar; and a thinner fold of the dorsal bar. Additionally, we observed variations in the conformation of the superficial root and fold of the dorsal bar, indicating their capability to adjust and enhance attachment to the dorsal anchors. Despite these observed differences, we opted for a more conservative approach and assigned the specimens to *V. cicinnus* in the Tapajós River, municipality of Itaituba, and municipality of Santarém, thereby establishing new locality records.

The most recently described species in the genus, *V. klasseni* [94], exhibits notable morphological differences compared to other species in the genus. One distinct feature is the sinistral position of the vaginal aperture in *V. klasseni*, while in other *Vancleaveus* species, it is ventral. Additionally, the haptoral structures, such as bars and anchors, bear closer resemblance to those of *Ameloblastella* species, although the authors observed a fold at the superficial root of both the ventral and dorsal anchor (only in the dorsal anchors in *Vancleaveus* species). However, the authors did not describe a ligament between the accessory piece and the base of the MCO, which fits with the diagnosis of *Vancleaveus*, as *Ameloblastella* spp. typically exhibit an articulated accessory piece. This set of characters is intriguing as it seems to partially conform to *Ameloblastella*, but also with *Vancleaveus* diagnoses, suggesting the need for further investigation within a phylogenetic framework, mainly because *Vancleaveus* and *Ameloblastella* have been recovered as closely related clades in the phylogenetic inferences based on 28S rDNA [1, 61, 65, 66].

There are some records of species of *Vancleaveus*, but they are difficult to recover because the authors did not deposit vouchers in a museum collection. Some of these studies reported the presence of *V. cicinnus* parasitizing *P. tigrinum* and *P. fasciatum* from Vale do Jamari, Ariquemes, State of Rondonia [14]; *V. cicinnus*, *V. fungulus*, and *V. janauacaensis* in the surubim hybrid (*P. reticulatum* × *P. corruscans*) from fish farms located in State of Mato Grosso do Sul, Central Brazil [44]; and *V. fungulus* parasitizing *P. corruscans* in the Upper Paraná River floodplain, Brazil [98]. This practice of incomplete documentation should be avoided in parasitological studies of South American fishes, as parasite records often represent the sole information available for these species and are frequently geographically fragmented, hampering evaluation of their true geographic distribution and limiting ecological and evolutionary research opportunities.

### *Demidospermus* Suriano, 1983

Syn. *Omothecium*: Kritsky, Thatcher and Boeger [52], 8–12, figs. 1–16; *Paramphocleithrium*: Suriano and Incorvaia [95], 120, figs. 23–29; *Urocleidoides*: part Mendoza-Palmero, Scholz, Mendoza-Franco and Kuchta [68], 495; *Peruanella*: part Cruces *et al.* [22], 593–598.

Type species: *Demidospermus anus* Suriano, 1983 [96], *ex Loricariichthys anus* (Valenciennes, 1835), from Laguna de Chascomús, Buenos Aires, Argentina, *Loricariichthys platymetopon*, from the reservoir of Itaipú Hydroelectric Power Station [21].

Other species: *Demidospermus paranaensis* Ferrari-Hoeinghaus, Bellay, Takemoto & Pavanelli, 2010, *ex Loricariichthys platymetopon* Isbrücker & Nijssen, 1979, Upper Paraná River, State of Paraná, Brazil [24]; *Demidospermus prolixus* Franceschini, Zago, Müller, Francisco, Takemoto & da Silva, 2017, *Demidospermus spirophallus* Franceschini, Zago, Müller, Francisco, Takemoto & da Silva, 2017, *ex Loricaria prolixa* Isbrücker & Nijssen, 1978, Sapucaí-Mirim River, State of São Paulo, Brazil [26]; *Demidospermus rhinelepisi* Acosta, Scholz, Blasco-Costa, Alves & da Silva, 2017, *ex Rhinelepis aspera* Spix & Agassiz, 1829, Aguapeí River, State of São Paulo, Brazil [2]; and *Demidospermus wilveri* Cruces, Santillán, Silvera & Chero, 2024*, ex Loricaria* sp., Madre de Dios River, Madre de Dios, Peru [23].

#### Emended diagnosis

Tegument annulations present, scattered over whole body and peduncle, sometimes inconspicuous; eyes and accessory chromatic granules absent. MCO coiled forming counterclockwise rings, sigmoid or sinuous tube; accessory piece sheath-like, not articulated. Vaginal aperture sinistral, between MCO and germarium; vagina formed by atrium and pre-atrium, only atrium, or only pre-atrium, muscular or sclerotized; vaginal canal sclerotized, curved or sinuous. Intercaecal and tandem gonads, testis posterior to germarium. Peduncle short (from 1 to 7% of total body length). One ventral and one dorsal haptoral bar, both articulated; ventral bar V, U, or W-shaped, with sclerotized narrowing in middle; dorsal bars U, V, or W-shaped, with or without sclerotized narrowing in middle. Haptoral hooks with approximately same size and shape. Parasites of loricariids.

#### Remarks

**Figure 2:**
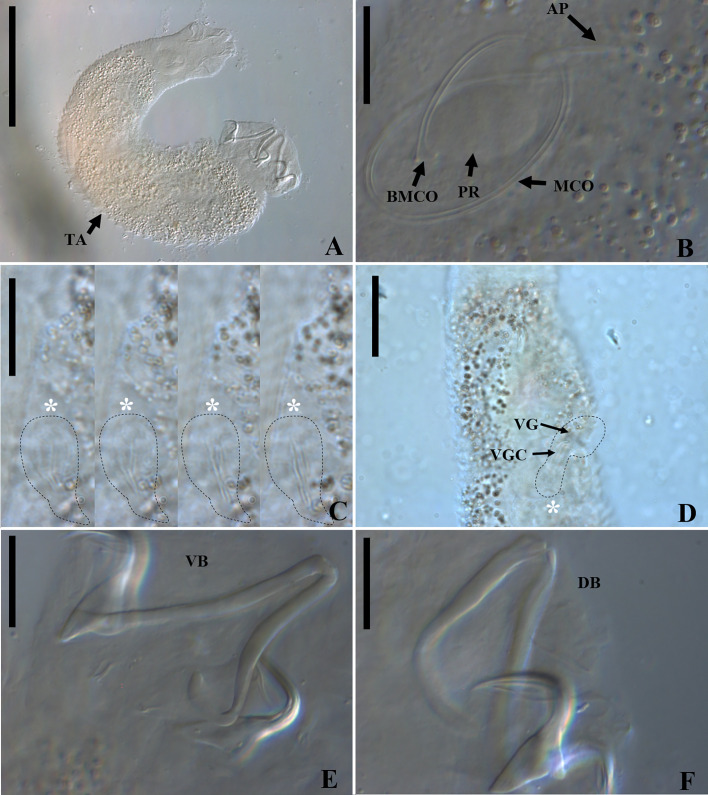
Light photomicrographs of *Demidospermus paranaensis*. A – whole worm in ventral view (CHIOC37255b) showing the tegument annulations (TA). B – MCO: accessory piece (AP); base of the MCO (BMCO); prostatic reservoir (PR) (CHIOC37255b). C – sclerotized distal portion of the deferens duct, observed at varying depths, from left to right, ventral to dorsal: asterisk indicates the same topographical region in D. D – vagina (VG) and vaginal canal (VGC) (CHIOC37255d). E – ventral bar (VB) (CHIOC37255b). F – dorsal bar (DB) (CHIOC37255b). Scale bar = 100 µm (A), 25 µm (B, D–F), 10 µm (C).

**Figure 3:**
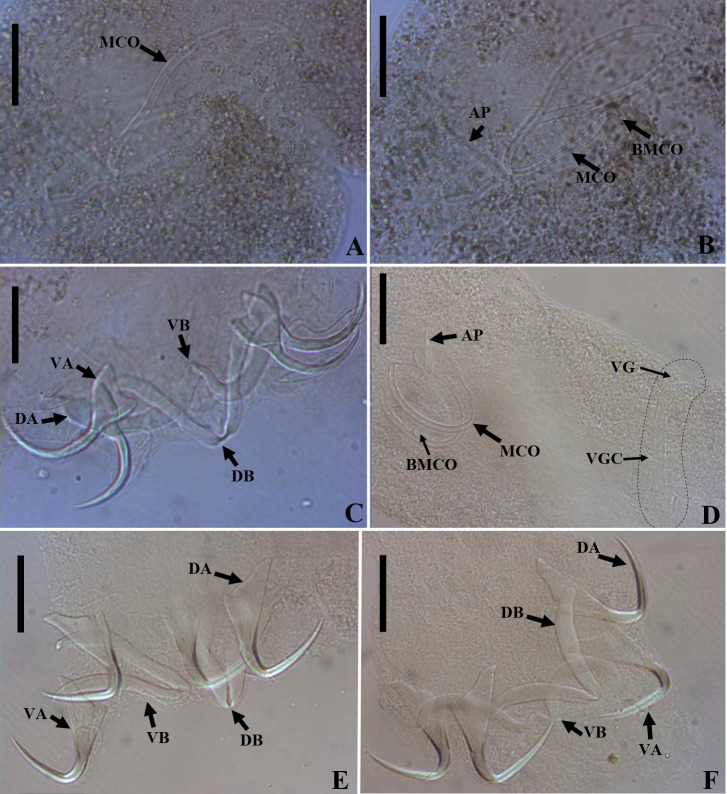
Light photomicrographs of *Demidospermus* spp. *Demidospermus prolixus* (CHIBB 234L). A and B – MCO: accessory piece (AP); base of the MCO (BMCO). C – haptor: ventral anchor (VA) and bar (VB), dorsal anchor (DA) and bar (DB). *Demidospermus spirophallus* (CHIBB 227L). D – reproductive structures: vagina (VG), vaginal canal (VGC). E – haptor. (CHIBB 228L). F – haptor. Scale bar = 15 µm.

**Figure 4:**
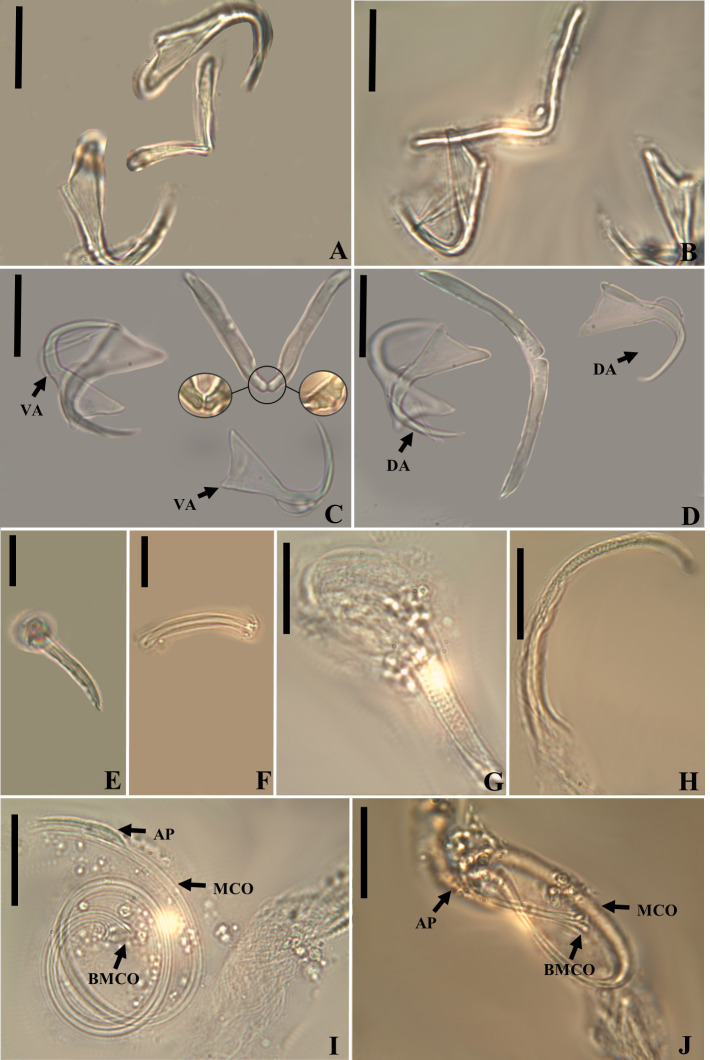
Light photomicrographs of two morphotypes of *Demidospermus anus* parasite of *L. platymetopon* from Upper Paraná River floodplain, Paraná, Brazil. A and B – ventral and dorsal bars and anchors (morphotype A), respectively. C and D – ventral and dorsal bars and anchors (morphotype B), respectively, in detail, the joint point in the ventral bar evidencing the sclerotized narrowing. E – vagina (morphotype A). F – vagina (morphotype B). G – sclerotized distal portion of the deferens duct (morphotype A). H – sclerotized distal portion of the deferens duct (morphotype B). I – MCO: accessory piece (AP); base of the MCO (BMCO) (morphotype A). J – MCO (morphotype B). Scale bar = 100 µm (A), 20 µm (A–D, G–H), 10 µm (E–F, I–J).

**Figure 5:**
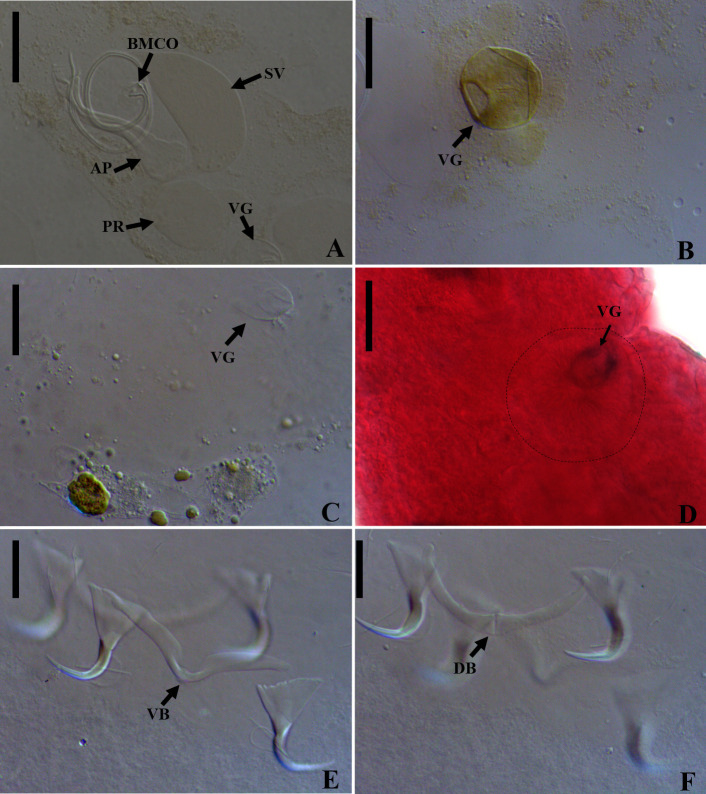
Light photomicrographs of *Demidospermus rhinelepisi*. A – copulatory complex in dorsal view (CHIBB 335L) – MCO: accessory piece (AP); base of the MCO (BMCO); prostatic reservoir (PR); vagina (VG); seminal vesicle (SV) (CHIOC37255b). B and C – vagina (CHIBB 327L, 335L) (VG). D – lateral muscularized seminal receptacle (dotted line) (CHIBB 328L). E and F – ventral (VB) and dorsal bar (DB) (CHIBB 335L). Scale bar = 25 µm (A), 20 µm (C–D), 15 µm (B, E–F).

*Demidospermus stricto sensu* was supported by the following synapomorphies: 1) the presence of tegument annulations; 2) ventral and dorsal bars, both articulated; 3) ventral bar articulated, bowed, V, U, or W-shaped; and 4) with a sclerotized narrowing in the middle (Figs. 2–5, 20). While the articulated, bowed, V, U, or W-shaped ventral bar is shared with species of *Magnanchistrius* n. gen., the tegument annulations, the sclerotized narrowing in the middle of the ventral bar, and the articulated dorsal bar are exclusive synapomorphies of *Demidospermus stricto sensu*.

**Figure 6:**
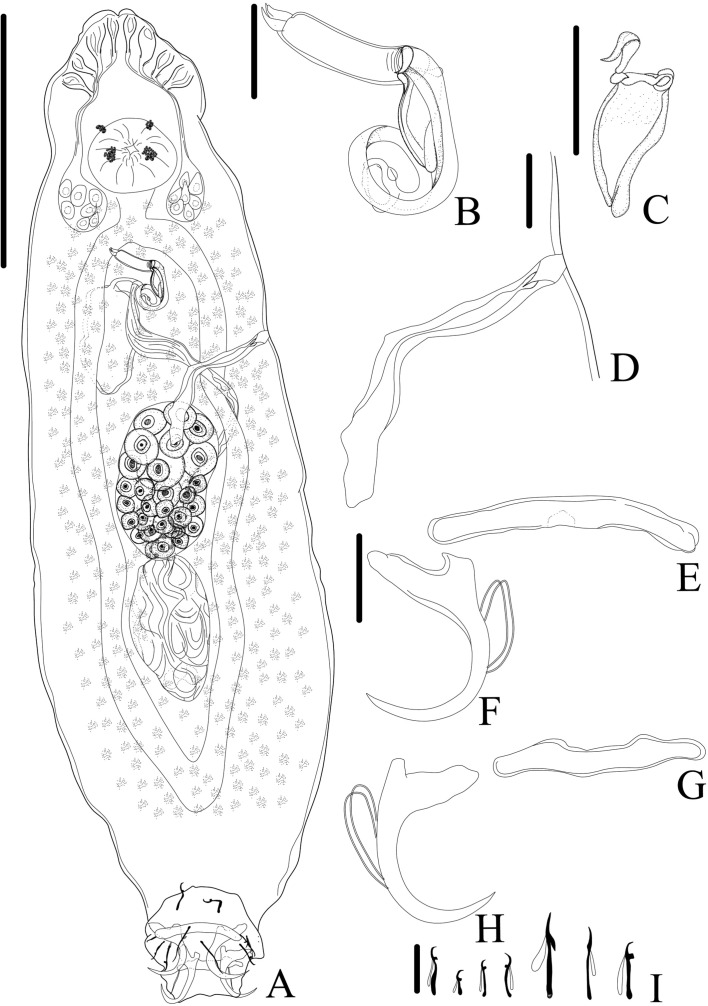
*Urocleidoides amazonensis i. s.* parasite of *Phractocephalus hemioliopterus*, from Tapajós River Basin, Pará, Brazil. A – whole helminth. B – MCO. C – accessory piece. D – vagina. E – ventral bar. F – ventral anchor. G – dorsal bar. H – dorsal anchor. I – hook pairs 1–7. Scale bar = 50 µm (A), 20 µm (C–D), 10 µm (B).

**Figure 7:**
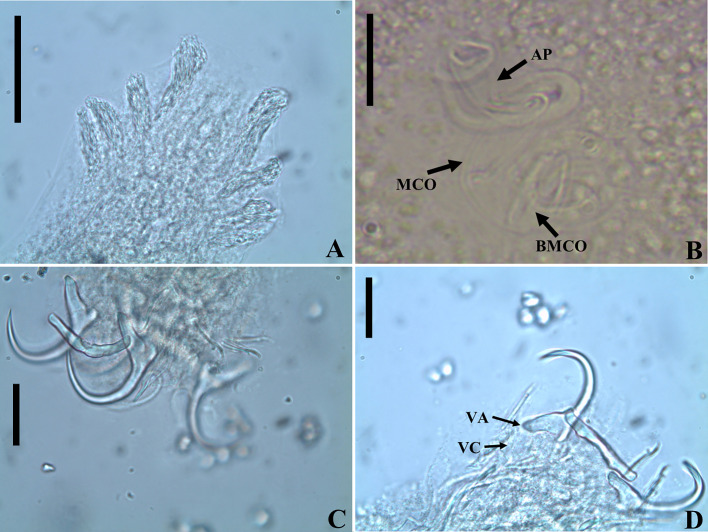
Light photomicrographs of *Urocleidoides amazonensis i. s*., a parasite of *Phractocephalus hemioliopterus*, from Tapajós River Basin, Pará, Brazil. A – anterior region of the head showing four pairs of head organs. B – copulatory complex, MCO, base of MCO (BMCO), accessory piece (A). C – ventral bar and anchor. D – dorsal bar and anchors. Scale bar = 15 µm.

**Figure 8:**
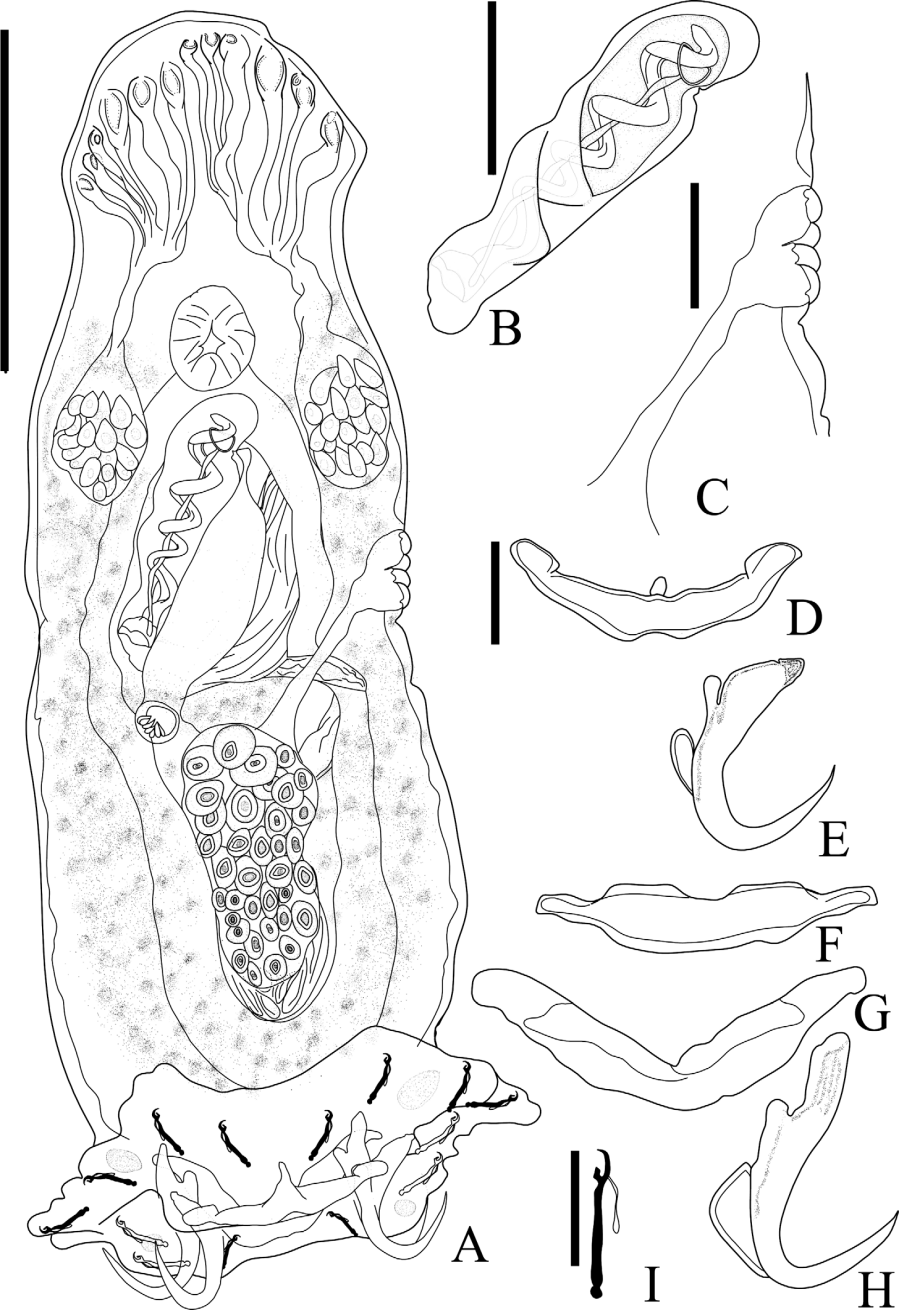
*Ameloblastella sakulocirra* n. sp. (ventral view), a parasite of *Phractocephalus hemioliopterus*, from the Tapajós river basin, Pará, Brazil. A – whole helminth. B – MCO, with part of the sac removed to improve visualization. C – vagina. D and E – ventral bar and anchor. F and G – dorsal bar in different specimens and views. H – dorsal anchor, I – hook. Scale bar = 100 μm (A), 25 μm (C), 20 μm (B), 15 μm (D – I).

**Figure 9:**
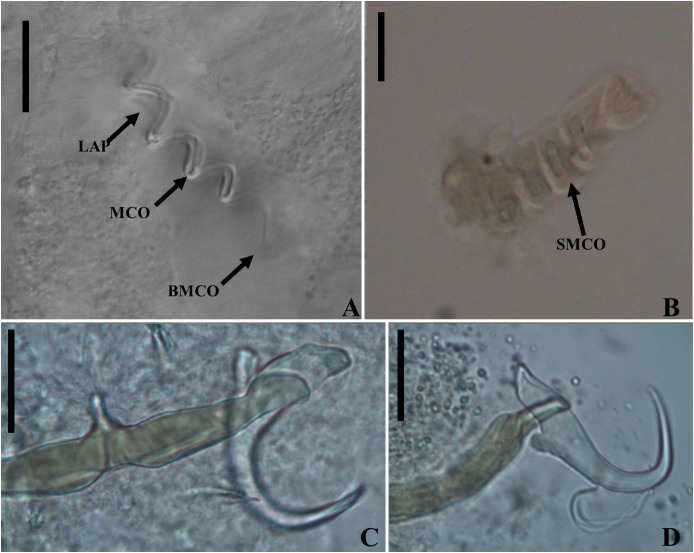
Light photomicrographs of *Ameloblastella sakulocirra* n. sp., a parasite of *Phractocephalus hemioliopterus*, from the Tapajós river basin, Pará, Brazil. A and B – copulatory complex: male copulatory organ (MCO), base of the MCO (BMCO), ligament of the accessory piece (LAP) to the base of the MCO, and the sac that surrounds the MCO (SMCO). C – ventral bar and anchor. D – dorsal bar and anchor. Scale bar = 15 µm.

**Figure 10:**
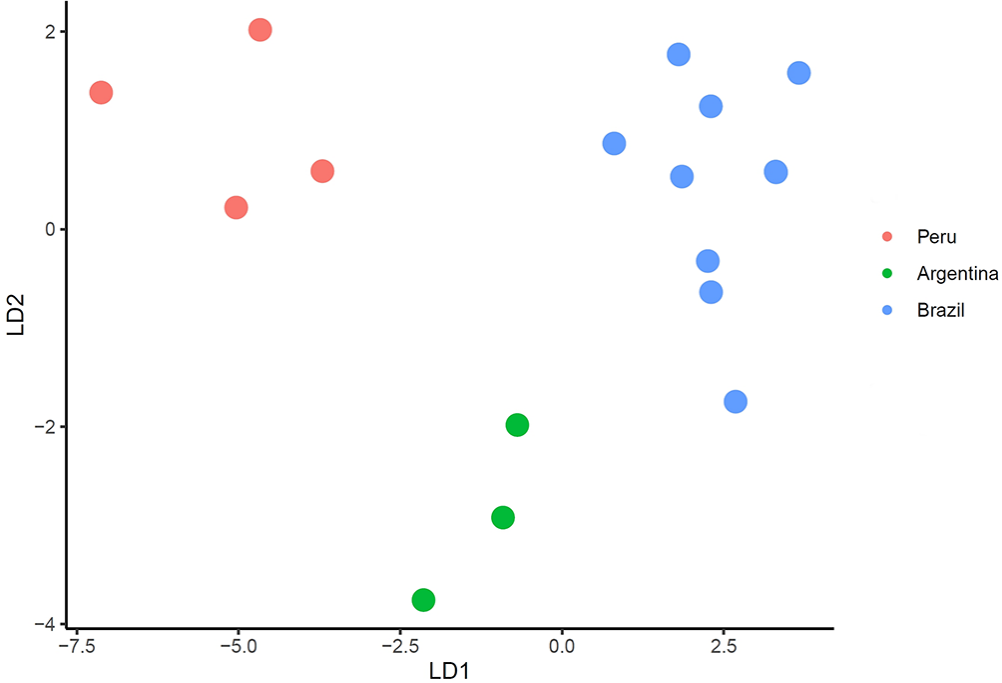
LDA plot with the first (LD1) and second (LD2) linear discriminants for *Ameloblastella martinae* (Peru), *Ameloblastella sakulocirra* n. sp. (Brazil), and *Ameloblastella* sp. (Argentina).

**Figure 11:**
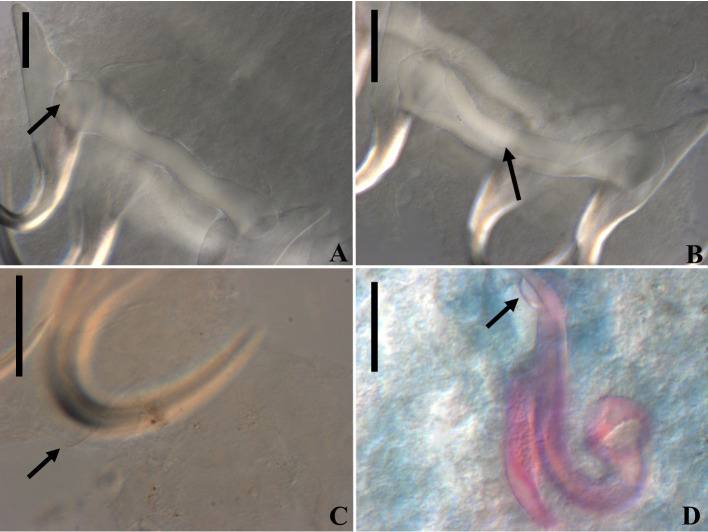
Light photomicrographs of *Rhabdolachosus ceccarellii* comb. n. A and B – ventral and dorsal bar (CHIOC 37325d; arrow indicating the grooves). C – Hook (CHIOC 37325d; indicated by arrow). D – MCO (CHIOC 37324c; extension of accessory piece indicated by the arrow). Scale bar = 15 µm.

**Figure 12:**
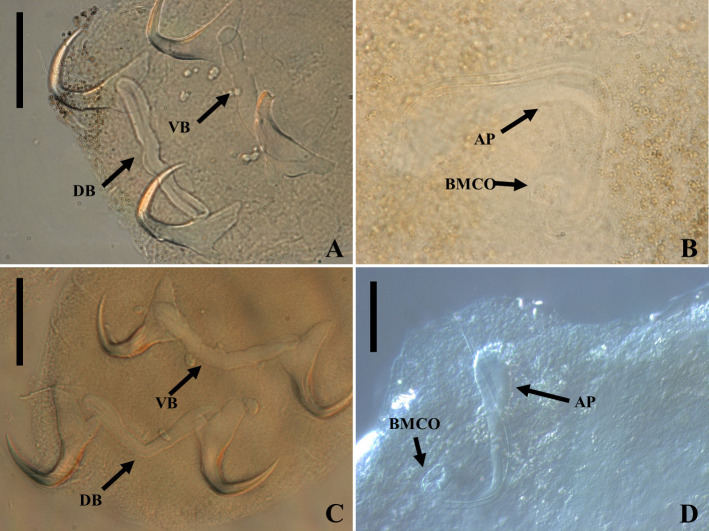
Light photomicrographs of *Rhabdolachosus brachyplatystomae* comb. n. A and B – haptoral and copulatory complex (CHIOC 37322c; AP – accessory piece; BMCO – base of the MCO). C and D – haptoral and copulatory complex (CHIOC 37322d). Scale bar = 40 µm.

**Figure 13:**
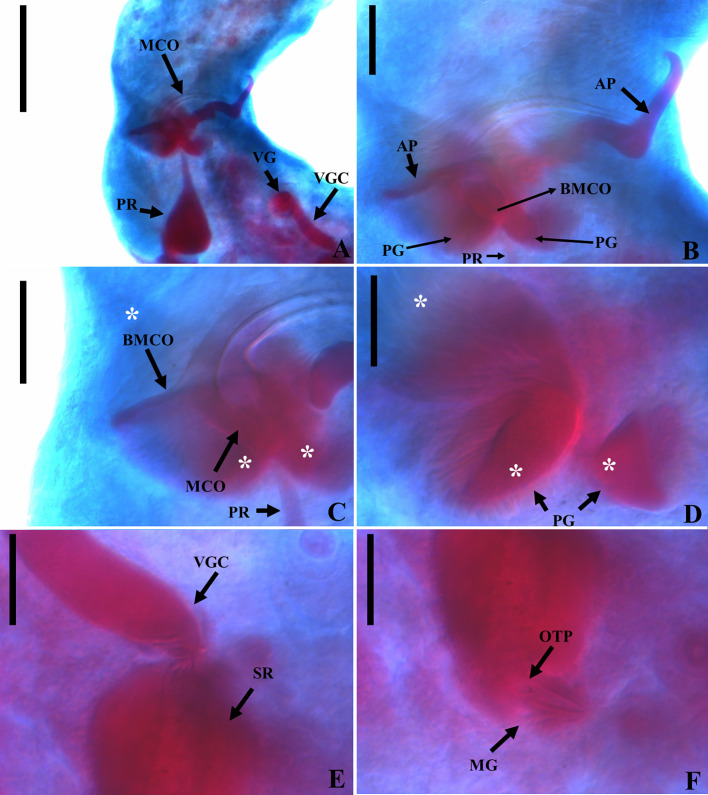
Light photomicrographs of *Rhabdolachosus araguaiaensis* comb. n. (CHIOC 37327) A – Reproductive organs: male copulatory organ (MCO), prostatic reservoir (PR); vagina (VG); vagina canal (VGC). B – MCO: accessory piece (AP); base of the MCO (BMCO); prostatic glands (PG). C – MCO: asterisk indicates the same topographical region in D. D – PG. E – distal part of VGC opening into the seminal receptacle (SR). F – ootype (OTP) surrounded by the Mehlis’ gland (MG). Scale bar = 40 µm (A), 20 µm (B–C), 10 µm (D–F).

**Figure 14:**
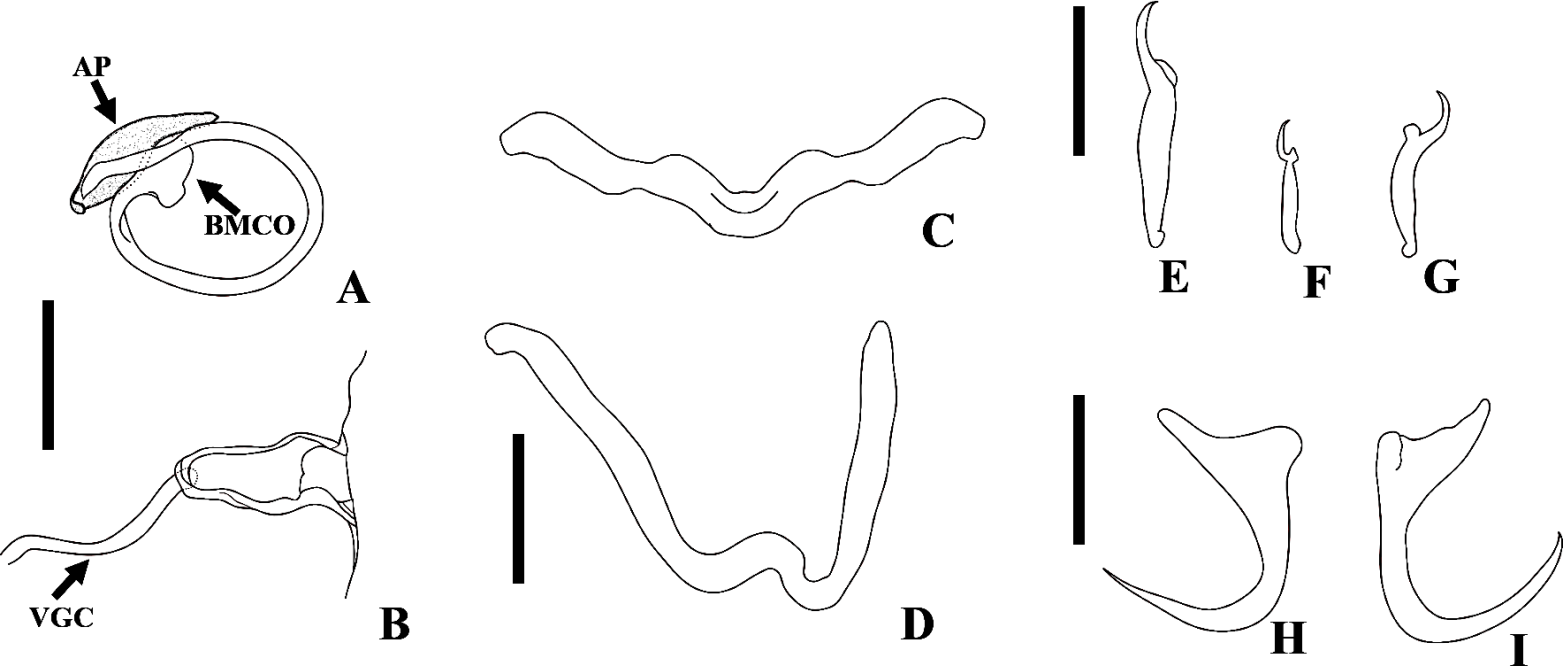
Drawings of *Martorellius paravalenciennesi* comb. n. (USNM 1382349) parasite of *Pimelodus clarias* from de La Plata River, Buenos Aires, Argentina. A – copulatory complex in ventral view – MCO: accessory piece (AP); base of the MCO (BMCO). B – vagina: vagina canal (VGC). C – composite drawing of the dorsal bar. D – ventral bar. E – hook 1. F – hook 2. G – hook 7. H – ventral anchor. I – dorsal anchor. Scale bar = 15 µm.

**Figure 15:**
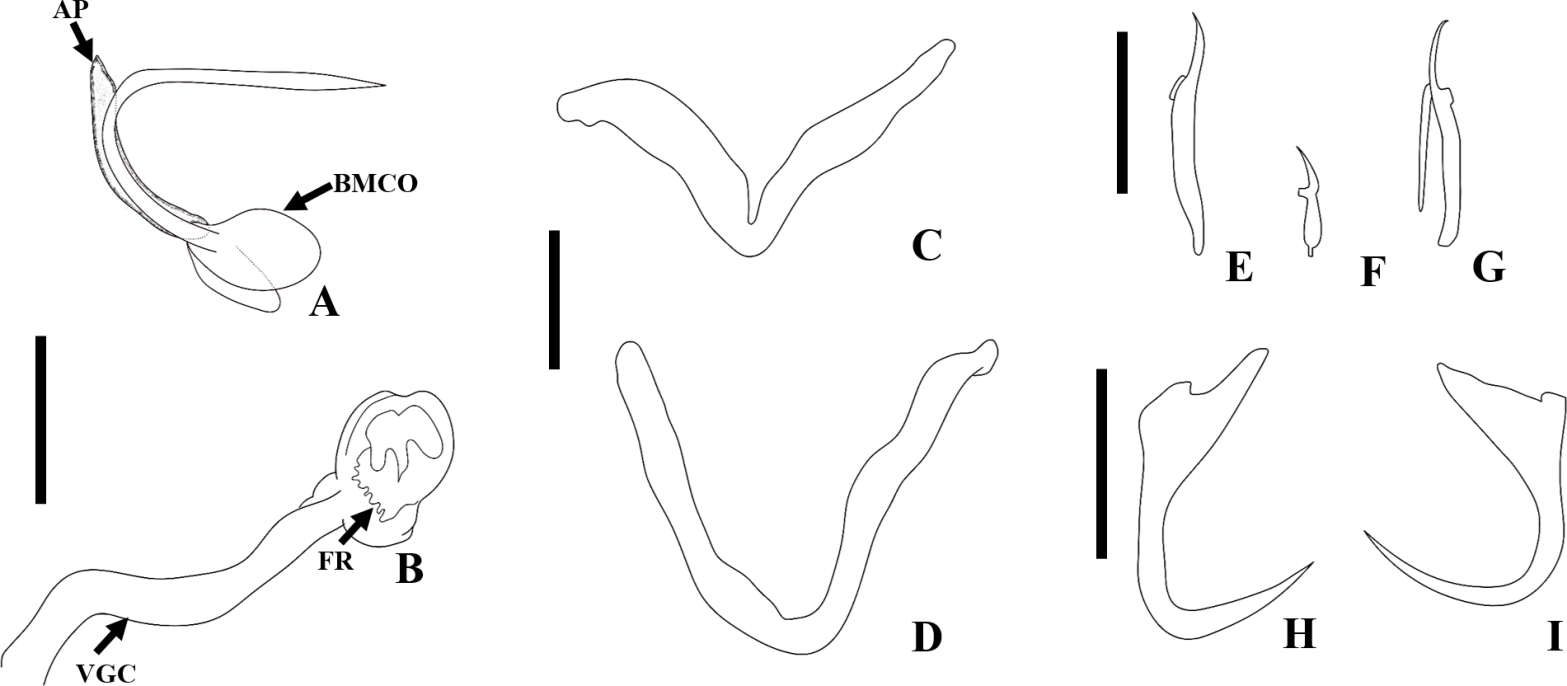
Drawings of *Martorellius valenciennesi* comb. n. (USNM 1382361) parasite of *Parapimelodus valenciennis* from de La Plata River, Buenos Aires, Argentina. A – copulatory complex in ventral view – MCO: accessory piece (AP); base of the MCO (BMCO). B – vagina: vagina canal (VC), fringes of vaginal atrium (FR). C – composite drawing of the dorsal bar. D – ventral bar. E – hook 1. F – hook 2. G – hook 7. H – ventral anchor. I – dorsal anchor. Scale bar = 15 µm.

**Figure 16:**
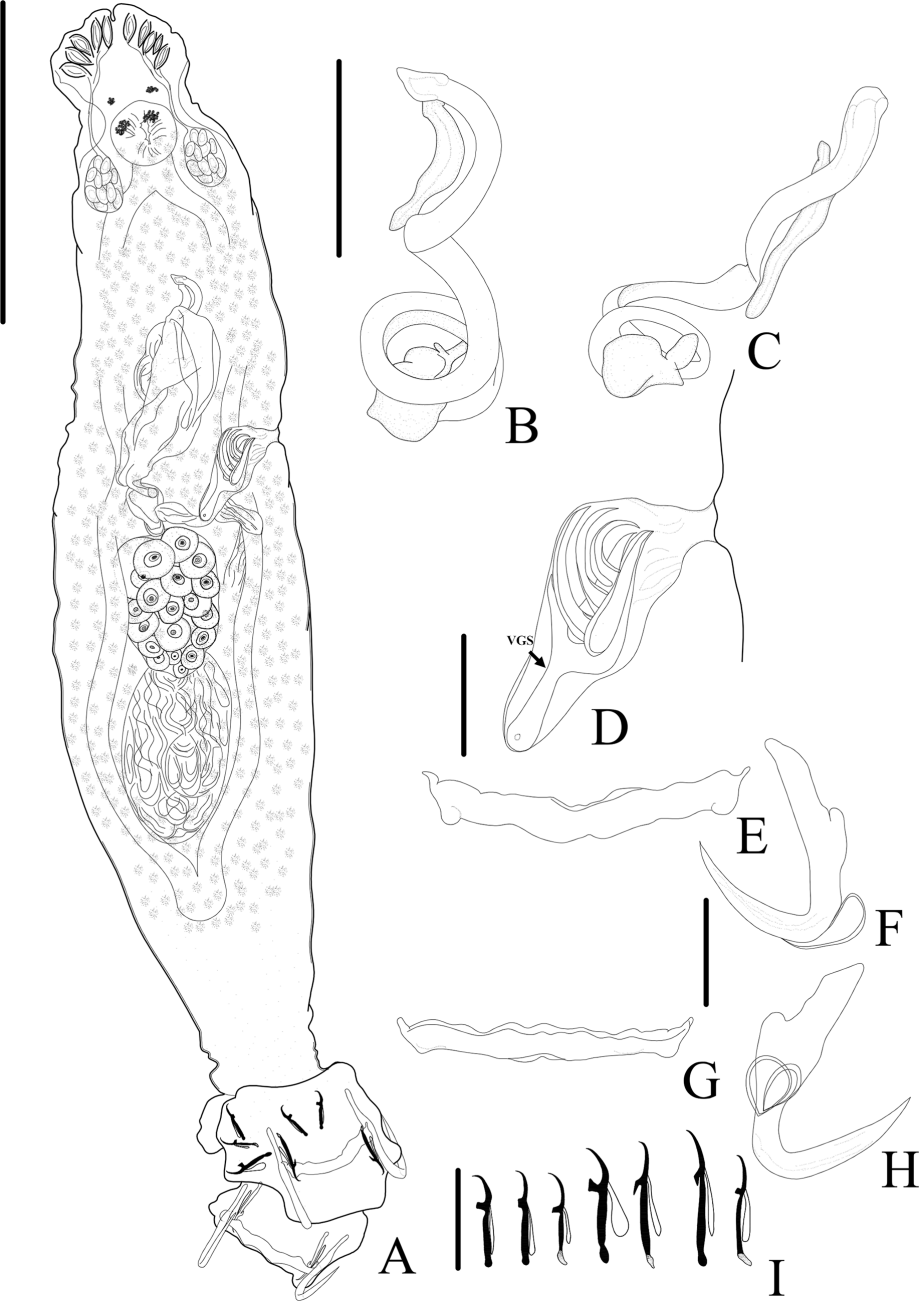
*Sicohencotyle antoniomaiai* n. gen. n. sp. (ventral view), a parasite of *Phractocephalus hemioliopterus*, from the Tapajós river basin, Pará, Brazil. A – Whole helminth. B and C – MCO in, respectively, ventral and dorsal view. D – vagina, highlighting the vaginal sclerite (VGS). E and F – ventral bar and anchor. G and H – dorsal bar and anchor, I – hooks pairs 1 to 7, from left. Scale bar = 150 µm (A), 25 µm (B, C), 15 µm (D, I), 20 µm (E–H).

**Figure 17:**
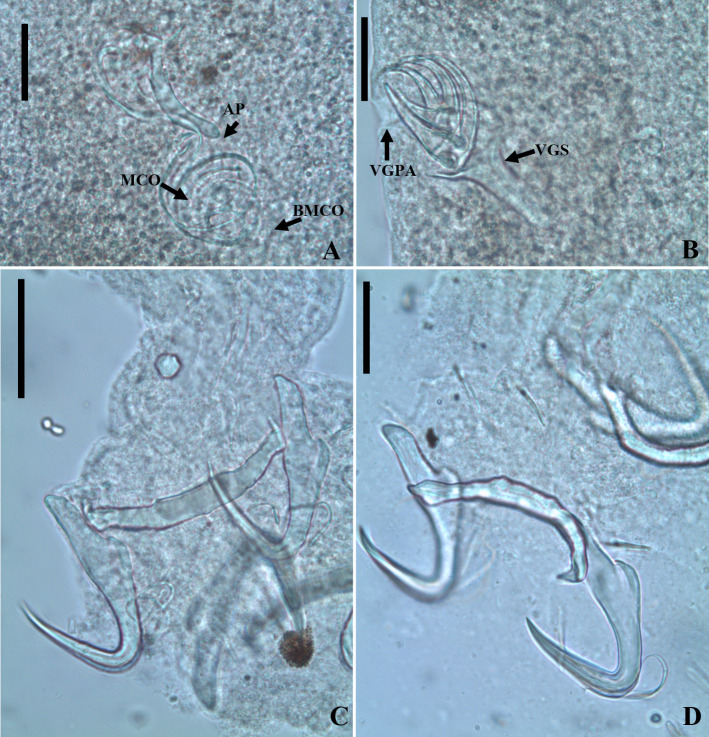
Photomicrographs of *Sicohencotyle antoniomaiai* n. gen. n. sp., a parasite of *Phractocephalus hemioliopterus*, from the Tapajós river basin, Pará, Brazil. A – MCO in dorsal view: base of the MCO (BMCO), accessory piece (AP). B – vagina in dorsal view: vaginal pre atrium (VGPA), vaginal sclerite (VGS). C – ventral bar and anchor. D – dorsal bar and anchor. Scale bar = 25 µm (A, C–D), 15 µm (B).

**Figure 18:**
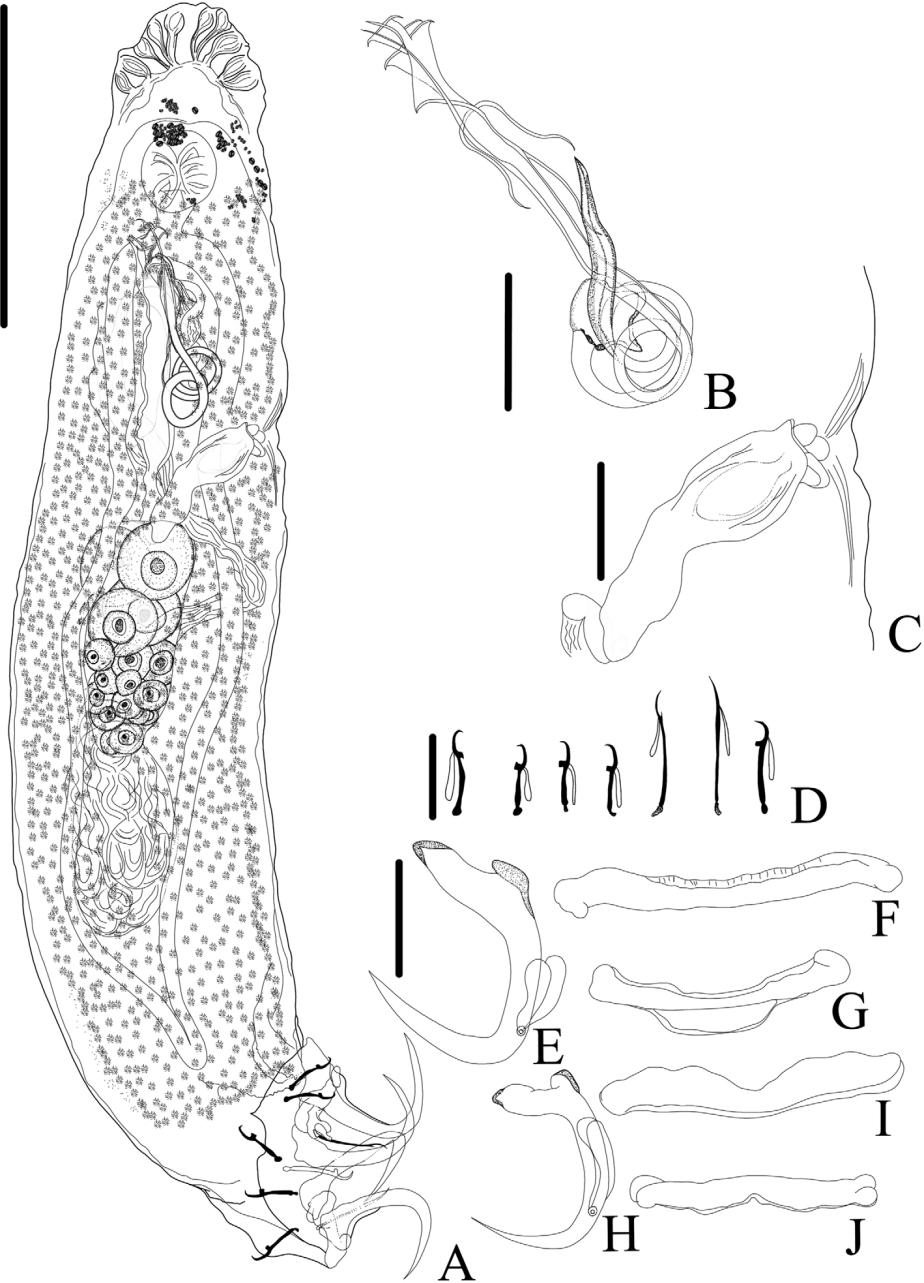
*Sicohencotyle catus* comb. n. (ventral view), a parasite of *Phractocephalus hemioliopterus*, from the Tapajós river basin, Pará, Brazil. A – whole helminth. B – MCO in ventral view. C – vagina. D – hooks pairs 1–7. E – ventral anchor. F and G –ventral bar. H – dorsal anchor. I and J – dorsal bar. Scale bar = 100 µm (A), 15 µm (B–J).

**Figure 19:**
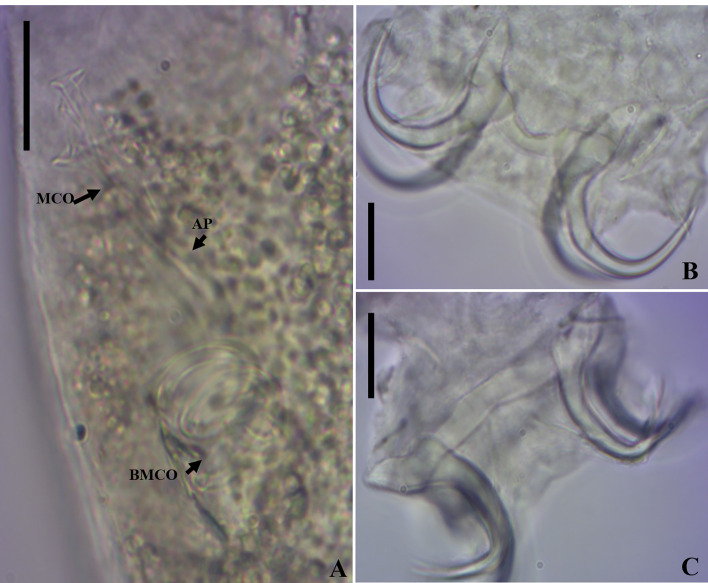
*Sicohencotyle catus* comb. n. (ventral view), a parasite of *Phractocephalus hemioliopterus*, from the Tapajós river basin, Pará, Brazil. A – MCO, base of the MCO (BMCO), accessory piece (AP). B – ventral bar and anchors. C – dorsal bar and anchors. Scale bar = 20 µm.

**Figure 20:**
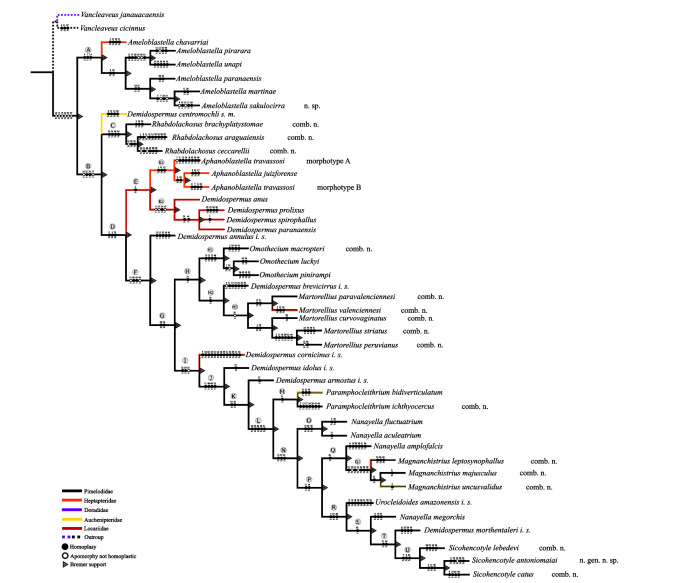
Strict consensus tree (Length = 354, CI = 0.329, RI = 0.607) of the morphological phylogenetic inference for the *Demidospermus*-like species. The numbers above and below branches referto the characters and transformation series, respectively. Siluriform hosts were reconstructed through unordered parsimonious character history tracing searching.

In certain instances, as evidenced in specimens of *D. paranaensis* (Fig. 2), a parasite of *L. platymetopon* from the Upper Paraná River in Brazil, the tegument annulations can exhibit conspicuous visibility [24]. However, in specimens of *D. anus* (USNM 1382363), collected from the type host and locality (*i.e.*, *L. anus* from Laguna de Chascomús, Buenos Aires, Argentina), this trait can also be observed, but discreetly. Tegument annulations were also observed by Franceschini *et al.* (2018) [26] in *D. anus* specimens collected by Cohen and Kohn (2008) [21], from *L. platymetopon* in the reservoir of the Itaipú Hydroelectric Power Station, Paraná, Brazil. However, the authors did not note this characteristic in *D. anus* specimens collected by themselves from the Upper Paraná River (upstream of the Itaipú reservoir), in *L. platymetopon*. Notably, this is the same host as studied by Cohen and Kohn (2008) [21] and Ferrari-Hoeinghaus (2010) [24], as well as the same locality as Ferrari-Hoeinghaus *et al.* (2010) [24], although the Cohen and Kohn (2008) [21] collection site is approximately 200 km downstream. Accessing the same specimens of *D. anus* (CHIBB 237L–243L) studied by Franceschini *et al.* (2018) [26], we also failed to observe this structure. Considering that the specimens examined by Cohen and Kohn (2008) [21] and Ferrari-Hoeinghaus *et al.* (2010) [24] were fixed in formalin at a concentration of 1:4000 and that tegument annulations are more prominently visible in their specimens, it is likely that this fixation method enhances the visibility of this structure, as suggested by Franceschini *et al.* (2018) [26], as fixation in formalin does not typically lead to the visualization of this structure in the majority of dactylogyrid species.

At first glance, it appears that the ventral and dorsal bars consist, each of them, of two articulated bars in *Demidospermus sensu stricto* species. However, a more detailed analysis reveals that the ventral bar possesses a distinctively sclerotized structure in its medial portion, which acts as a connecting element for the other sections of the bar. This structure resembles an additional, diminutive connective sclerite, providing a sense of continuity to the bar. In contrast, some species of the genus lack this structure in the dorsal bar. Interestingly, despite their distinct features, both the ventral and dorsal bars resist separation *via* proteolytic proteins, indicating their integrity as unique, articulated units (Fig. 4).

Following the description of the type species of the genus, *D. anus*, in *L. anus* from Buenos Aires, Argentina, Cohen and Kohn (2008) [21] reported this species in *L. platymetopon* from Itaipú Reservoir, Brazil; however, this record was potentially erroneous according to Ferrari-Hoeinghaus *et al.* (2010) [24]. These authors described *D. paranaensis* parasitizing *L. platymetopon* from the Upper Paraná River Basin, State of Paraná, Brazil. Ferrari-Hoeinghaus *et al.* (2010) [24] differentiated this species from *D. anus* based on haptoral sclerite dimensions and MCO morphology, as well as the presence of tegument annulations, which were reportedly absent in *D. anus.* Table 2 shows that there are noticeable differences in the dimensions of haptoral structures between *D. anus* and *D. paranaensis.* The length of the bars is particularly distinct, while other dimensions are similar in both species. Furthermore, differences were observed in the morphology of anchors between these two species.

**Table 2:** Morphometric and ecological variables of specimens of *Demidospermus stricto sensu*.

	*D. anus*	*D. paranaensis*	*D. anus*	*D. spirophallus*	*D. prolixus*	*D. rhinelepisi*
	Suriano (1983) [96]	Ferrari-Hoeinghaus *et al.* (2010) [24]	Franceschini *et al.* (2018) [26]	Franceschini *et al.* (2018) [26]	Franceschini *et al.* (2018) [26]	Acosta *et al.* (2018) [2]
BL	675 (500–680)	344 (249–431; *n* = 19)	356 (319–392; *n* = 3)	880 (670–1225; *n* = 8)	747 (416–987; *n* = 7)	559 (394–814; *n* = 10)
BGW	175 (120–180)	94 (49–99; *n* = 20)	65 (55–75; *n* = 3)	125 (89–177; *n* = 12)	119 (109–130; *n* = 7)	121 (100–136; *n* = 10)
PD	–	25 (18–36; *n* = 17) long	28 (24–32; *n* = 4)	43 (33–53; *n* = 12) long	37 (34–48; *n* = 7) long	41 (37–47; *n* = 10) long
		21 (14–32; *n* = 17) wide		45 (24–60; *n* = 12) wide	31 (25–58; *n* = 7) wide	39 (36–47; *n* = 10) wide
HL	–	45 (36–60; *n* = 20)	48 (46–52; *n* = 3)	106 (63– 135; *n* = 10)	78 (60–103; *n* = 7)	58 (53–66; *n* = 8)
HW	–	59 (42–80; *n* = 20)	71 (69–73; *n* = 3)	136 (84–206; *n* = 10)	170 (152–205; *n* = 7)	75 (69–80; *n* = 8)
VAL	27 (26–28; *n* = 2)*	25 (17–50; *n* = 15)	22 (20–23; *n* = 3)*	33 (32–34; *n* = 7)*	37 (36–38; *n* = 3)*	29 (27–32; 15)
VAW	15 (14–16 *n* = 2)	14 (9–30; *n* = 15)	11 (11–12; *n* = 3)*	18 (17–19; *n* = 7)*	14 (12–15; *n* = 3)*	20 (18–22; 15)
DAL	29 (28–30; *n* = 2)*	25 (12–60; *n* = 14)	24 (21–26; *n* = 3)*	37 (36–39; *n* = 7)*	37 (36–38; *n* = 3)*	
DAW	14 (12–17; *n* = 2)*	13 (9–30; *n* = 14)	13 (10–14; *n* = 3)*	20 (19–21; *n* = 7)*	15 (14–15; *n* = 3)*	
VBL	93 (92–94; *n* = 3)*	81 (61–91; *n* = 13)	51 (50–53; *n* = 3)*	88 (85–92; *n* = 7)*	83 (75–96; *n* = 3)*	
DBL	96 (94–98; *n* = 3)*	80 (59–87; *n* = 12)	43 (41–45; *n* = 13)*	80 (70–84; *n* = 7)*	71 (65–82; *n* = 3)*	
Hook	15 (*n* = 2) pairs 1–4, 6–7	15 (14–16; *n* = 12)	11 (10–12; *n* = 8)	13 (9–15; *n* = 15)*	16 (14–16; *n* = 5)* pairs 1–3, 5–7	14 (14–15; 15)
	18 (18–19; *n* = 2) pair 5*				19 (18–21; *n* = 2)* pair 4	
MCO	235 (210–255)	136 (130–147; *n* = 3)*	152 (143–156; *n* = 5)	212 (193–230; *n* = 11)	223 (210–234; *n* = 7)	134 (129–140; *n* = 4)*
APL	30 (25–35)	25 (22–27; *n* = 2)*	31 (30–33; *n* = 5)	53 (49–57; *n* = 5)	36 (32–38, *n* = 7)	50 (44–53; *n* = 15)
GL	–	40 (19–55; *n* = 6)	27 (21–34; *n* = 2)*	52 (40–65; *n* = 4)	–	115 (99–129; *n* = 4)
GW	–	31(19–42); *n* = 7)	19 (19–20; *n* = 2)*	59 (21–86; *n* = 4)	–	54 (44–60; *n* = 4)
TL	–	54 (46–72; *n* = 7)	53 (46–61; *n* = 2)*	254 (*n* = 1)	138 (113–164; *n* = 2)	53 (45–61; *n* = 3)
TW	–	29 (17–38; *n* = 7)	28 (23–33; *n* = 2)*	150 (*n* = 1)	64 (54–74; *n* = 2)	33 (26–38; *n* = 1)
Host	*L. anus*	*L. platymetopon*	*L. platymetopon*	*L. prolixa*	*L. prolixa*	*R. aspera*
Locality	Buenos Aires, Argentina	Paraná, Brazil	Paraná, Brazil	São Paulo, Brazil	São Paulo, Brazil	São Paulo, Brazil

* Measurements acquired in this study for standardization purposes. Body length (BL), body greatest width (BGW), pharynx diameter (PD), haptor long (HL), haptor wide (HW), ventral anchor length (VAL), ventral anchor base width (VAW), dorsal anchor length (DAL), dorsal anchor base width (DAW), ventral bar length (VBL), dorsal bar length (DBL), mean of marginal hooks (Hook), male copulatory organ total length (MCO), accessory piece length (APL), germarium length (GL), germarium width (GW), testis length (TL), testis width (TW).

*Demidospermus anus* described by Suriano (1983) [96] and *D. paranaensis* described by Ferrari-Hoeinghaus *et al.* (2010) [24] exhibit anchors with underdeveloped superficial roots. These characteristics were observed in the specimens of *D. anus* analyzed in the current study (USNM 1382363). However, some specimens of *D. paranaensis* contained well developed superficial roots (CHIOC37255 d–e) as well as specimens with underdeveloped superficial roots (CHIOC37255 b). Likewise, among the specimens of *D. anus* analyzed by Franceschini *et al.* (2018) [26], it is possible to observe specimens with anchors showing underdeveloped superficial roots (CHIBB 237L) and specimens whose anchors exhibit relatively well developed superficial roots (CHIBB 239L–240L). This characteristic was also observed in the specimens analyzed in the present study. In Figures 4A and 4B, a specimen (referred to as morphotype A) with anchors containing relatively well developed superficial roots can be observed. On the other hand, in Figures 4C and 4D, there is a specimen in which the anchors do not possess well developed superficial roots (referred to as morphotype B).

We found differences in the size and shape of the MCO between *D. anus* and *D. paranaensis*. The measurement reported by Ferrari-Hoeinghaus *et al.* (2010) [24] only indicates the space occupied by the MCO in the body, not the complete MCO measurement. We conducted new measurements for *D. paranaensis*, and the differences between the two species are presented in Table 2. Specimens of *D. anus* from Argentina have a larger MCO than those from Brazil, as well as *D. paranaensis* from Brazil. The MCOs of *Demidospermus stricto sensu* species can be sinuous, sigmoid, or forming rings (Figs. 2–5). These rings can take an elliptical shape in some species, as in *D. anus* and *D. paranaensis*, with varying numbers of ellipses. However, we observed two morphotypes concerning these rings of the MCO.

Specimens of *D. anus* from the type locality and host (USNM 1382363) possess a sinuous MCO, or coiled with loosely formed rings. Suriano (1983) [96] also characterized the MCO of *D. anus* as coiled with loosely formed rings. However, among the specimens of *D. anus* provided by Franceschini *et al.* (2018) [26], there is a specimen characterized by an MCO coiled with loosely formed rings (CHIBB 237L), but also another with MCO coiled with tightly formed rings (CHIBB 240L). The same can be noted for the specimens of *D. paranaensis*, since there is one specimen with a coiled MCO with loosely formed rings (CHIOC 37255b) and others with a coiled MCO with tightly formed rings (CHIOC37255 d–e). Among the specimens we analyzed, there are some with MCOs forming tightly coiled rings (Fig. 4I, morphotype A), and others with sinuous MCO or with MCO forming loosely coiled rings (Fig. 4J, morphotype B). These morphotypes align with the morphotypes of the anchors presented earlier. In other words, specimens with anchors exhibiting a relatively well developed superficial root tend to have MCOs with tightly coiled rings (morphotype A), while specimens with anchors showing a poorly developed superficial root tend to have sinuous or loosely coiled MCOs (morphotype B). Furthermore, specimens of *D. anus* from Argentina exhibit an MCO with a maximum of 1.5 ellipses preceding the accessory piece, while specimens of *D. anus* and *D. paranaensis* from Brazil, with an MCO with tightly coiled rings can display up to 2.

In the specimens of *D. anus* from the type locality and host (USNM 1382363), there was not a vaginal atrium; instead, the sclerotized vaginal canal opens directly into the body wall, giving rise to a sclerotized vaginal pre-atrium. But the remaining species of the genus present a vaginal atrium. In *D. prolixus*, the vaginal atrium is muscular, and in *D. paranaensis* and *D. spirophallus*, it is sclerotized. However, among the specimens of *D. anus* examined in the present study from Brazil, there is a muscular vaginal pre-atrium, as demonstrated by Cohen and Kohn (2008) [21]. Nonetheless, there is also morphological variability in the distal portion of the vaginal canal of the specimens of *D. anus* from Brazil (serrated *versus* smooth vaginal canal), which corroborates the same morphotypes described above (Figs. 4E–4F).

The characteristics observed in specimens of *D. anus* and *D. paranaensis* suggest the presence of a population of *D. anus* in Argentina, with an MCO that is sinuous or coiled into loosely formed rings, absence of a vaginal atrium, anchors containing relatively poorly developed superficial roots and dorsal bar without sclerotized narrowing in the middle. Meanwhile, in Brazil, two morphotypes have been identified, but it remains unclear whether they correspond to *D. anus* or *D. paranaensis*. One morphotype resembles *D. anus* specimens from Argentina, while the other is distinguished by an MCO coiled into tightly formed rings and anchors with relatively more developed superficial roots. However, both morphotypes share a vaginal atrium and a sclerotized narrowing in the middle of the dorsal bar. These variations may correspond to intraspecific variations, as Cohen and Kohn (2008) [21] suggested. However, further meticulous studies encompassing both morphological and molecular data, as proposed by Franceschini *et al.* (2018) [26], will be imperative to test this hypothesis and resolve this taxonomic impasse.

The species comprising *Demidospermus stricto sensu* are morphologically very similar, especially regarding *D. anu*s, *D. paranaensis*, and the recently described species, *D. wilveri*. The latter is retained within the genus because it shares the morphological characteristics supporting the genus, particularly those related to the bars and MCO, and it parasitizes loricariids, the only host group reported for these species so far. Like *D*. *anus*, *D. wilveri* appears not to exhibit the sclerotized narrowing in the middle of the dorsal bar, a structure present in the other species of the genus described to date. Cruces *et al.* (2024) [23] differentiated *D. wilveri* from *D. anus* by the absence of a self-fertilization tube (present in *D. anus*). This structure seems to be the distal portion of the deferens duct, which, in *D. anus* and *D. paranaensis*, is sclerotized. Cruces *et al.* (2024) [23] distinguished *D. wilveri* from *D. paranaensis* based on the absence of tegument annulations, present in *D. paranaensis*. As discussed earlier, this structure may or may not be visualized, apparently depending on how the helminths were fixed. Additionally, Cruces *et al.* (2024) [23] considered the short and straight vaginal canal in *D. wilveri*, contrasting with the long, sigmoid vaginal canal in *D. paranaensis*. However, in one of the specimens of *D. paranaensis* (CHIOC37255d), the vaginal canal is relatively short, containing only curved ends (Fig. 2D). Therefore, the only characteristic that seems to differentiate *D. wilveri* from the other species, especially the most similar ones, *D. anus* and *D. paranaensis*, is the distal portion of its MCO, which is spatulate or spoon-shaped, while the other species have a contiguous or acute distal portion of the MCO.

### *Sedis mutabilis* and *incertae sedis*

*Demidospermus centromochi* Mendoza-Franco & Scholz, 2009, *ex Centromochlus heckelii* (de Filippi, 1853) (Auchenipteridae) from Iquitos, Peru [64], should be considered *sedis mutabilis*. *Demidospermus annulus* Marcotegui & Martorelli, 2011, *ex Parapimelodus valenciennesi* (=*Parapimelodus valenciennis*) (Lütken, 1874) (Pimelodidae) from Argentina, Buenos Aires, Baía de Samborombón [59]; *Demidospermus brevicirrus* Mendoza-Palmero, Scholz, Mendoza-Franco & Kuchta, 2012 *ex Pimelodus* sp. (Pimelodidae) from Iquitos, Peru [68]; *Demidospermus cornicinus* Kritsky & Gutierrez, 1998 *ex Bergiaria westermani* (=*Iheringichthys westermani*) (Lütken, 1874) (Pimelodidae) from Buenos Aires, Argentina [48]; *Demidospermus idolus* Kritsky & Gutierrez, 1998, *Demidospermus armostus* Kritsky & Gutierrez, 1998, *ex Pimelodus albicans* (Valenciennes, 1840) (Pimelodidae) from Buenos Aires, Argentina [48]; *Urocleidoides amazonensis* Mizelle & Kritsky, 1969, *ex Phractocephalus hemioliopterus* (Pimelodidae) from Amazonas, Brazil [72]; *Demidospermus mortenthaleri* Mendoza-Palmero, Scholz, Mendoza-Franco & Kuchta, 2012, *ex Brachyplatystoma juruense* (Boulenger, 1898) (Pimelodidae) from Iquitos, Peru [68]; *Demidospermus osteomystax* Tavernari, Takemoto, Lacerda & Pavanelli, 2010, *ex Auchenipterus nuchalis* (Spix & Agassiz, 1829) (Auchenipteridae) from Maranhão, Brazil [99]; *Demidospermus tocantinensis* Cohen, Justo, Gen & Boeger, 2020, *ex Auchenipterus nuchalis* (Auchenipteridae) from Tocantins, Arraias River, Brazil [19], *Demidospermus doncellae* Morey, Rojas, Dávila, Chu & De Pina, 2024, *ex Pseudoplatystoma punctifer* (Castelnau, 1855) (Pimelodidae), from Belén market, Loreto, Peru [75], *Demidospermus bifurcatus* Justo, Martins & Cohen, 2024, *Demidospermus juruaensis* Justo, Martins & Cohen, 2024 and *Demidospermus takemotoi* Justo, Martins & Cohen, 2024, *ex Ageneiosus inermis* (Linnaeus, 1766), from Juruá River, State of Acre, Brazil [45], should be considered *incertae sedis*.

#### Remarks

The species considered here as *sedis mutabilis* and *incertae sedis* arose in a polytomic clade, or did not cluster within any clade that would allow the identification of synapomorphies supporting their relationship with the other analyzed species, or were not analyzed in this study. Therefore, a more conservative approach was deemed more appropriate, rather than proposing numerous monotypic genera, as this could potentially create further taxonomic confusion in a group that has long been in need of a revision of its morphological boundaries. In some of these species, based on morphology, homology of certain characters was suspected and they were therefore analyzed. However, our initial suspicion of homology was not supported by the phylogenetic inferences presented here (Fig. 20).

For example, *D. cornicinus* and *D. idolus*, with their similar MCO morphology and the presence of dilated hooks pair 1, resemble species of the newly proposed genus *Martorellius*. However, these species did not cluster together (Fig. 20) and the main distinguishing characters were the modifications in the points of hooks pairs 5 and 6, present in *Martorellius* species. In addition, *D. annulus* appears to belong to this morphological group, but remains classified as *incertae sedis* since it neither grouped with *Demidospermus sensu stricto* species nor formed a clade with other taxa that would allow its synapomorphies to be determined.

*Demidospermus armostus* is another species that did not align with our hypothesized phylogenetic relationships. While it shares similar MCO morphology and accessory piece articulation with species of *Paramphocleithrium*, it is suggested (Fig. 20) that these characteristics evolved independently in these species. *Paramphocleithrium* spp. can be distinguished from *D. armostus* by their urn-shaped vaginal atrium (*versus* cup-shaped in *D. armostus*).

Certain species, namely *D. centromochi* and *D. brevicirrus*, posed significant challenges in formulating hypotheses regarding their phylogenetic relationships due to their distinct characteristics deviating from other *Demidospermus*-like species. *Demidospermus centromochi* exhibits a distinct accessory piece comprised of two articulated segments, while *D. brevicirrus* showcases highly unique morphology of the MCO, resembling a rough cylindrical tube. These species appear to have evolved as distantly related entities, as indicated by the phylogenetic analysis (Fig. 20).

Other species, such as *D. mortenthaleri i. s.* and *U. amazonensis i. s*., bear a resemblance to the species within the *Sicohencotyle* n. gen. and *N. megorchis* in terms of the overall morphology of haptoral structures and the shape of the MCO. Notably, *U. amazonensis i. s.* shares the coiled MCO with counterclockwise rings with these species, while *D. mortenthaleri i. s*. typically exhibits a coiled MCO, it can also be observed with an uncoiled MCO. The non-sclerotized vaginal canal, found in species of *Sicohencotyle* n. gen. and *N. megorchis*, is also shared by *U. amazonensis i. s.* Additionally, a curved or sinuous vaginal canal is present in *U. amazonensis i. s*., *N. megorchis*, and *D. mortenthaleri i. s*. However, both *D. mortenthaleri i. s*. and *U. amazonensis i. s*. display unique combinations of characteristics that differentiate them from these species, making it challenging to extract a synapomorphy uniting them.

Like *D. osteomystax, D. tocantinensis* and *D. doncellae* were not included in the current phylogenetic analysis. These species are distinguished by possessing a dextral vaginal aperture, not typical of *Demidospermus*-like species, indicating the potential need to establish a new genus. While *D. osteomystax* exhibits both ventral and dorsal bars articulated, resembling those of *Demidospermus sensu stricto*, *D. tocantinensis* has only the dorsal bar articulated, similar to *Aphanoblastella travassosi* (morphotype A), *D. annulus i. s.*, *Martorellius striatus* comb. n., and *D. cornicinus i. s*. On the other hand, the bars in *D. doncellae* are unarticulated and slightly straight, resembling those of species in *Rhabdolachosus* n. gen. Therefore, even though these species share a dextral-sided vaginal opening, they may correspond to different lineages. It is premature in this study to allocate them to any other genus proposed here or to erect a new genus for their placement. Therefore, further investigations are required to elucidate their relationships with other dactylogyrids. Thus, we consider them to be *incertae sedis*, pending further taxonomic clarification.

The recently described species *Demidospermus juruaensis* Justo, Martins & Cohen, 2024, *Demidospermus bifurcatus* Justo, Martins & Cohen, 2024, and *Demidospermus takemotoi* Justo, Martins & Cohen, 2024 – parasites of the auchenipterid *Ageneiosus inermis* (Linnaeus, 1766) from State of Acre, Brazil [45] – should, in our view, be regarded as *incertae sedis*. These species do not parasitize loricariids, and their morphological traits are incongruent with *Demidospermus stricto sensu*. Notably, the vaginal position in at least two of these species remains uncertain, further complicating their taxonomic placement. Moreover, none of these species appear to possess articulated bars, a key morphological feature of *Demidospermus stricto sensu*, reinforcing the need for a reassessment of their classification.

### *Urocleidoides amazonensis i. s.* Mizelle & Kritsky, 1969 (Figs. 6, 7)

Type host: *Phractocephalus hemioliopterus* (Pimelodidae) [72].

Type locality: Amazonas River Basin [72].

Site of infection: Gills.

Prevalence: 4/4 (100%), 3/4 (75%), 2/2 (100%), mean intensity = 16, 3 and 6, and mean abundance of infectio*n* = 16, 4 and 6, from Igarapé Jari, Tapajós River Basin, municipality of Santarém; Tapajós River, National Park of Amazonia, municipality of Itaituba; and Tapajós River, Pimental, municipality of Itaituba, State of Pará, Brazil, respectively.

Present record: *Phractocephalus hemioliopterus*, Igarapé Jari (−54.876133°; −2.334050°), Tapajós River Basin, municipality of Santarém; Tapajós River, National Park of Amazonia (−56.299889°; −4.552694°), municipality of Itaituba; and Tapajós River, Pimental (−56.264613°; −4.568505°), municipality of Itaituba, State of Pará, Brazil.

Deposited material: voucher 6 ZUEC PLA (206–211), 5 MZUSP (8082a–c, 8083, 8084), 5 CHIOC (40604–40605, 40606a-b, 40607), 3 CHIBB (874L–876L).

#### Measurements

Based on 5 specimens: 4 mounted in Hoyer’s medium, and 1 stained and mounted in Gray & Wess’s medium.

Body 518 (371.5–606; *n* = 5) long; 156.5 (139–175.5; *n* = 3) maximum wide. Pharynx 36 (31.5–43; *n* = 3) in diameter. Haptor 57 (51–61.5; *n* = 5) long, 74 (68.5–83; *n* = 5) wide. Ventral anchor 31 (29.5–34.5; *n* = 4) long, 18.5 (16–21, 5; *n* = 4) base wide; dorsal anchor 30 (28.5–31.5; *n* = 4) long, 19 (17.5 – 20; *n* = 4) base wide. Ventral bar 38.5 (33.5–44.5; *n* = 3) long, distance between ends 36 (28.5–43; *n* = 3); dorsal bar 44 (35–49.5; *n* = 4) long. Hook pair 1: 17 (15.5–19; *n* = 8) long, pair 2, 3 and 4: 15 (13.5–15; *n* = 5) long, pair 5 and 6: 22 (19–24; *n* = 3) long, pair 7: 17 (16–19; *n* = 3) long. MCO 111.5 (85–138; *n* = 2) long, proximal ring 17 (13–21; *n* = 2) in diameter; accessory piece 39.5 (29–50; *n* = 2) long. Testis 88 (73.5–111; *n* = 4) long, 66 (88–65.5; *n* = 4) wide. Germarium 91 (82–107; *n* = 4) long, 53.5 (38.5–66; *n* = 4) wide.

#### Remarks

Among the *Urocleidoides* species, parasites of siluriform fishes once considered *incertae sedis*, *Ameloblastella* (syn. *Urocleidoides*) *chavarriai* (Price, 1938), *Ameloblastella* (syn. *Urocleidoides*) *mamaevi* (Kritsky & Thatcher, 1976), *Aphanoblastella* (syn. *Urocleidoides*) *travassosi* (Price, 1938), *Aphanoblastella* (syn. *Urocleidoides*) *mastigatus* (Suriano, 1986), *Aphanoblastella* (syn. *Urocleidoides*) *robustus* (Mizelle & Kritsky, 1969), *Philocorydoras* (syn. *Urocleidoides*) *corydori* (Molnar, Hanek & Fernando, 1974) and *Philocorydoras* (syn. *Urocleidoides*) *margolisi* (Molnar, Hanek & Fernando, 1974) have been recombined in other genera based on morphological similarities [50, 51, 69, 104]. Other species like *Nanayella* (syn. *Urocleidoides*) *megorchis* (Mizelle & Kritsky, 1969), *Urocleidoides carapus* Mizelle, Kritsky & Crane, 1968, and *Urocleidoides gymnotus* Mizelle, Kritsky & Crane, 1968, were either reclassified into another genus [1] or determined to belong to *Urocleidoides* [89] based on phylogenetic inferences from molecular characters. However, species such as *Urocleidoides amazonensis and Urocleidoides catus* still lack comprehensive studies to elucidate the natural history of these groups.

The main characteristics observed in the specimens of *U. amazonensis i.s*. that support the original description [72] include: the presence of four eyes, morphologically similar anchors with a slightly larger ventral anchor, and similar bars, though the ventral bar is larger than the dorsal bar (Figs. 6, 7). While Mizelle and Kritsky (1969) [72] observed specimens with overlapping or tandem gonads, we only observed specimens with tandem gonads. According to these authors, the MCO of *U. amazonensis i.s*. has one or two rings with ornamentation at the terminal end; however, the distal ring may resemble a twist (Figs. 6, 7) and not a proper ring. The accessory piece with a sigmoidal distal portion [72] was observed in this study to be deltoid with a sinuous distal portion. Even though the authors did not observe a vagina in the *U. amazonensis i.s*. specimens, we did observe a weekly sclerotized vagina opening on the left side of the body. Despite the noted differences, the specimens examined in this study possess bars and anchors identical to those of *U. amazonensis i.s*., and in the specimens studied, which exhibited less distended MCO, it was possible to diagnose the MCO morphology described by Mizelle and Kritsky (1969) [72]. Mizelle and Kritsky (1969) [72] also observed that the species closest to *U. amazonensis i.s*. were *U*. *catus* and *Nanayella* (syn. *Urocleidoides*) *megorchis* due to these species sharing modifications in hooks of pairs 5 and 6. Our phylogenetic inference (Fig. 20) supports the close relationship between these species, although it does not provide a definitive resolution for *U. amazonensis i.s.*

### *Ameloblastella* Kritsky, Mendoza-Franco & Scholz, 2000

Syn. *Cleidodiscus*: part Price [81], 408, pl. I, figs. 4–6; *Urocleidoides*: part Molnar *et al.* [73], 919, fig. 9, part Kritsky and Thatcher [53], 131–132, figs. 13–20; *Vancleaveus*: part Suriano and Incorvaia [95], 116, figs. 8–14; *Pseudovancleaveus*, França *et al.* [25], 29–31, fig. 3.

Type species: *Ameloblastella chavarriai* (Price, 1938) *ex Rhamdia rogersi* (Regan, 1907) (Heptapteridae) from San Pedro Montes de Oca, Costa Rica [81], *ex Rhamdia guatemalensis* (Günther, 1864) from Ixin-há Cenote, Yucatan, Mexico [50], *ex Rhamdia quelen* (Quoy & Gaimard, 1824) from Cumuto River near Coryal, Trinidad [73].

Other species: *Ameloblastella mamaevi* (Kritsky & Thatcher, 1976), *ex Cephalosilurus zungaro* (=*Zungaro zungaro*) (Humboldt, 1833) (Pimelodidae), Colombia [53]; *Ameloblastella platensis* (Suriano & Incorvaia, 1995), *ex Pimelodus clarias maculatus* (=*Pimelodus maculatus*) Lacépède, 1803 (Pimelodidae), Argentina [95]; *Ameloblastella paranaensis* (França, Isaac, Pavanelli & Takemoto, 2003), *ex Iheringichthys labrosus* (Lütken, 1874) (Pimelodidae), Upper Paraná River, State of Paraná, Brazil [25]; *Ameloblastella unapi* Mendoza-Franco & Scholz, 2009, *ex Calophysus macropterus* (Pimelodidae), Amazon River Basin, Peru [64]; *Ameloblastella satoi* Monteiro, Kritsky & Brasil-Sato, 2010, *ex P. maculatus* (Pimelodidae), São Francisco River, State of Minas Gerais, Brazil [74]; *Ameloblastella edentensis* Mendoza-Franco, Mendoza-Palmero & Scholz, 2016, *ex Hypophthalmus edentatus* Spix e Agassiz, 1829 (Pimelodidae), Nanay River, Peru [63], *ex Hypophthalmus marginatus* Valenciennes, 1840 (Pimelodidae), Tocantins River, Maranhão, Brazil [70]; *Ameloblastella peruensis* Mendoza-Franco, Mendoza-Palmero & Scholz, 2016, *ex Hypophthalmus* sp. (Pimelodidae), Iquitos, Peru [63], *ex Hypophthalmus marginatus* Valenciennes, 1840 (Pimelodidae), Tocantins River, State of Maranhão, Brazil [70]; *Ameloblastella formatrium* Mendoza-Franco, Mendoza-Palmero e Scholz, 2016, *ex* undetermined pimelodid, Nanay River, Peru [63], *ex Pimelodella avanhandavae* Eigenmann, 1917 (Heptapteridae), and *Hemisorubim platyrhynchos* (Valenciennes, 1840) (Pimelodidae), State of São Paulo, Brazil [3]; *Ameloblastella unapioides* Mendoza-Franco, Mendoza-Palmero & Scholz, 2016, *ex Sorubim lima* (Bloch & Schneider, 1801) (Pimelodidae), Iquitos, Peru [63]; *Ameloblastella amazonica* Negreiros, Tavares-Dias & Pereira, 2019, *ex. Pimelodus blochii* Valenciennes, 1840 (Pimelodidae), State of Acre and Iaco Rivers, Brazil [76]; and *Ameloblastella pirarara* Mathews, Domingues, Maia, Silva, Adriano & Aguiar, 2021, *ex Phractocephalus hemioliopterus* (Pimelodidae), Tapajós River, Brazil [61]; *Ameloblastella prima* Meneses, Justo & Cohen, 2023, *ex Pimelodina flavipinnis* Steindachner, 1876 (Pimelodidae), Tocantins River, State of Maranhão, Brazil [70].

#### Emended diagnosis

Tegument annulations absent. Eyes absent; accessory chromatic granules in cephalic and anterior trunk regions possibly present. MCO coiled, with counterclockwise rings or corkscrew-like coil, with at least two coils preceding accessory piece; sac of MCO absent or present; accessory piece sheath-like, with or without lateral lobe or formed by two sclerotized pieces, all proximally or distally articulated with base of MCO through a ligament. Vaginal aperture sinistral, between MCO and germarium level; vagina formed by atrium and pre-atrium or just atrium; vaginal atrium and pre-atrium muscular or sclerotized; vaginal canal sclerotized or not sclerotized, curved or sinuous or with loops. Intercecal and overlapped gonads, testis dorsal to germarium. Peduncle absent, short or medium-sized (*i.e.*, from 1 to 15% compared to the body total length). One ventral and one dorsal haptoral bar, not articulated, slightly straight or V, U, and W-shaped; ventral bar with a medio-anterior or medio-posterior projection. Haptoral hooks with approximately the same size and shape. Parasites of heptapterids and pimelodids.

#### Remarks

*Ameloblastella* has been repeatedly recognized as a monophyletic genus [1, 61, 65, 66]. Consistent with previous findings, our research corroborates this natural history, highlighting the significance of the coiled MCO, with at least two rings in the MCO preceding the accessory piece, and the accessory piece articulating to its base through a ligament. It is worth noting that these character states, despite their significance, may represent homoplastic synapomorphies (Fig. 20). However, the combination of these characteristics is sufficient to distinguish *Ameloblastella* from any other analyzed genera.

Within *Ameloblastella*, certain species possess anchors with evenly curved shafts and tips (*A. chavarriai, A. mamaevi, A. satoi, A. edentensis, A. peruensis, A. martinae, A. pirarara*, *Ameloblastella sakulocirra* n. sp. and *A. prima*), while others exhibit reasonably straight shafts and tips (*A. platensis, A. paranaensis, A. unapi, A. formatrium, A. unapioides,* and *A. amazonica*), forming an approximately 90-degree angle at their intersection. Although such characters cannot be phylogenetically informative, they are taxonomically relevant. On the other hand, some species display more than ten rings in the MCO and loops in the vaginal canal. Mathews *et al.* [61] suggested that these characteristics could represent synapomorphies for *A. unapi* and *A. pirarara*, which our inference supported (Fig. 20). Among other known species of the genus, the number of MCO rings does not exceed six, and vaginal canal loops are absent.

The recent inclusion of *A. martinae* in *Ameloblastella* has introduced a novel morphotype of the MCO, characterized by a corkscrew-like shape, adding to the existing polymorphism within the genus. While this characteristic may suggest that these species pertain to a separate lineage, it has been classified within the genus, supported by evidence from previous molecular phylogenetic analyses [67], and by our current morphological inference (Fig. 20).

Notably, the accessory piece of *A. chavarriai* exhibits a unique form, consisting of two sclerotized pieces joined proximally [50, 81], setting it apart from other species within the genus. Furthermore, this species is the only one described outside of South America, parasitizing a heptapterid host, whereas the remaining species were found in South America and primarily parasitize pimelodid hosts, except for *A. formatrium*, which was also reported in a heptapterid host [3].

### *Ameloblastella sakulocirra* n. sp. (Figs. 8–10)

urn:lsid:zoobank.org:act:78CB7AE8-A830-4430-98DA-5F757878EC97

Type host: *Phractocephalus hemioliopterus* (Bloch & Schneider, 1801), “redtail catfish”.

Type locality: Tapajós River, National Park of Amazonia, municipality of Itaituba, State of Pará (−56.299889°; −4.552694°), Brazil.

Other localities: Tapajós River, Pimental (−56.264613°; −4.568505°), municipality of Itaituba; and Igarapé Jari, Tapajós River Basin, municipality of Santarém (−54.876133°; −2.334050°), State of Pará, Brazil.

Site of infection: Gills.

Prevalence: 3/4 (75%), 2/4 (75%), 1/2 (50%), mean intensity = 6, 9 and 5, and mean abundance of infectio*n* = 6, 2.5, and 5, from Igarapé Jari, Tapajós River Basin, municipality of Santarém; Tapajós River, National Park of Amazonia, municipality of Itaituba; and Tapajós River, Pimental, municipality of Itaituba, State of Pará, Brazil, respectively.

Deposited material: Holotype ZUEC PLA (222), paratypes 2 ZUEC PLA (225, 228), 2 MZUSP (8089a–b), 1 CHIOC (40492), 1 CHIBB (882L), voucher 4 ZUEC PLA (223–227), 4 MZUSP (8086, 8087a–b, 8088), 4 CHIOC (40493, 40494a–b, 40495), 3 CHIBB (883L–885L).

Etymology: the name of the species is a Latinized compound noun referring to the sac (from Greek σακούλα, sakoúla = bag) of the MCO (from the Latin cirrus).

#### Description

Description (based on 17 specimens: 9 stained on Gomori’s trichrome and mounted in Dammar gum, and 8 mounted on Gray and Wess’s medium). Body 304 (230–374; *n* = 17) long, bell-shaped; body 105 (65–176; *n* = 15) greatest width, at level of medium body. Cephalic region distinct from body, with poorly developed cephalic lobes; cephalic glands unicellular posterolateral to pharynx, opening in six pairs of head organs. Eyes and accessory chromatic granules absent. Pharynx subspherical 31 (17–49; *n* = 13) in diameter, muscular, glandular; esophagus short; two intestinal ceca, confluent posteriorly to gonads, lacking diverticula. Peduncle absent; haptor 43 (23–60; *n* = 17) long, 93 (53–134; *n* = 16) wide, subhexagonal, with four haptoral patches (one pair ventral and one pair dorsal). Ventral anchor 30 (25–38; *n* = 16) long, 16 (11–18; *n* = 14) base; dorsal anchor 35 (24–49; *n* = 16) long, 16 (12–21; *n* = 14) base; both with well-defined roots. Ventral anchor with superficial root more developed than deep root; small indentation at base precedes superficial root, which has thick sclerotization resembling a cap with short shaft, slightly curved, and with a straight and elongated point. Dorsal anchor with superficial root more developed than deep root; with thin sclerotization in the superficial root, short shaft, slightly curved and with a straight and elongated point. Ventral bar 56 (39 – 81; *n* = 16) long, distance between ends 46 (20–74; *n* = 16), slightly straight or as an expanded “U”, resembling an arc, with an anteromedial projection, and dilated and rounded ends; dorsal bar 59 (44–86; *n* = 17) long, distance between ends 48 (23–75; *n* = 16), slightly curved, V-shaped, with anteromedial concavity, and slightly tapered ends. Hooks similar in shape and length, robust, relatively long, with a straight tip, straight shaft, erect thumb, with a proximal dilation, followed by a tapering shank that distally expands and then tapers again; pair 1, 17 (13–22; *n* = 9); pair 2 and 7, 19 (12–28; *n* = 18); pairs 3, 4 and 6, 21 (12–26; *n* = 30); pair 5, 15 (10–19; *n* = 9) long. Common genital pore opening midventral near level of cecal bifurcation. MCO 77 (45–120; *n* = 16) long, coiled, with 4–5 counterclockwise rings 7.5 (6–12; *n* = 17) proximal ring diameter, resembling a corkscrew, and a semicircular expanded base. Sac of MCO present. Accessory piece 6 (3–11; *n* = 15) long, reduced, articulated with the base of the MCO by copulatory ligament. One prostatic reservoir. Intercecal and overlapped gonads. Testis 96 (52–162; *n* = 8) long, 34 (22–56; *n* = 6) wide, dorsal to germarium, oval; vas deferens arising from the mid-anterior region of testicle, dorsoventrally involving left branch of intestinal cecum, running to right side of trunk, twisting ventrally to anterior region, dilating to form seminal vesicle and twisting ventrally, to posteriorly connect to base of MCO. Vagina single, formed by a non-sclerotized and cup-shaped atrium, opening at left body margin; vaginal canal, a slightly straight and short tube, arranged in an angle of approximately 45 degrees to body. Uterus present; ootype at base of uterus; seminal receptacle not observed. Germanium 95 (69–117; *n* = 10) long, 34 (26–46; *n* = 8) wide, elliptical. Vitellaria well developed, coextensive with ceca, and absent in regions of reproductive systems.

#### Remarks

*Ameloblastella sakulocirra* n. sp. distinguishes from all other known species in the genus by the presence of a sac enveloping the MCO, a characteristic absent in its congeners. However, morphologically, this species shows a closer affinity to *A. martinae* and an undescribed *Ameloblastella* species recovered from *P. corruscans* and *P. reticulatum* from Argentina [67]. These three species stand apart from other *Ameloblastella* species due to their distinctive MCO morphology, characterized by a corkscrew-like shape, while the remaining species in the genus exhibit a coiled MCO composed of delicate rings.

The MCO of *A. sakulocirra* n. sp. consists of 4–5 tightly wound rings, similar to that of *A. martinae*, which has 4.5 tightly wound rings. In contrast, *Ameloblastella* sp. possesses 3 tightly wound rings and 1 loosely wound ring [67]. The accessory piece of *A. sakulocirra* n. sp. also resembles the accessory piece of *A. martinae*, being smaller in size and located distally at the MCO, also differing from the larger, medially located accessory piece of *Ameloblastella* sp. (Figs. 8 and 9, Table 3).

**Table 3:** Morphometric variables of specimens of *Ameloblastella* spp. closely related under morphological overview.

	*A. martinae*	*Ameloblastella* sp.	*A. sakulocirra* n. sp.
	Mendoza-Palmero *et al.* (2020) [67]	Mendoza-Palmero *et al.* (2020) [67]	Present study
BL	388 (295–445; *n* = 10)	–	304 (230–374; *n* = 17)
BGW	83 (70–100; *n* = 10)	–	105 (65–176; *n* = 15)
PD	26 (23–30; *n* = 10)	–	31 (17–49; *n* = 13)
HL	51 (43–79; *n* = 9)	–	43 (23–60; *n* = 17)
HW	120 (105–140; *n* = 9)	–	93 (53–134; *n* = 16)
VAL	30 (28–34; *n* = 11)	29 (28–29; *n* = 4)	30 (25–38; *n* = 16)
VAW	17 (14–19; *n* = 11)	16 (14–17; *n* = 4)	16 (11–18; *n* = 14)
DAL	37 (31–40; *n* = 11)	36 (35–36; *n* = 4)	35 (24, –49; *n* = 14)
DAW	17 (14–20; *n* = 11)	17 (14–19; *n* = 4)	16 (12–21; *n* = 14)
VBL	57 (49–65; *n* = 11)	54 (52–55; *n* = 4)	56 (39–81; *n* = 16)
DBL	56 (47–64; *n* = 11)	52 (50–54; *n* = 4)	59 (44–86; *n* = 17)
H15	18 (16–20; *n* = 11)	19 (17–19; *n* = 4)	16 (10–22; *n* = 18)
H23467	24 (23–26; *n* = 11)	24 (23–24; *n* = 4)	20 (12–28; *n* = 48)
MCOS	37 (32–42; *n* = 11)	35 (33–36; *n* = 4)	35 (21–80; *n* = 16)
MCOPR	8 (7–10; *n* = 11)	8 (8–9); *n* = 4)	7.5 (6–12; *n* = 17)
APL	9–15 (12; *n* = 8)	–	6 (3–11; *n* = 15)
GL	96 (69–130; *n* = 10)	–	95 (69–117; *n* = 10)
GW	29 (24–43; *n* = 10)	–	34 (26–46; *n* = 8)
TL	116 (100–140; *n* = 4)	–	96 (52–162; *n* = 8)
TW	29 (19–40 (*n* = 7)	–	34 (22–56; *n* = 6)
Locality	Peru, Iquitos	Argentina, Santa	Brazil, Itaituba and Santarém
		Fe Province	
Host	*S. lima, H. platyrhynchos, P. corruscans,* and *P. reticulatum*	*P. corruscans* and *P. reticulatum*	*P. hemioliopterus*

Abbreviations: body length (BL), body greatest width (BGW), pharynx diameter (PD), haptor long (HL), haptor wide (HW), ventral anchor length (VAL), ventral anchor base width (VAW), dorsal anchor length (DAL), dorsal anchor base width (DAW), ventral bar length (VBL), dorsal bar length (DBL), mean of marginal hooks 1 and 5 (H15), mean of marginal hooks 2–4, 6 and 7 (H23467), male copulatory organ space occupied in body (MCOS), male copulatory organ proximal ring diameter (MCOPR), accessory piece length (APL), germarium length (GL), germarium width (GW), testis length (TL), testis width (TW).

We have also identified some haptoral characteristics that distinguish *A. sakulocirra* n. sp. from *A. martinae* and *Ameloblastella* sp. In *A. sakulocirra* n. sp., the ventral anchor exhibits a small indentation at the base, just before the superficial root, followed by a sclerotized structure resembling a cap (Figs. 8 and 9). This feature is absent in *A. martinae* and *Ameloblastella* sp. [67]. Additionally, the deep root of the dorsal anchor is more pronounced in *A. sakulocirra* n. sp. compared to *A. martinae* and *Ameloblastella* sp. Furthermore, the hooks of *A. sakulocirra* n. sp. display a constriction in the proximal portion, which is not observed in *A. martinae* and *Ameloblastella* sp.

In conclusion, the morphometric analyses yielded strong support for the distinction of the three species (Fig. 10). The first linear discriminant accounted for approximately 84% of the observed separation (Table 4). Key characteristics that contributed significantly to the accurate classification included the length of the dorsal anchor (−3.23), the mean length of marginal hooks 2–4, 6, and 7 (−3.13), and the length of the dorsal bar (2.90). Our model achieved a high classification accuracy of 93% for the observed data. Further evaluation of the model against the training and test sets demonstrated its excellent fit and performance, with accuracy rates ranging from 93% to 100% (Table 5).

**Table 4:** Coefficients of linear discriminants of *Ameloblastella martinae*, *Ameloblastella sakulocirra* n. sp., and *Ameloblastella* sp.

	LD1	LD2
Proportion of trace	0.84	0.16
VAL	2.87	0.30
VAW	−1.37	−0.67
DAL	−3.23	−1.33
DAW	0.80	−0.07
VBL	2.34	0.05
DBL	−0.85	2.90
MCOL	−3.00	−0.81
MCOD	1.10	−0.64
H15	2.34	−1.57
H2467	−3.13	2.40

Abbreviations: VAL, ventral anchor length; VAW, ventral anchor base width; DAL, dorsal anchor length; DAW, dorsal anchor base width; VBL, ventral bar length; DBL, dorsal bar length; MCOL, largest space occupied by the MCO in body; MCORD, MCO 1st ring diameter; H15, mean of marginal hooks 1 and 5; H23467, mean of marginal hooks 2–4, 6 and 7.

**Table 5:** Prediction accuracy of the LDA model of *Ameloblastella martinae* (Peru), *Ameloblastella sakulocirra* n. sp. (Brazil), and *Ameloblastella* sp. (Argentina).

	Against training set			Against test set		
	Peru	Argentina	Brazil	Peru	Argentina	Brazil
Peru	4	0	0	6	0	0
Argentina	0	3	0	0	1	0
Brazil	0	0	10	1	0	7

### *Rhabdolachosus* n. gen.

urn:lsid:zoobank.org:act:73641E03-6A2A-47D2-AC5E-A9F77ED38717

Syn. *Demidospermus*: part Cepeda and Luque [16], 869–873, figs. 1–22.

Type species: *Rhabdolachosus brachyplatystomae* comb. n. (syn. *Demidospermus brachyplatystomae*) (Cepeda & Luque, 2010) [16], *ex Brachyplatystoma filamentosum* (Pimelodidae), from Araguaia River, State of Mato Grosso, Brazil.

Other species: *Rhabdolachosus araguaiaensis* comb. n. (syn. *Demidospermus araguaiaensis*) (Cepeda & Luque, 2010), *Rhabdolachosus ceccarellii* comb. n. (syn. *Demidospermus ceccarellii*) (Cepeda & Luque, 2010), *ex B. filamentosum*, from Araguaia River, State of Mato Grosso, Brazil [16].

Etymology: the name of the genus is a latinized compound noun from Greek and refers to the haptoral bars (ράβδοι, rávdoi) with grooves (αυλακώσεις, avlakóseis).

#### Diagnosis

Tegument annulations absent; eyes absent or one pair present; accessory chromatic granules absent; MCO not coiled, forming incomplete rings, C, G, J or U-shaped; accessory piece sheath-like, sometimes spiraled, not articulated or directly articulated with proximal region of MCO or with MCO base. Vaginal aperture sinistral, between MCO and germarium; vagina formed by atrium and pre-atrium or just atrium, muscular or sclerotized; vaginal canal sclerotized or not, curved or sinuous, straight or just curved in extremities. Gonads in tandem, intercecal, testis posterior to germarium. Peduncle medium to long-sized (exceeding 8% of total body length). One ventral and one dorsal haptoral bar, not articulated; ventral bar slightly straight; dorsal bar slightly straight or V, U, and W-shaped; longitudinal groove present, continuously or at ends of dorsal bar. Haptoral hooks approximately same size and shape or with some modification at pair 5. Parasites of pimelodids.

#### Remarks

The newly established genus *Rhabdolachosus* n. gen. is defined by the following characteristics: 1) a peduncle exceeding 8% of the total body length; 2) ventral bar slightly straight; 3) presence of grooves, either continuous or not, in the ventral bar; and 4) continuous grooves in the dorsal bar. The synapomorphy 1 and 2 are enough to distinguish the genus from almost all other *Demidospermus*-like species examined here, except for *A. unapi* and *D. mortenthaleri i. s*. Nonetheless, *Rhabdolachosus* species have gonads in tandem (overlapped in *A. unapi*), vaginal aperture between the MCO and the germarium level (next to the germarium level in *A. unapi*) and bears grooves in the vaginal atrium (absent in *A. unapi* and *D. mortenthaleri i. s*.). Additionally, *Rhabdolachosus* species do not have a medio-anterior or medio-posterior projection in the ventral bar (present in *A. unapi*), exhibit a short and cup-shaped vaginal atrium with grooves (in contrast to the large, urn-shaped, non-grooved vaginal atrium of *D. mortenthaleri i. s*.), along with a contiguous distal part of the vaginal canal (unlike the bifurcated distal part of the vaginal canal in *D. mortenthaleri i. s*.) (Fig. 20, Table S2).

In comparison to the diagnosis outlined by Cepeda and Luque (2010) [16], our study introduces distinct morphological interpretations. Specifically, the positioning of the haptoral complex in *R. brachyplatystomae* comb. n. and *R. ceccarellii* comb. n. (Figs. 11–12) was inverted in the original description. Thereby, the ventral bars generally exhibit slight straightness across the three species, with the possibility of being slightly bowed in *R. brachyplatystomae* comb. n. The dorsal bar in these species can present either a curved or slightly straight configuration (Table S2). Similar to the observations made by Cepeda and Luque (2010) [16], we attribute the polymorphisms in the haptoral complex and MCO morphology of *R. brachyplatystomae* comb. n. to intraspecific variations (Fig. 12). Variations in hook and accessory piece morphology were also noted in *R. ceccarellii* comb. n. This species displays alterations in hook pair 5, characterized by increased length and reduced thickness, accompanied by a vestigial or underdeveloped thumb. Other hooks of this species demonstrate noticeable expansion in the proximal region. Notably, its accessory piece is proportionally longer than initially described and exhibits an expanded distal portion that guides the MCO (Fig. 11).

The copulatory organ of *R. araguaiaensis* comb. n. exhibits distinctive features within the *Demidospermus*-like species. It possesses a bifurcated base, with one part directed laterally and the other posteriorly (Fig. 13). The laterally directed base accommodates the proximal portion of the accessory piece, to which it articulates. From the posteriorly directed part, extensions of prostatic glands emerge, initially appearing glandular and later filamentous (Fig. 13). Other characteristics were also observed in this species, including the junction between the vaginal canal and the seminal receptacle, as well as the ootype and Mehlis’ glands (Fig. 13).

Cruces *et al.* (2024) [22] proposed *Peruanella* Cruces, Santillán, Silvera, Morey, Rubin & Chero, 2024, to accommodate *Peruanella madredediosensis* Cruces, Santillán, Silvera, Morey, Rubin & Chero, 2024, parasite of *Brachyplatystoma tigrinum* (Britski, 1981) (Pimelodidae), and *Peruanella aureagarciae* (Morey, Rojas, Dávila, Chu & De Pina, 2024), parasites of *Pseudoplatystoma punctifer* (Castelnau, 1855) (Pimelodidae), from Belén Market, Loreto, Peru [75]. This genus shares with *Rhabdolachosus* n. gen. the absence of eyes, a non-coiled MCO (*i.e.*, a curved tube, forming incomplete rings, C, G, J, or U-shaped), a peduncle exceeding 8% of the body total length, and a slightly straight ventral bar. However, *Rhabdolachosus* n. gen. differs from *Peruanella* by having a left vaginal aperture (midventral aperture in *Peruanella*). Nevertheless, further investigation will be needed to test the phylogenetic relationship between these genera.

### *Aphanoblastella* Kritsky, Mendoza-Franco & Scholz, 2000

Syn. *Cleidodiscus*: part Price [81], 407–408, pl. I, figs. 1–3; *Urocleidoides*: part Mizelle and Kritsky [72], 384, figs. 65–73, part Suriano [97], 74–78, figs. 1–17.

Type species: *Aphanoblastella travassosi* (Price, 1938), *ex Rhamdia laticauda* (Kner, 1858) from San Pedro Montes de Oca, Costa Rica.

Other species: *Aphanoblastella mastigatus* (Suriano, 1986), *ex Rhamdia quelen* (Quoy & Gaimard, 1824), Argentina [97]; *Aphanoblastella robusta* (Mizelle & Kritsky, 1969), *ex Rhamdia* sp. Amazonas River Basin, Brazil [81]; *Aphanoblastella chagresii* Mendoza-Franco, Aguirre-Macedo, Vidal-Martínez, 2007, *ex Pimelodella chagresi* (Steindachner, 1877), Rio Frijolito, Republic of Panama [62]; *Aphanoblastella juizforense* Carvalho, Tavares & Luque 2009, *ex R. quelen*, Paraibuna River, Juiz de Fora, State of Minas Gerais, Brazil [15]; *Aphanoblastella aurorae* Mendoza-Palmero, Scholz, Mendoza-Franco & Kuchta, 2012, *ex Goeldiella eques* (Müller & Troschel, 1849), Iquitos, Peru [68]; and *Aphanoblastella magna* Yamada, Acosta, Yamada, Scholz & Silva, 2018 *ex Pimelodella avanhandavae* Eigenmann, 1917, Aguapeí River, Upper Paraná River, State of São Paulo, Brazil [103].

#### Emended diagnosis

Tegument annulations absent; two pairs of eyes, posterior larger and closer than anterior; accessory chromatic granules usually present. MCO corkscrew-like coil, sigmoid or sinuous; accessory piece sheath-like or rod-shaped, sometimes directly articulated with proximal region of MCO or with MCO base. Vaginal aperture sinistral, between MCO and germarium; vagina formed by atrium or pre-atrium, muscular or sclerotized; vaginal canal sclerotized, curved, or sinuous. Gonads in tandem, intercecal, testis posterior to germarium. Peduncle long-sized (exceeding 16% of the total body length). One ventral and one dorsal haptoral bar, not articulated, or just dorsal articulated; ventral and dorsal bars V, U, or W-shaped; ventral bar with a medio-anterior or medio-posterior projection. Haptoral hooks approximately the same size and shape. Parasites of heptapterids.

#### Remarks

The taxonomic history of *Aphanoblastella* began with the description of *Cleidodiscus travassosi* Price, 1938, the type species of the genus [81]. This species was found inhabiting the gills of *Rhamdia rogersi* (Regan, 1907) – now regarded as a junior synonym of *Rhamdia laticauda* (Kner, 1858) – from San Pedro de Montes de Oca, San José Province, Costa Rica. Molnar *et al.* (1974) [73] subsequently reclassified this species as *Urocleidoides travassosi* (Price, 1938) after examining specimens found parasitizing *Rhamdia quelen* (Quoy & Gaimard, 1824) from the Cumuto River near Coryal, Trinidad and Tobago. During this revision, they presented an alternative interpretation of the morphological features, depicting the MCO with delicate rings, thus divergent from the initial screw-like MCO description. The authors also highlighted an accessory piece articulated at the base of the MCO, along with disparities in the morphology of the bars. Importantly, they deposited three specimens labeled USNPC 73179 (USNM 1368746), which furnished the material for our examinations of these variations.

Later, Suriano (1986) [97], in congruence with the proposed reclassification by Molnar *et al.* (1974) [73], reported *U. travassosi* associated with *Pimelodella laticeps* Eigenmann, 1917, another heptapterid from Laguna de Chascomus, in the province of Buenos Aires, Argentina. Suriano (1986) [97] also exhibited a distinct interpretation of the MCO, depicting a loosely sinuous MCO with a ring, and an unarticulated accessory piece at the base of the MCO. However, the interpretation of the bars closely aligned with those offered by Molnar *et al.* (1974) [73], despite the authors omitting to deposit any specimens in scientific collections. Afterward, Kritsky *et al.* (1986) [51] categorized this species as *incertae sedis* due to the absence of the vaginal sclerite, a synapomorphy of *Urocleidoides* Mizelle & Price, 1964. Ultimately, this species was transferred to *Aphanoblastella* as *Aphanoblastella travassosi* [50].

Kritsky *et al.* (2000) [50] provided a closer description of *A. travassosi*, compared to the original description, when reporting this species from the gills of *Rhamdia guatemalensis* (Günther, 1864) from Ixin-Há Cenote, Yucatan, Mexico. This characterization was also echoed by researchers such as Mendoza-Franco *et al.* (2007) [62], who reported *A. travassosi* in *R. quelen* from Lago Alajuela, in the Republic of Panama. Considering the wide geographic distribution and host range of *A. travassosi*, uncommon for other species of the genus [103], and the varying morphological traits observed in specimens collected from diverse hosts and localities, it is likely that multiple species are being mistaken for one. This challenges the hypothesis by Yamada *et al.* (2018) [103] proposing *A. travassosi* to be a widely distributed euryxenous species. This long-time taxonomic impasse can only be resolved by accurately examining specimens from the original type locality and host (*i.e.*, *R. laticauda* from San Pedro de Montes de Oca, San José Province, Costa Rica). However, the description provided by Kritsky *et al.* [50] (here referred to as morphotype B) fits with the original description, while that of Molnar *et al.* [73] (here designated as morphotype A), and the variation noted by Suriano (1986) [97], probably correspond to other species that must be formally described.

*Aphanoblastella* forms a monophyletic group, as demonstrated in Figure 20, which reinforces earlier conclusions drawn from molecular analyses [1, 61, 65, 66, 103]. The genus is supported by: 1) the presence of two pairs of equidistant eyes, with the posterior pair being larger than the anterior, only shared with *Sicohencotyle antomaia* n. gen. n. sp.; 2) the peduncle exceeding 16% of the total body length; and 3) the presence of a medio-anterior or medio-posterior projection in the ventral bar. While the medium-sized peduncle is widely distributed among other lineages, the presence of a projection in the ventral bar is just shared with *Ameloblastella*. Notably, both character states seem to have evolved independently in their respective lineages.

### *Omothecium* Kritsky, Thatcher & Boeger, 1987

Syn. *Demidospermus*: part Kritsky and Gutiérrez [48], 148–149*.*

Type species: *Omothecium pinirampi* (syn. *Demidospermus pinirampi*) Kritsky, Thatcher & Boeger, 1988, *ex Pinirampus pinirampu* (Spix & Agassiz, 1829) (Pimelodidae), from Janauacá, Lake, Solimões River, State of Amazonas, Brazil [52], *ex Pimelodina flavipinnis* Steindachner, 1876 (Pimelodidae), from Reservoir of Lajeado, middle Tocantins River, municipality of Porto Nacional, State of Tocantins, Brazil [4].

Other species: *Omothecium luckyi* (syn. *Demidospermus luckyi*) Kritsky, Thatcher & Boeger, 1988, *ex. P. pinirampu*, Janauacá Lake, State of Amazonas, Brazil [52] and *Omothecium macropteri* (syn. *Demidospermus macropteri*) (Mendoza-Franco & Scholz, 2009) comb. n., *ex Calophysus macropterus* (Pimelodidae), Iquitos, Peru [64].

#### Emended diagnosis

Tegument annulations absent; eyes variable, absent, or just anterior pair present, or two pairs present with posterior larger and closer than anterior, or posterior and/or anterior fused; accessory chromatic granules absent or present. MCO coiled forming counterclockwise rings, or a curved tube, forming incomplete rings as C, G, J, and U-shaped; base of the MCO globose, with two expansions like flaps; accessory piece sheath-like, not articulated. Vaginal aperture sinistral, next to MCO level; vagina formed by sclerotized atrium; vaginal canal sclerotized, sigmoid, or a slightly straight tube or curved in extremities. Gonads in tandem, intercecal, testis posterior to germarium. Peduncle medium-sized (from 8 to 15% of total body length). One ventral and one dorsal haptoral bar, not articulated, both V, U, or W-shaped or dorsal bar slightly straight; extremities of ventral bar tapering or contiguous; extremities of dorsal bar contiguous or expanded. Haptoral hooks equal or unequal in size and shape; when unequal, pairs 5 and 6 can be longer and/or with shank thinner than the others, with a depressed, flattened, or vestigial thumb, and pair 2 can have a significantly reduced shank. Parasites of pimelodids.

#### Remarks

*Omothecium* was originally established to encompass *O. luckyi* and *O. pinirampi*, characterized by: 1) a weakly sclerotized vagina, sinistral, opening close to the MCO; 2) gonads in tandem; 3) unmodified anchors and bars; 4) non-dilated hooks; and 5) an MCO coiled in clockwise rings [52]. Later, the genus was synonymized with *Demidospermus* by Kritsky and Gutiérrez (1998) [48], who transferred its species, renaming them as *Demidospermus pinirampi* (Kritsky, Thatcher & Boeger, 1987) and *Demidospermus luckyi* (Kritsky, Thatcher & Boeger, 1987). In our phylogenetic inference (Fig. 20), the clade corresponding to *Omothecium* was recovered, supporting our proposal to resurrect the genus, which is now characterized by: 1) a globular base of the MCO; 2) 2 flaps arising from the base of the MCO; 3) a sinistral vaginal opening next to the MCO level; and 4) absence of dilated hooks. The species currently included in the genus have a sclerotized vaginal atrium and an MCO like a curved tube, forming incomplete rings, something like a C, G, J, or U-shaped. Along with the species originally described in the genus, *O. pinirampi* and *O. luckyi*, we transfer *Demidospermus macropteri* Mendoza-Franco & Scholz, 2009, which is now designated as *Omothecium macropteri* (Mendoza-Franco & Scholz, 2009) comb. n. This set of synapomorphies is sufficient to distinguish these species from all other genera and species analyzed herein.

### *Martorellius* n. gen.

urn:lsid:zoobank.org:act:4B8E19BD-A06A-48EF-9AB9-01788B3915D8

Syn. *Demidospermus*: part Gutiérrez and Suriano [39], 170–171, figs. 1–14; part Kritsky and Gutiérrez [48], 148–150, figs. 2–3, 13–24, 35–46; part Monteiro, Kritsky and Brasil-Sato [74], 187; part Mendoza-Palmero and Scholz [69], 587–589, figs. 15, 21.

Type species: *Martorellius curvovaginatus* (syn. *Demidospermus curvovaginatus*) (Mendoza-Palmero & Scholz, 2011) comb. n., *ex Pimelodus* sp. (Pimelodidae) [69].

Other species: *Martorellius valenciennesi* (syn. *Demidospermus valenciennesi*) (Gutiérrez & Suriano, 1992) comb. n. (Fig. 15), *ex Parapimelodus valenciennis* (syn. *Parapimelodus valenciennesi*) (Lütken, 1874) [39, 48] and *Pimelodus maculatus* Lacépède, 1803, *Pimelodus* sp., from De La Plata River, Buenos Aires, Argentina [39, 48] and Reservoir of Itaipú Hydroelectric Power Station, Paraná River, State of Paraná, Brazil [21]; *Martorellius paravalenciennesi* (syn. *Demidospermus paravalenciennesi*) (Gutiérrez & Suriano, 1992) comb. n. (Fig. 14), *ex Pimelodus maculatus* (syn. *Pimelodus clarias*) Lacépède, 1803 (Pimelodidae), from Buenos Aires, Argentina [37–39, 48], Guandu River, State of Rio de Janeiro, Brazil [8, 87], Reservoir of Itaipú Hydroelectric Power Station, Paraná River, State of Paraná, Brazil [21], São Francisco River, Três Marias, State of Minas Gerais, Brazil [74], *ex Prochilodus lineatus* (Prochilodontidae) (Valenciennes, 1837), from the Batalha River, State of São Paulo, Brazil [55]; *Martorellius peruvianus* (syn. *Demidospermus peruvianus*) (Mendoza-Palmero & Scholz, 2011) comb. n., *ex Pimelodus ornatus* Kner, 1858 (Pimelodidae), and *Martorellius striatus* (syn. *Demidospermus striatus*) (Mendoza-Palmero & Scholz, 2011) comb. n., *ex Pimelodus* sp. (Pimelodidae) from Iquitos, Peru [69].

Etymology: the name is a Latinized noun, in honor of the researcher Sergio R. Martorelli for his contributions to Neotropical parasitology.

#### Diagnosis

Tegument annulations absent; two pairs of eyes, posterior larger and closer than anterior, or posterior and/or anterior fused; accessory chromatic granules absent or present. MCO a curved tube, forming incomplete rings, C, G, J, or U-shaped; MCO base globose or doubly globose; MCO base expansions absent or with one to two flaps; accessory piece sheath-like, not articulated. Vaginal aperture sinistral, at germarium level or between MCO and germarium; vagina formed by an atrium, or by pre-atrium and atrium, all sclerotized; vaginal canal sclerotized, sinuous, proximally dorsal and distally ventral. Gonads in tandem, intercecal, testis posterior to germarium. Peduncle short to medium-sized (from 1 to 15% compared to body total length); haptoral patches present or absent. One ventral and one dorsal haptoral bar, not articulated, both V, U, or W-shaped; extremities of ventral bar tapering; sclerotized narrowing in middle of dorsal bar present or absent. Haptoral hooks unequal in size and shape; pair 1 dilated, composed of one subunit; pair 2 with shank significantly reduced. Parasites of pimelodids.

#### Remarks

The genus proposed here is supported by unique synapomorphy: a sinuous vaginal canal that is dorsally proximal and ventrally distal (Fig. 20). Five species previously classified under *Demidospermus* have been reassigned to this genus. These species include *M. valenciennesi* comb. n., *M. paravalenciennesi* comb. n., *M. curvovaginatus* comb. n., *M. peruvianus* comb. n., and *M. striatus* comb. n., all parasites of siluriforms, although *M. paravalenciennesi* comb. n. has been reported in characiform fish [55]. Additionally, species of *Martorellius* n. gen. share other morphological features, including a dilated hook pair 1 composed of a single unity (also observed in *D. cornicinus i. s.* and *D. brevicirrus i. s.*); a reduced shank of hook pair 2 (trait also found in some species of *Omothecium* and *Paramphocleithrium*, *D. brevicirrus i. s.*, *D. cornicinus i. s.*, and *D. brevicirrus i. s.*); a slightly more robust hook pair 7 compared to pairs 2–6 (not dilated, as in *D. idolus i. s.*, *D. armostus i. s.*, and *Sicohencotyle lebedevi* comb. n.); and a non-coiled MCO, resembling various shapes such as a curved tube, incomplete ring, or C, G, J, or U-shaped structures, characteristics commonly found in other *Demidospermus*-like species. However, this set of characteristics is enough to distinguish this genus from others analyzed here.

Species of *Martorellius* n. gen., notably *M. valenciennesi* comb. n., *M. paravalenciennesi* comb. n. (Figs. 14 and 15), and *M. curvovaginatus* comb. n., exhibit remarkable morphological similarities. When Gutiérrez and Suriano (1992) [39] described *M. valenciennesi* comb. n. and *M. paravalenciennesi*, they differentiated these species based on: the number and shape of head organs (2 globular and 3 spindle-shaped in *M. valenciennesi* comb. n. *versus* 3 globular and 2 spindle-shaped in *M. paravalenciennesi* comb. n.); the shape of hooks (hook pairs 1 and 7 larger and more robust than pairs 2–6 in *M. valenciennesi* comb. n. *versus* hook pair 7 larger and more robust than pairs 1–6 in *M. paravalenciennesi* comb. n.); the size of the testis relative to the germarium (testis larger than germarium in *M. valenciennesi* comb. n. *versus* smaller than germarium in *M. paravalenciennesi* comb. n., without providing measurements of testes for both species); and the smaller MCO (50.5 μm *versus* 68.3 μm) and accessory piece (18 μm *versus* 27 μm) of *M. valenciennesi* comb. n. compared to *M. paravalenciennesi* comb. n. Later, Kritsky and Gutiérrez (1998) [48] redescribed these species from the same host and locality.

Regarding head organs, Kritsky and Gutiérrez (1998) [48] did not provide comparative information. However, concerning MCO measurements, they presented data that supported Gutiérrez and Suriano’s (1992) [39] findings. However, concerning hooks, these authors reported that hook pairs 1 and 7 are, as in *M. valenciennesi* comb. n., larger than the other pairs in *M. paravalenciennesi* comb. n., aligning with the interpretation of hooks in both species. Regarding the sizes of the germarium and testis, Kritsky and Gutiérrez (1998) [48] provided measurements showing the testis larger than the germarium for both species, indicating similarity between these species and contradicting previous data provided by Gutiérrez and Suriano (1992) [39]. Therefore, according to the study by Kritsky and Gutiérrez (1998) [48] and the material examined in the present study (Figs. 14, 15, 20), *M. valenciennesi* comb. n., and *M. paravalenciennesi* comb. n. are differentiated based on the measurements of the MCO and accessory piece, the morphology of the vagina, the number of head organs, and the absence of a prostatic reservoir in *M. valenciennesi* comb. n. (*versus* one prostatic reservoir in *M. paravalenciennesi* comb. n.).

In the description of *M. curvovaginatus* comb. n., Mendoza-Palmero and Scholz (2011) [69] considered this species morphologically similar to *M. paravalenciennesi* comb. n., from which it was differentiated based on the presence of a flap on the base of the MCO (absent in *M. paravalenciennesi* comb. n.). Additionally, the authors considered the MCO of *M. curvovaginatus* comb. n. less coiled than that of *M. paravalenciennesi* comb. n., with a narrower base. These authors also reported the vaginal atrium of *M. curvovaginatus* comb. n., sclerotized (*versus* weakly sclerotized in *M. paravalenciennesi*), and funnel-shaped with a posteriorly directed vaginal canal, whereas in *M. paravalenciennesi* comb. n., the shape of the vaginal atrium would be different from this shape and would not present a posteriorly directed vaginal canal. Examining the paratype (USNM 1399527) and vouchers (USNM 1399528 and USNM 1399529) of *M. curvovaginatus* comb. n., we observed that the presence of the flap at the base of the MCO and the presence of two pairs of eyes, one of them fused (only the posterior pair larger and closer and not fused in *M. paravalenciennesi* comb. n.), differentiate it from *M. paravalenciennesi* comb. n. The vaginal atrium of these two species appeared to us to have the same sclerotization consistency and similar morphology, cup-shaped and not funnel-shaped. While the morphology of the vaginal canal is shared by all species of the genus. Despite the observed similarity between *M. valenciennesi* comb. n., *M. paravalenciennesi* comb. n., and *M. curvovaginatus* comb. n., these three species show specific variations that allow them to be distinguished. However, these morphological boundaries lack integrative support, which should be addressed in future studies.

### *Paramphocleithrium* Suriano & Incorvaia, 1995

Syn. *Demidospermus*: part Gutiérrez and Suriano [39], 171–172, figs. 23–29; part Kritsky and Gutiérrez [48], 152–154, figs. 47–56.

Type species: *Paramphocleithrium bidiverticulatum* (syn. *Demidospermus bidiverticulatum*) Suriano & Incorvaia, 1995, *ex Pimelodus maculatus* (syn. *Pimelodus clarias maculatu*s) Lacépède, 1803 (Pimelodidae), from de La Plata River, Buenos Aires, Argentina [95], Reservoir of Itaipú Hydroelectric Power Station, Paraná River, State of Paraná [21], and São Francisco River, near the municipality of Três Marias, Brazil [74], Brazil; *ex Pimelodus albicans* (Valenciennes, 1840) (Pimelodidae), from de La Plata River, Buenos Aires, and Salado del Norte River, San Justo, Argentina [17, 48]; *ex Pimelodus* sp. (Pimelodidae), from Reservoir of Itaipú Hydroelectric Power Station, Paraná River, State of Paraná [21]; *ex Auchenipterus osteomystax* (Miranda Ribeiro, 1918) (Auchenipteridae), from Reservoir of Itaipú Hydroelectric Power Station, Paraná River, State of Paraná [21].

Other species: *Paramphocleithrium ichthyocercus* (syn. *Demidospermus ichthyocercus*) comb. n. (Monteiro, Kritsky & Brasil-Sato, 2010), *ex P. maculatus* (Pimelodidae), from São Francisco River, near the municipality of Três Marias, State of Minas Gerais, Brazil [74].

#### Emended diagnosis

Tegument annulations absent; two pairs of eyes, posterior larger and closer than anterior, or posterior and/or anterior fused; accessory chromatic granules absent. MCO a curved tube, forming incomplete rings, C, G, J, or U-shaped; base of MCO rounded, simple, just differentiated from MCO or globose; accessory piece composed of two sclerotized pieces, proximally or distally articulated, or a piece sheath-like, all directly articulated with proximal region of MCO or with MCO base. Vaginal aperture sinistral, next to MCO or between MCO and germarium; vagina composed of a large and sclerotized atrium, urn-shaped, with or without fringes; vaginal canal sclerotized, a curved, or sinuous tube, or a straight tube curved in extremities. Gonads in tandem, intercecal, testis posterior to germarium. Peduncle medium; haptoral patches present. One ventral and one dorsal haptoral bar, both not articulated, V, U, or W-shaped; dorsal bar with or without expanded ends. Haptoral hooks unequal in size and shape; pair 2 with or without a significantly reduced shank; pair 5 and 6 longer and/or with a shank thinner than the others, with a depressed, flattened, or vestigial thumb and a sub-developed or undeveloped point. Parasites of pimelodids and auchenipterids.

#### Remarks

*Paramphocleithrium* was proposed by Suriano and Incorvaia (1995) [95] to accommodate *P. bidiverticulatum*, which, according to the authors, lacks spermatozoa packages, possesses two prostatic reservoirs, and exhibits distinct morphological features of haptoral structures. The genus was named after the presence of two intestinal diverticula, according to the same authors. Subsequently, Kritsky and Gutiérrez (1998) [48] synonymized *Paramphocleithrium* with *Demidospermus*, based on studies conducted on specimens collected from the type locality and type host, adding a new record for hosts. In this study, the authors presented drawings that only show a prostatic reservoir and no intestinal diverticula, which we corroborate by analyzing the specimens in the present study (USNM 1382350 and USNM 1382351).

In our phylogenetic inferences, this genus was recovered (Fig. 20), supporting its resurrection. The main synapomorphy of the genus is the presence of an urn-shaped vaginal atrium. Although this feature is shared with *D. mortenthaleri i. s*., species of *Paramphocleithrium* differ by having an accessory piece directly articulated with the proximal region of the MCO (not articulated in *D. mortenthaleri i. s*.), by having a vagina composed of one atrium (one atrium and one pre-atrium in *D. mortenthaleri i. s*.) and by not presenting grooves on the ventral bar (grooves present on the ventral bar of *D. mortenthaleri i. s*.).

### *Magnanchistrius* n. gen.

urn:lsid:zoobank.org:act:A55805B6-30FC-4E7D-B72F-FD221F7DA98B

Syn. *Demidospermus*: part Gutiérrez and Suriano [39], 171–172, figs. 23–29; part Kritsky and Gutiérrez [48], 156–157, figs. 88–97, 78–87.

Type species: *Magnanchistrius uncusvalidus* (syn. *Demidospermus uncusvalidus*) comb. n. (Gutiérrez & Suriano, 1992), *ex Pimelodus maculatus* (syn. *Pimelodus clarias*) Lacépède, 1803 (Pimelodidae), from de La Plata River, Buenos Aires, Argentina [39], Guandu River, State of Rio de Janeiro [8, 87], Reservoir of Itaipú Hydroelectric Power Station, Paraná River [21], and São Francisco River, Três Marias, State of Minas Gerais [74], Brazil; *ex Pimelodus* sp. (Pimelodidae), from Reservoir of Itaipú Hydroelectric Power Station, Paraná River [21]; and *Trachelyopterus galeatus* (syn. *Parauchenipterus galeatus*) (Linnaeus, 1766) (Auchenipteridae), from de La Plata River, Buenos Aires, Argentina [39].

Other species: *Magnanchistrius majusculus* (syn. *Demidospermus majusculus*) comb. n. (Kritsky & Gutierrez, 1998), *ex Pimelodus albicans* (Valenciennes, 1840) (Pimelodidae) from de La Plata River, Buenos Aires, and Salado del Norte River San Justo, Argentina [17, 39, 40], *ex Pimelodus maculatus* (Pimelodidae) from Guandu River, State of Rio de Janeiro, Brazil [8, 87]; *Magnanchistrius leptosynophallus* (syn. *Demidospermus leptosynophallus*) comb. n. (Kritsky & Gutierrez, 1998), *ex Bergiaria westermanni* (syn. *Iheringichthys westermanni*) (Lütken, 1874) (Pimelodidae), from de La Plata River, Buenos Aires, Argentina [48], *ex Iheringichthys labrosus* (Pimelodidae) from Lagoa dos Patos, Paraná River, and from Reservoir of Itaipú Hydroelectric Power Station, Paraná River, State of Paraná, Brazil [21, 25]; *ex P. maculatus*, from Guandu River, State of Rio de Janeiro [8]; *ex Pimelodus blochii* Valenciennes, 1840 (Pimelodidae), from Iaco River, Sena Madureira, and Acre River, Rio Branco, State of Acre, Brazil [76]; *ex Pimelodella* sp. (Heptapteridae) and *Pimelodus* sp. (Pimelodidae), from Reservoir of Itaipú Hydroelectric Power Station, Paraná River, Brazil [21].

Etymology: the name of the genus is a Latinized compound noun and refers to the dilated (from Latin *magna* = large) hooks (from Greek *αγκίστρια*, ankístria *=* hooks) pairs 1, 2, and 7.

#### Diagnosis

Tegument annulations absent; two pairs of eyes, posterior larger and closer than anterior, or posterior and/or anterior fused; accessory chromatic granules absent or present. MCO a curved tube, forming incomplete rings, C, G, J, or U-shaped; base of MCO rounded, simple, just differentiated from MCO; distal part of MCO contiguous or contains extensions resembling barbs; accessory piece sclerotized, sheath-like, not articulated. Vaginal aperture sinistral, between MCO and germarium; vagina composed of sclerotized atrium and muscular pre-atrium; vaginal canal sclerotized or weekly sclerotized, a slightly straight tube or curved in extremities. Gonads in tandem, intercecal, testis posterior to germarium. Peduncle short to medium-sized (from 1 to 15% compared to total body length); haptoral patches absent. One ventral and one dorsal haptoral bar, both V, U, or W-shaped, only the ventral articulated; ventral bar with a sclerotized narrowing in the middle; dorsal bar with or without a medio anterior/posterior projection. Haptoral hooks unequal in size and shape; pairs 1, 2, and 7, dilated, composed of two subunits; pairs 5 and 6 longer and/or with a shank thinner than the others, with a depressed, flattened, or vestigial thumb and a sub-developed or undeveloped point. Parasites of auchenipterids, heptapterids, and pimelodids.

#### Remarks

*Magnanchistrius* n. gen. is supported by the following synapomorphies: 1) two flaps at the base of the MCO; 2) two haptoral bars, with the ventral one articulated; 3) ventral bar V, U, or W-shaped; 4) hooks of pairs 1, 2, and 7, dilated and composed of two units; and 5) recurved tips of the ventral and dorsal anchors. It is, in our phylogenetic inference (Fig. 20), the best-supported genus, by the number of synapomorphies and Bremer support, since *Demidospermus* is the genus best supported by the number of exclusive synapomorphies.

Among the *Demidospermus*-like species, the V, U, or W-shaped ventral bar is a widely distributed characteristic. However, the ventral bar in this format and still articulated is only shared with *Demidospermus stricto sensu.* Nevertheless, only *Magnanchistrius* n. gen. uniquely presents the articulated ventral bar, once in *Demidospermus stricto sensu*, both ventral and dorsal bars are articulated. The dilated hooks of pairs 1, 2, and 7, composed of two subunits, are also an exclusive characteristic of *Magnanchistrius* n. gen. The remaining characteristics constitute homoplasies, such as the two flaps protruding from the base of the MCO, which are also shared by species of *Omothecium*, and the recurved tips of the ventral and dorsal anchors, also shared with *A. travassosi* (morphotype A). However, this set of characteristics is enough to distinguish this genus from the others analyzed here.

### *Sicohencotyle* n. gen.

urn:lsid:zoobank.org:act:91B3B246-AD7D-4582-B10C-62FF56A0B89D

Syn. *Urocleidoides*: part 380–382, figs. 37–45, 46–55 [72].

Type species: *Sicohencotyle antoniomaiai* n. gen., n. sp., *ex Phractocephalus hemioliopterus* (Bloch & Schneider, 1801) (Pimelodidae), from Igarapé Jari, Tapajós River Basin, municipality of Santarém, State of Pará, Brazil.

Other species: *Sicohencotyle lebedevi* (syn. *Urocleidoides lebedevi*) (Kritsky & Thatcher, 1976) comb. n., *ex Pimelodus grosskopfii* (Pimelodidae) [53], Cali, Colombia and *Sicohencotyle catus* (syn. *Urocleidoides catus*) (Mizelle & Price, 1964) comb. n., *ex P. hemioliopterus*, State of Amazonas, Brazil [72].

Etymology: The name of the genus is in honor of the researcher Simone Chinicz Cohen for her contributions to the study of the biodiversity of fish parasites from South America, plus *cotyle* (from Greek *κοτύλη* that means cup or bowl and refers to the haptor of Monopisthocotyla).

#### Diagnosis

Tegument annulations absent; two pairs of eyes, posterior larger and closer or equidistant compared to anterior, or posterior and/or anterior fused; accessory chromatic granules absent or present. MCO coiled forming counterclockwise rings; distal part of MCO expanded or containing extensions resembling barbs; base of MCO expanded or rounded, simple, just differentiated from MCO; accessory piece sclerotized, sheath-like, not articulated. Vaginal aperture sinistral, between MCO and germarium; vagina composed of atrium and pre-atrium or simply pre-atrium; vaginal atrium and pre-atrium, when present, sclerotized or muscular; pre-atrium with or without grooves; atrium with or without a claw-shaped internal structure; vaginal canal not sclerotized; absence of seminal receptacle. Gonads in tandem, intercecal, testis posterior to germarium. Peduncle medium to long. Haptoral patches absent. One ventral and one dorsal haptoral bar; ventral bar slightly straight; dorsal bar slightly straight or V, U, and W-shaped. Haptoral hooks unequal in size and shape; pairs 5 and 6 longer and/or with a shank thinner than the others, with a depressed, flattened, or vestigial thumb and with or without a sub-developed or undeveloped point; pair 2 with a normal or a significantly reduced shank. Parasites de pimelodids.

#### Remarks

The genus *Sicohencotyle* n. gen. is characterized by the following synapomorphies: 1) the presence of 2–3 counterclockwise rings before the accessory piece, and 2) the presence of grooves in the vaginal atrium. While the first characteristic is shared with *Ameloblastella chavarriai*, *A. paranaensis*, *Aphanoblastella travassosi* morphotype B, and *D. spirophallus*, the second is shared with species of *Rhabdolachosus* n. gen., *Omothecium*, as well as *V. janauacaensis*, *A. chavarriai*, *D. centromochli s. m*., *D. annulus i. s*., *M. striatus* comb. n., *D. idolus i. s*., *D. cornicinus i. s*., and *D. armostus i. s*. However, the combination resulting from the presence of these two characteristics is only shared with *A. chavarriai*. Nevertheless, species of *Sicohencotyle* n. gen. differ from *A. chavarriai* in several aspects. Species of *Sicohencotyle* n. gen. possess an accessory piece formed by a single sclerotized piece, sheath-like (two sclerotized pieces, jointed proximally or distally in *A. chavarriai*), and not articulated with the proximal region of the MCO or with the MCO base (articulated by a ligament in *A. chavarriai*). *Sicohencotyle* n. gen. is also characterized by having gonads in tandem (overlapped in *A. chavarriai*), and a distal part of the vaginal canal contiguous (bifurcated in *A. chavarriai*). Species of *Sicohencotyle* n. gen. lack glandular haptoral patches (present in *A. chavarriai*), do not have a medio anterior or medio posterior projection in the ventral bar (projection present in *A. chavarriai*), exhibit a longitudinal groove in the ventral and dorsal bars (longitudinal groove absent in the bars of *A. chavarriai*), and show modifications in the shanks and thumbs of hook pairs 5 and 6 (modifications absent in *A. chavarriai*). *Boegeriella* Mendoza-Palmero & Hsiao, 2020, a genus of dactylogyrids parasites of Amazonian catfishes [66], also resembles *Sicohencotyle* n. gen., mainly considering the morphology of the MCO and haptoral structures. However, the accessory piece of *Boegeriella* is articulated with the base of the MCO by a ligament, whereas the accessory piece of *Sicohencotyle* n. gen. species is not articulated. *Boegeriella* is also characterized by having a folded germarium, while *Sicohencotyle* n. gen. has a simple germarium. Additionally, the vagina of *Sicohencotyle* n. gen. species exhibits grooves, which are not present in *Boegeriella* species*.*

Until now, *Sicohencotyle* n. gen. comprises three species. The type species, *S. antoniomaiai* n. gen. n. sp., is described here. The remaining two species have been transferred to the genus and are now designated as *S. lebedevi* comb. n. and *S. catus* comb. n. These two species had been described within *Urocleidoides* and were considered *incertae sedis* by Kritsky *et al.* (1986) [51]. Mendoza-Palmero and Scholz (2011) [69] transferred *Urocleidoides lebedevi* to *Demidospermus*, while *Urocleidoides catus* still occupied the condition proposed by Kritsky *et al.* (1986) [51]

### *Sicohencotyle antoniomaiai* n. gen. n. sp. (Figs. 16 and 17)

urn:lsid:zoobank.org:act:355968EE-4CD3-4317-8A4E-1C51E2C29173

Type host: *Phractocephalus hemioliopterus* (Bloch & Schneider, 1801) (Pimelodidae).

Type locality: Igarapé Jari, Tapajós River Basin, municipality of municipality of Santarém (−54.876133°; −2.334050°), state of Pará, Brazil.

Other localities: Tapajós River, Pimental (−56.264613°; −4.568505°), municipality of Itaituba; and Tapajós River, National Park of Amazonia, municipality of Itaituba (−56.299889°; −4.552694°), State of Pará, Brazil.

Site of infection: Gills.

Prevalence: 2/4 (50%), 4/4 (100%), 1/2 (50%), mean intensity = 20, 5 and 2, and mean abundance of infectio*n* = 10, 5 and 1, from Igarapé Jari, Tapajós River Basin, municipality of Santarém; Tapajós River, National Park of Amazonia, municipality of Itaituba; and Tapajós River, Pimental, municipality of Itaituba, State of Pará, Brazil, respectively.

Deposited material: Holotype ZUEC PLA (218). Paratypes 6 ZUEC PLA (214–221), 4 MZUSP (8085a–g), 8 CHIOC (40496a–d, 40497a–d), 4 CHIBB (877L–880L). Voucher 3 ZUEC PLA (212, 213, 216), 1 CHIOC (40498), 1 CHIBB (881L).

Etymology: the name of the species is a Latinized noun in honor of the researcher Antonio Augusto Mendes Maia, for his contribution to the study of animal parasites in Brazil.

#### Description

Description (based on 15 specimens: 6 stained on Gomori’s trichrome and mounted in Dammar gum, and 9 mounted on Gray and Wess’s medium). Body 443 (322–707; *n* = 15) long, fusiform; trunk 118 (75–163; *n* = 14) greatest width, generally at level of testis, rarely between germarium and testis. Cephalic region distinct from body, with poorly developed cephalic lobes; cephalic glands unicellular, posterolateral to pharynx, opening in five pairs of head organs. Two pairs of eyes, posterior larger than anterior; accessory chromatic granules absent. Pharynx subspherical, 28 (22–35; *n* = 12) in diameter, muscular, glandular; esophagus short, bifurcates in two intestinal ceca confluent posteriorly to gonads, lacking diverticula. Peduncle medium-sized; haptor 71 (43–137; *n* = 13) long, 144 (60–252; *n* = 12) wide, subhexagonal, conspicuous, dorsal part posteriorly displaced from ventral part. Ventral anchor 38 (21–44; *n* = 14) long, 22 (13–41; *n* = 14) base; dorsal anchor 41 (39–45; *n* = 12) long, 23 (20–26; *n* = 11) base, both with superficial root elongated, deep root low developed, short and straight shaft, and a straight and elongated point, surpassing superficial root. Ventral bar 51 (41–64; *n* = 13) in length, distance between ends 44 (30–58; *n* = 12), slightly straight, with a flattened elevation in the medio anterior region, with ends containing posteriorly a rounded prominence and anteriorly an acute prominence; dorsal bar 49 (35–64; *n* = 12) long, distance between ends 43 (28–60; *n* = 11), slightly straight, with a continuous longitudinal groove, with ends containing posteriorly a rounded prominence and anteriorly an acute prominence. Hooks unequal, not dilated, filamentous hook loop variable; pairs 1 and 2 robust, with a delicate tip, curved blade in pair 1 and straight in pair 2, straight thumb, shaft with a proximal dilation, in pair 2 after a slight constriction; pair 3 delicate, curved blade, straight thumb, shaft tapering distally and proximally, with a proximal slight constriction followed by a weakly sclerotized dilation; pair 4 robust, with a delicate tip, curved blade, erect thumb, shaft with proximal constriction followed by a dilation; pairs 5 and 6 delicate, blunt tip, straight blade, depressed thumb, with a proximal constriction followed by a weakly dilation in pair 5 and a dilation in pair 6; pair 7 delicate, with blunt tip, erect thumb and a weakly sclerotized proximal end. Hook pairs 1–3: 14 (12–17; *n* = 19) long, pairs 4 and 5: 16 (14–18; *n* = 11) long, pair 6: 17 (14–18; *n* = 6) long, pair 7: 15 (15–17; *n* = 6) long. Common genital pore opening midventral, below level of cecal bifurcation. MCO 132 (103–160; *n* = 15) long, tubular, thickened, coiled with a proximal ring and a distal twist, both counterclockwise, diameter of the first ring 17 (9–21; *n* = 15), base irregular, expanded, strongly sclerotized, containing 2 flaps. Accessory piece 35 (20–45; *n* = 15) long, sclerotized, sheath-like, evolving the distal portion of MCO, not articulated. Prostatic reservoir not observed. Gonads in tandem, intercecal, testis posterior to germarium. Testis elliptical, 53 (42–71; *n* = 8) long, 40 (25–54; *n* = 8) wide; vas deferens arising from the mid-anterior region of the testis, enveloping dorsoventrally the left branch of intestinal cecum, dilating distally and forming the seminal vesicle. Vagina sinistral, with pre-atrium, atrium, and vaginal canal not sclerotized; presence of a claw-shaped sclerite strongly sclerotized, internal to vaginal canal and atrium, containing five distal lanciform projections; vaginal canal sigmoid. Uterus is present; ootype delta-shaped with a posterior apex; seminal receptacle resembling lips with thickened envelope epithelium. Germarium oval, 57 (38–76; *n* = 9) long and 40 (28–60; *n* = 9) wide. Egg 93 (82 and 103) long and 42 (31 and 53) wide, with a robust proximal filament. Vitellaria is well developed, extending along cecum and absent in regions of reproductive system structures.

#### Remarks

The type species, *S. antoniomaiai* n. gen. n. sp., differs from all *Demidospermus*-like species by the presence of a claw-shaped vaginal sclerite within the vaginal atrium. However, this species exhibits a morphology similar to that of species of *Boegeriella* Mendoza-Palmero & Hsiao, 2020, *Boegeriella conica* (Mendoza-Palmero, Mendoza-Franco, Acosta & Scholz, 2011), a parasite of *Platynematichthys notatus* (Jardine, 1841) (Pimelodidae) and *Brachyplatystoma juruense* (Boulenger, 1898) (Pimelodidae), and *Boegeriella ophiocirrus* (Mendoza-Palmero, Mendoza-Franco, Acosta & Scholz, 2011), a parasite of *Platystomatichthys sturio* (Kner, 1858) (Pimelodidae), all from the Amazon River Basin, Peru, especially concerning the morphology of the MCO and haptoral structures [66]. However, although the MCO of these two species is robust like those of *Sicohencotyle* n. gen. species, *B. conica* and *B. ophiocirrus* have MCO with 1.5–2.5 clockwise rings before the accessory piece, while *Sicohencotyle* n. gen. species have 2–3 counterclockwise rings before the accessory piece. The accessory piece of *B. conica* and *B. ophiocirrus* is articulated with the base of the MCO by a ligament, whereas the accessory piece of *Sicohencotyle* n. gen. species is not articulated. The haptoral structures of *Boegeriella* and *Sicohencotyle* n. gen. are quite similar, including the shapes of the anchors, with a well-developed superficial root and the anterior projections of the ends of the ventral and dorsal bars. However, the vagina of *Sicohencotyle* n. gen. species exhibits grooves, which were not observed in *B. conica* and *B. ophiocirrus*.

### *Sicohencotyle catus* (Mizelle & Kritsky, 1969) comb. n. (Figs. 18 and 19)

Syn. *Urocleidoides catus*: 381–382, figs. 46–55 [72].

Type host: *Phractocephalus hemioliopterus* (Pimelodidae).

Type locality: Amazon River Basin.

Present record: *Phractocephalus hemioliopterus,* Igarapé Jari, Tapajós River Basin, municipality of Santarém (−54.876133°; −2.334050°); Tapajós River, Pimental (−56.264613°; −4.568505°), municipality of Itaituba; and Tapajós River, National Park of Amazonia, municipality of Itaituba (−56.299889°; −4.552694°), State of Pará, Brazil.

Site of infection: Gills.

Prevalence: 4/4 (100%), 4/4 (100%), 2/2 (100%), mean intensity and mean abundance of infection = 73, 20 and 37, from Igarapé Jari, Tapajós River Basin, municipality of Santarém; Tapajós River, National Park of Amazonia, municipality of Itaituba; and Tapajós River, Pimental, municipality of Itaituba, State of Pará, Brazil, respectively.

Deposited material: Voucher 13 ZUEC PLA (193–205), 13 MZUSP (8077a–d, 8078, 8079a–b, 8080a–d, 8081a–b), 12 CHIOC (40608a–b, 40609a–c, 40610a–e, 40611a–b), 13 CHIBB (861L–873L).

#### Measurements

Measurements (based on 25 specimens: 15 stained with Gomori’s Trichrome and mounted in Canada balsam, 5 mounted in Gray & Wess’s medium, 1 mounted in Gray & Wess + GAP, 2 mounted in Hoyer’s medium, and 2 mounted in Hoyer’s+GAP). Body 318 (239–431) long; trunk 85 (48–136) greatest width at mid-body. Pharynx 20 (11–26; *n* = 19) in diameter. Haptor 48 (29–70; *n* = 25) long, 66 (47–92; *n* = 25) wide. Ventral anchor 32 (27–38; *n* = 25) long, 16 (14–20; *n* = 22) base; dorsal anchor 26 (18–32; *n* = 23) long, 14 (12–18; *n* = 17) base. Ventral bar 39 (31–52; *n* = 24) long, distance between ends 36 (29–43; *n* = 11); dorsal bar 36 (31–47; *n* = 23) long. Hooks pairs 1 and 7, 17 (13–20; *n* = 29) long; pair 2, 16 (13–20; *n* = 14) long; pairs 3 and 4, 15 (12–17; *n* = 24) long; pair 5, 25 (18–29; *n* = 13) long; pair 6, 24 (15–26; *n* = 10) long. MCO 132 (102–160; *n* = 24) long; diameter of the first ring of MCO 12 (8–17; *n* = 23); accessory piece 29 (17–42; *n* = 21) long. Testis 53 (34–71; *n* = 22) long, 31 (16–59; *n* = 22) wide. Germarium 50 (33–74; *n* = 22) long, 27 (12–43; *n* = 22) wide. Egg 81 (78–87; *n* = 4) long, 59 (43–70) wide.

#### Remarks

The morphology of the *Sicohencotyle catus* comb. n. specimens were found to corroborate the observations made by Mizelle and Kritsky (1969) [72]. Its main characteristics are 4 eyes, similar anchors, a ventral bar with an irregular margin, slightly curved in the middle, and a dorsal bar, often with a median anterior notch (Figs. 18 and 19). According to the authors, the gonads of *S. catus* n. comb. can be arranged in tandem or overlapped; however, the specimens observed in the present study showed exclusively gonads arranged in tandem (Fig. 18). Mizelle and Kritsky (1969) [72] observed that the MCO of *S. catus* comb. n. has two to three rings, with a fin-shaped extension in the distal region and contains a simple accessory piece, which was also observed in the specimens of the present study: however, the precise morphology and origin of the extensions observed in the distal region of the MCO are challenging to determine, although they resemble barbs (Figs. 18 and 19). According to Mizelle and Kritsky (1969) [72], the vagina is ventral, elongated, and associated with a tube. However, we observed a vagina not sclerotized and with a sinistral opening (Fig. 18). We can confirm that *S. catus* comb. n. does not present the vaginal sclerite, a synapomorphy of *Urocleidoides* species [51, 72].

### Phylogenetic inferences

Two most parsimonious (length = 365) and best-scored trees (14.8) were obtained, which after TBR branch-swapping and summarization using strict Nelsen consensus, came out in a single consensus tree of 366 in length, 14.84 of score, consistency index (CI) 0.41, and retention index (RI) 0.57 (Fig. 20). The phylogenetic hypothesis depicted in this tree confirmed that *Demidospermus* is not monophyletic, since its monophyly is rejected in all equally parsimonious trees. On the other hand, it confirms the monophyly of *Ameloblastella*, *Aphanoblastella*, and other lineages formed by *Demidospermus*-like species, while suggesting the non-monophyly of *Nanayella*.

*Demidospermus sensu stricto* is defined by clade E2, supported by three main synapomorphies (the presence of tegument annulations, both articulated haptoral bars, and the presence of a sclerotized constriction in the middle of the ventral bar) (Fig. 20, S1). This clade stands as a sister group to clade E1, composed of *Aphanoblastella* species. The remaining *Demidospermus* species, which do not share the same synapomorphies as *Demidospermus sensu stricto* (Fig. 20, S1) and do not parasitize loricariid fishes, have been reorganized into other genera (either newly proposed or resurrected) when synapomorphies could be identified. When synapomorphies were not readily apparent, these species were considered to be *sedis mutabilis* or *incertae sedis*.

*Demidospermus centromochli* Mendoza-Franco & Scholz, 2009, a parasite of *Centromochlus heckelii* (de Filippi, 1853) (Auchenipteridae) from Iquitos, Peru, is positioned in a polytomy with clades C and D (Fig. 20). Due to this positioning, we propose that this species be classified as *sedis mutabilis*. Meanwhile, *Demidospermus annulus* Marcotegui & Martorelli, 2011, a parasite of *Parapimelodus valenciennis* (Lütken, 1874) (Pimelodidae) from Buenos Aires, Argentina, which grouped in clade F and is sister to clade G, is designated here as *incertae sedis*. In the same way, *Demidospermus brevicirrus* Mendoza-Palmero, Scholz, Mendoza-Franco & Kuchta, 2012, parasite of *Pimelodus* sp. (Pimelodidae) from Iquitos, Peru, which formed the clade H2, sister to the clade H3, is also designated as *incertae sedis*. Additionally, *Demidospermus cornicinus* Kritsky & Gutierrez, 1998, parasitizing pimelodids from Argentina and Brazil, *Demidospermus idolus* Kritsky & Gutierrez, 1998, found in *Pimelodus albicans* (Valenciennes, 1840) (Pimelodidae) from Buenos Aires, Argentina and *Demidospermus armostus* Kritsky & Gutierrez, 1998, also parasitizing pimelodids from Argentina and Brazil, which aligns closely with *D. armostus* appearing as a sister taxon to clade L, should all be considered *incertae sedis*. Similarly, *Demidospermus mortenthaleri* Mendoza-Palmero, Scholz, Mendoza-Franco & Kuchta, 2012, a parasite of *Brachyplatystoma juruense* (Boulenger, 1898) (Pimelodidae) from Peru, which grouped within clade T, which is sister to clade U is designated here as *incertae sedis*. Despite the provision of supplementary morphological data for *Urocleidoides amazonensis* Mizelle & Kritsky, 1969 (clade R), this species remains classified as *incertae sedis*.

To organize the remaining species of *Demidospermus*, grouped into clades C, H1, H3, M, Q1, and U, we propose the establishment of new genera (*i.e.*, *Rhabdolachosus* n. gen., *Martorellius* n. gen., *Magnanchistrius* n. gen., and *Sicohencotyle* n. gen.) as well as the resurrection of existing ones (*i.e.*, *Omothecium* Kritsky, Thatcher & Boeger, 1987 and *Paramphocleithrium* Suriano & Incorvaia, 1995). The rationale and detailed justification for these taxonomic changes were previously elaborated in the section Taxonomic acts.

*Ameloblastella* spp. formed the clade A, sister to the remaining dactylogyrids analyzed (clade B). It is supported by a coiled MCO, with 1–1.5 rings preceding the accessory piece which is articulated with the MCO base through a ligament. This clade appeared as a sister group to the remaining *Demidospermus*-like species. A new species (Figs. 8–10, Tables 2–4) is recognized for the genus from specimens recovered of *P. hemioliopterus* (Pimelodidae) from Tapajós River Basin, Brazil.

Clade B is supported by a long peduncle, a slightly straight ventral bar with no continuous grooves at its ends, and a dorsal bar with continuous grooves. These characters support the proposal of a new genus, *Rhabdolachosus* n. gen., to organize these species, which are proposed to be re-assigned as *Rhabdolachosus araguaiaensis* (Cepeda & Luque, 2010) comb. n., *Rhabdolachosus brachyplatystomae* (Cepeda & Luque, 2010) comb. n., and *Rhabdolachosus ceccarellii* (Cepeda & Luque, 2010) comb. n. (Figs. 11–13), all parasites of *Brachyplatystoma filamentosum* (Lichtenstein, 1819) (Pimelodidae) from the State of Mato Grosso, Brazil.

Species of *Demidospermus sensu stricto* (clade E2) (Fig. 20) formed a monophyletic clade, supported by the presence of annulations in the tegument, by the ventral and dorsal bars articulated, V, U, or W-shaped, by the presence of a sclerotized narrowing in the middle of the ventral bar. This set of character differentiates these species from the remaining *Demidospermus*-like species. As the type species of the genus, *D. anus*, a parasite of *L. anus* from Buenos Aires, Argentina, arose within this group, it is considered the natural grouping of *Demidospermus*.

Clade H (Fig. 20) is supported by a globose MCO base, with two flaps extending from it, a sinistral vaginal aperture, next to the MCO level, and by the absence of dilated hooks. *Omothecium* Kritsky, Thatcher & Boeger, 1987 is resurrected here to organize these species, and we suggest they pass to be called *Omothecium macropteri* (Mendoza-Franco & Scholz, 2009) comb. n., *Omothecium luckyi* Kritsky, Thatcher & Boeger, 1987, and *Omothecium pinirampi* Kritsky, Thatcher & Boeger, 1987.

The sister clade of *Omothecium* spp. (clade H2) is formed by *D*. *brevicirrus i.s.* and five other species previously attributed to *Demidospermus*. With the exception of *D*. *brevicirrus i.s.*, the remainder of these species form clade H3, which is supported by the sinuous, proximally dorsal, and distally ventral vaginal canal. A new genus is erected to encompass these species, which are suggested to pass to be called *Martorellius valenciennesi* (Gutiérrez & Suriano, 1992) comb. n., *Martorellius paravalenciennesi* (Gutiérrez & Suriano, 1992) comb. n., *Martorellius curvovaginatus* (Mendoza-Palmero & Scholz, 2011) comb. n. *Martorellius peruvianus* (Mendoza-Palmero & Scholz, 2011) comb. n., and *Martorellius striatus* (Mendoza-Palmero & Scholz, 2011) comb. n.

There was a clade formed by *Paramphocleithrium bidiverticulatum* Suriano & Incorvaia, 1995, a parasite of *Pimelodus albicans* (Valenciennes, 1840) (Pimelodidae) from de La Plata River, Buenos Aires, Argentina, and *Paramphocleithrium ichthyocercus* (Monteiro, Kritsky & Brasil-Sato, 2010) comb. n., a parasite of *Pimelodus maculatus* Lacepède, 1803 (Pimelodidae) from São Francisco River, near Três Marias, State of Minas Gerais, Brazil. Therefore, for this clade M, which is supported by a large and urn-shaped vaginal atrium, the resurrection of *Paramphocleithrium* Suriano & Incorvaia, 1995 is suggested.

Two clades were identified as a sister group to *Paramphocleithrium* spp. One of them consists of *Nanayella* species (clade O), characterized by the absence of a prostatic reservoir, a large vaginal atrium with fringes or spines, and a not continuous longitudinal groove at the ends of the dorsal bar. This clade includes the type species, *Nanayella aculeatrium* Acosta, Mendoza-Palmero, Silva & Scholz, 2019, but other species of *Nanayella* clustered in different clades (clade Q and S), suggesting their non-monophyly. In clade Q, *Nanayella amplofalcis* Acosta, Mendoza-Palmero, Silva & Scholz, 2019 appeared as sister to the clade Q1, formed by *Demidospermus leptosynophallus* Kritsky & Gutierrez, 1998, a parasite of *Bergiaria westermanni* (Lütken, 1874) (Pimelodidae), *Demidospermus majusculus* Kritsky & Gutierrez, 1998, a parasite of *Pimelodus albicans* (Valenciennes, 1840) (Pimelodidae), and *Demidospermus uncusvalidus* Gutierrez & Suriano, 1992, parasites of pimelodids, heptapterids, and auchenipterids from Brazil and Argentina. This clade Q1 is distinguished from the others by two flaps at the base of the male copulatory organ (MCO), an articulated ventral bar, haptoral bar V, U, or W-shaped, dilated hook pairs 1, 2, and 7 composed of two subunits, and the presence of a recurved extremity in the point of the ventral and dorsal anchors. To accommodate these species, a new genus, *Magnanchistrius*, is proposed, and the species are reclassified as *Magnanchistrius leptosynophallus* (Kritsky & Gutierrez, 1998) comb. n., *Magnanchistrius majusculus* (Kritsky & Gutierrez, 1998) comb. n., and *Magnanchistrius uncusvalidus* (Gutierrez & Suriano, 1992) comb. n.

Sister to *U. amazonensis i. s.* (clade R), arose a clade including *Nanayella megorchis* (Mizelle & Kritsky, 1969), *Demidospermus mortenthaleri* Mendoza-Palmero, Scholz, Mendoza-Franco & Kuchta, 2012, *Demidospermus lebedevi* (Kritsky & Thatcher, 1976), *Urocleidoides catus i. s.* Mizelle & Kritsky, 1969, and an undescribed *Demidospermus*-like species. The latter three species constitute a distinct clade characterized by the presence of 2–3 rings in the MCO, before the accessory piece, presence of grooves in the vaginal atrium, and a medium-sized peduncle. To accommodate these species, a new genus, *Sicohencotyle* n. gen., is proposed, resulting in the reclassification of *Sicohencotyle lebedevi* (Kritsky & Thatcher, 1976) comb. n., *Sicohencotyle catus* (Mizelle & Kritsky, 1969) comb. n., and the designation of a new species, *Sicohencotyle antoniomaiai* n. gen. n. sp.

## Discussion

The findings presented here must be contextualized within the realm of *Demidospermus*-like species, a group that exhibits clear polyphyly. While many of the morphological traits discussed are either exclusive to specific groups or widely shared across different lineages, their phylogenetic significance may vary when considered in a broader context that includes other dactylogyrid lineages. This study proposes four new genera, resurrects two others, and describes two new species. However, it is important to note that this paper does not offer a definitive phylogeny of the *Demidospermus*-like species. Instead, it aims to stimulate collaborative discussions and integrate various contributions towards a more comprehensive understanding of the phylogeny within this group of dactylogyrids.

Not all species of *Ameloblastella* and *Aphanoblastella*, or other well-defined genera like *Boegeriella*, were examined in the present study; therefore, the synapomorphies that sustain the clades we found (Fig. 20), based on morphological phylogeny, may change as more terminals or taxonomical tools are added to future inferences. However, the diagnosis provided for many of the *Demidospermus*-like species, previously in a state of taxonomic uncertainty, is as comprehensive as possible. Our goal was to provide a more accurate classification for species of uncertain placement and to provide a morphological framework that may, if deemed useful, serve as a basis for future morphological descriptions and evolutionary inferences.

While delineating individual species of Neotropical gill monopisthocotyls poses minimal challenges, defining genera remains problematic in many instances due to the scarcity of synapomorphies that unequivocally characterize each genus [2]. This becomes evident when assessing the level of homoplasy of the studied characters (S1), as nearly 50% of them exhibit RI or CI less than 0.5. This suggests that within this group of parasites, the possibility of developing new adaptive structures is quite restricted. This scenario of evolutionary convergence is also observed in other groups of monopisthocotyls, such as the gyrodactylids [18]. For some groups of dactylogyrids, such convergence is attributed to the fact that these parasites occupy similar niches [91]. The dactylogyrids analyzed here parasitize phylogenetically close hosts, all of which are siluriforms and many of them pimelodids, which can act as a convergent selective force for the evolution not only of haptoral structures, but also reproductive structures such as the MCO, though host morphological traits have also been proved to act as such an evolutionary force [93]. However, we cannot rule out the possibility that the observed homoplasies stem from shared ancestry, *i.e.*, plesiomorphy. After all, *Demidospermus*-like species appear to belong to a clade of dactylogyrids exclusively parasitizing siluriforms, corresponding to clade A of Mathews *et al.* (2021) [61] and clades B1–5 of Kmentová *et al.* (2022) [46]. Nonetheless, apart from the limited number of synapomorphies, most genera analyzed herein are supported by unique sets of characteristics.

The monophyly of *Nanayella* has been proved uncertain (Fig. 20), but it was beyond the scope of this study to propose any alternative hypothesis that could reorganize this genus recently established based on morphological and molecular evidence. When proposing the genus, Acosta *et al.* (2019) [1] described four species and reclassified *U. megorchis* as *N. megorchis*. In their study, maximum likelihood analysis did not yield significant support for the clade (bootstrap less than 60), yet Bayesian inference exhibited robust support (0.96). This same pattern was repeated in subsequent studies that revealed low support for the genus [65, 78, 106], culminating in evidence underscoring the non-monophyletic nature of the genus [61, 105]. However, more recently, the monophyly of *Nanayella* was reinstated with strong support for both Bayesian and maximum likelihood analyses [79]. Nevertheless, as additional lineages are identified, this instability will probably undergo more precise characterization.

Many phylogenetic inferences based on molecular data have revealed a close phylogenetic relationship between species of *Nanayella*, *Boegeriella*, and *D. mortenthaleri i. s*. [66, 78, 79, 89, 90, 105, 106]. In our investigation, *Nanayella* was also recovered in close relationship with *D. mortenthaleri i. s*., although with the inclusion of species of *Magnanchistrius* n. gen. within this clade, which had not been evaluated in other phylogenetic contexts until now. While it is acknowledged that omissions of taxa can have adverse effects on the outcomes of phylogenetic analyses [46], we opted not to include *Boegeriella* in our final inference (Fig. 20). This genus shares some phylogenetic characteristics with one of the genera proposed here, *Sicohencotyle* n. gen., while presenting distinguishing features such as the presence of an accessory piece articulated to the base of the MCO and haptoral hooks of equal shape. These characteristics of *Boegeriella* placed it, in our exploratory inferences, within *Ameloblastella*, suggesting the non-monophyly of this genus. Within the context of such pronounced homoplasy, the manual downweighing of these characters could indeed exert a discernible impact on the topology, potentially clustering the species of *Boegeriella*, *Nanayella*, and *D. mortenthaleri i. s*. in closer proximity. However, such an approach would inherently contradict our proposition of automatically weighing homoplasies. Hence, to avert premature taxonomic perplexity, we deemed it more prudent to omit *Boegeriella* from our inferential analyses.

The attempt to reallocate species with uncertain classification to other genera permeates the group of *Demidospermus*-like species; however, most of these attempts have aimed to bring other species into *Demidospermus* rather than the opposite. While these initiatives are important, they should be based on integrative studies, where multiple lines of evidence, at least beyond classical morphology, support a new understanding of a particular group of species. Otherwise, the authoritative nature of taxonomy is reinforced. In this context, the only proposal that reclassified one of the *Demidospermus*-like species to another genus using additional tools beyond those used in the taxon description was by Acosta *et al.* (2019) [1], who placed *U. megorchis* within *Nanayella*, since there was both morphological and phylogenetic evidence supporting this decision.

Some species synonymies, such as those recorded for *D. leptosynophallus* (syn. *Demidospermus mandi*) França, Isaac, Pavanelli & Takemoto, 2003, and *Demidospermus cornicinus* (syn. *Demidospermus labrosi* França, Isaac, Pavanelli & Takemoto, 2003) [21], also suffer from a lack of support from integrative evidence. These species were synonymized based solely on morphology, without the addition of other taxonomic tools. Regarding *D. mandi* and *D. leptosynophallus*, the species are morphologically very similar, especially concerning the morphology of the MCO and vagina. However, concerning *D. labrosi* and *D. cornicinus*, there are some differences in the morphology of the accessory piece and bars. Moreover, these species were described based on distinct hosts and localities. To determine the morphological boundaries that support the species and distinguish it from others, it is necessary to conduct an integrative study, adding other tools beyond classical morphology. Especially because there are precedents for other groups of monopisthocotyls, such as *Thaparocleidus* Jain, 1952 and *Lamellodiscus* Johnston & Tiegs, 1922, which have revealed the existence of a relative morphological conservatism [80, 92]. This phenomenon considers the presence of more taxa than previously described and recorded under the same morphotypes.

The data matrix we provided (Table S2) can be useful for species classification, as it can be utilized in any software designed for the creation, editing, and management of spreadsheets that include filtering capabilities. This effectively functions as an interactive taxonomic key. Many of the taxonomic discussions we conducted in the remarks section were based on the character matrix, which allowed us to summarize the universe of shared characters. *Demidospermus*-like species that do not fit into any of the taxa provided in the character matrix should, especially under a phylogenetic context, have new genera proposed for them.

## Conclusion

In conclusion, while this study represents a significant step forward in clarifying the taxonomic status of *Demidospermus*-like species, it also highlights the complexities and challenges that remain. The identification of homoplastic characters underscores the limitations in our current understanding and suggests that the potential for developing new adaptive structures within this group is constrained. As we continue to unravel the phylogenetic relationships and morphological distinctions within these parasites, it is crucial that future research incorporates integrative approaches, combining morphological, molecular, and ecological data, to refine the classification and evolutionary history of these species. This study, with its proposed new genera (*Rhabdolachosus* n. gen., *Martorellius* n. gen., *Magnanchistrius* n. gen. and *Sicohencotyle* n. gen.), genera resurrections (*Omothecium* Kritsky, Thatcher & Boeger, 1987 and *Paramphocleithrium* Suriano & Incorvaia, 1995), and species descriptions (*Sicohencotyle antoniomaiai* n. gen. n. sp., and *Ameloblastella sakulocirra* n. sp.), serves as a foundation for future investigations, but much work remains to achieve a comprehensive and stable phylogeny for the *Demidospermus*-like species and related dactylogyrids.

## Supplementary material

The Supplementary materials of this article are available at https://www.parasite-journal.org/10.1051/parasite/2025034/olm. In the supplementary material S1, we provide comprehensive resources to complement the main manuscript. The supplementary materials include:

*Table S1*: Detailed listing of dactylogyrids studied. *Supplementary Material S1.1*: Commented list of characters used in the study. Each character is discussed, providing context and justification for its inclusion in the phylogenetic analysis. *Supplementary Material S1.2*: Script used for phylogenetic inference. This script allows for the replication of the analysis and serves as a reference for methodological approaches in similar studies. *Table S2*: Analyzed species and their respective characters. This table is structured to allow for easy filtering and sorting, making it a functional interactive taxonomic key. *Table S3*: Character matrix in Nexus format, which can be directly utilized in phylogenetic software for further analysis or verification of our findings.
