# Supplementary material for: Morphological phylogeny on the unnatural grouping of Demidospermus-like species (Monopisthocotyla, Dactylogyridae) with the proposal of new genera, genera resurrections, and descriptions of new species
Source: Parasite. 2025 Aug 5;32:49. doi: 10.1051/parasite/2025034 (PMC12324567; doi:10.1051/parasite/2025034)
Supplement: Supplementary file 1 — Table S1: Detailed listing of dactylogyrids studied. [file parasite-32-49-s1.pdf]

Table S1. Dactylogyrids studied from different collections

**Species**	**Host**	**Locality**	**Material studied**
Ameloblastella chavarriai (Price, 1938)	*Rhamdia quelen* (Quoy & Gaimard, 1824)	Trinidad and Tobago, Trinidad, Cumuto River near Coryal	Voucher: USNM 1368745 (2 slides).
		Mexico, Yucatan, Ixin-Ha Cenote	Voucher: USNM 1384158 (5 slides).
Pseudovancleaveus (=*Ameloblastella*) *paranaensis* (França, Isaac, Pavanelli & Takemoto, 2003)	*Iheringichthys labrosus* (Lütken, 1874)	Brazil, Floodplain of the upper Paraná River	Paratype: CHIOC 34588a-d.
Ameloblastella pirarara Mathews, Domingues, Maia, Silva, Adriano & Aguiar, 2021	*Phractocephalus hemiliopterus* (Bloch & Schneider, 1801)	Brazil, Igarapé Jari, Tapajós River Basin, Santarém, Pará	Holotype: ZUECPLA 140<br>Paratype: ZUECPLA 141-144; MZUSP 7959a-b, 7960a-b.
Ameloblastella unapi Mendoza-Franco & Scholz, 2009	*Calophysus macropterus* (Lichtenstein, 1819)	Peru, Loreto, Iquitos, UNAP Exper Fish Farm	Holotype: USNM 1396529.<br>Paratype: USNM 1396530 (2 slides).
Aphanoblastella aurorae Mendoza-Palmero, Scholz, Mendoza-Franco & Kuchta, 2012	*Goeldiella eques* (Müller & Troschel, 1849)	Peru Loreto Iquitos, Santa Clara	Paratype: USNM 1400341 (1 slide), 1400342 (2 slides), 1400343 (2 slides).
Aphanoblastella travassosi (Price, 1938), morphotype A	*Rhamdia quelen* (Quoy & Gaimard, 1824)	Trinidad and Tobago, Trinidad, Cumuto River near Coryal	Voucher: USNM 1368746 (3 slides).
Aphanoblastella travassosi (Price, 1938), morphotype B		Mexico, Yucatan, Ixin-Ha Cenote	Voucher: USNM 1384159 (6 slides).
Cosmetocleithrum gussevi	*Oxydoras niger*	Brazil, Amazonas, Lago	Holotype: INPA-PA 266-

Kritsky, Thatcher & Boeger, 1986	(Valenciennes, 1821)	Janauacá	1.<br>Paratype: INPA PA 266-2, INPA 158b.
Cosmetocleithrum striatuli Abdallah, Azevedo & Luque, 2012	*Trachelyopterus striatulus* (Steindachner, 1877)	Brazil, Guandu River, Rio de Janeiro	Holotype: INPA 532.<br>Paratype: 533 a-c.
Demidospermus anus Suriano, 1983	*Loricariichthys anus* (Valenciennes, 1835)	Argentina, Buenos Aires, Chascomus	Voucher: USNM 1382363 (3 slides).
	Loricariichthys platymetopon Isbrücker & Nijssen, 1979	Brazil, Paraná, Porto Rico	Voucher: CHIBB (237L–243L).
Demidospermus armostus Kritsky & Gutierrez, 1998	*Syndontis clarias* (=*Pimelodus maculatus* Lacepède, 1803	Argentina, Buenos Aires, Rio de La Plata	Holotype: USNM 1382347.<br>Paratype: USNM 1382348 (6 slides).
Demidospermus araguaiaensis Cepeda e Luque 2010	*Brachyplatystoma filamentosum* (Lichtenstein, 1819)	Brazil, Mato Grosso, Araguaia River	Holotype: CHIOC 37326.<br>Paratype: CHIOC 37327.
Paramphocleithrium (=*Demidospermus*) *bidiverticulatum* (Suriano & Incorvaia, 1995)	*Syndontis clarias* (=*Pimelodus maculatus* Lacepède, 1803)	Argentina, Buenos Aires, Rio de La Plata	Voucher: USNM 1382350 (3 slides).
	Pimelodus albicans (Valenciennes, 1840)		Voucher: USNM 1382351 (3 slides).
Demidospermus brachyplatystomae Cepeda e Luque 2010	*Brachyplatystoma filamentosum* (Lichtenstein, 1819)	Brazil, Mato Grosso, Araguaia River	Holotype: CHIOC 37320.<br>Paratype: CHIOC 37321 a–b, 37322 a–d.
Demidospermus brevicirrus Mendoza-Palmero, Scholz, Mendoza-Franco &	*Pimelodus* sp.	Peru, Loreto, Iquitos, Santa Clara	Paratype: USNM 1400335 (1 slide), 1400336 (3 slides), 1400337 (1 slide),

Kuchta, 2012			1400338 (2 slides).
Demidospermus ceccarellii Cepeda e Luque 2010	*Brachyplatystoma filamentosum* (Lichtenstein, 1819)	Brazil, Mato Grosso, Araguaia River	Holotype: CHIOC 37323.<br>Paratype: CHIOC 37324 a–d, 37325 a–e.
Demidospermus centromochli Mendoza-Franco & Scholz Kuchta, 2009	*Centromochlus heckelii* (De Filippi, 1853)	Peru, Loreto, Iquitos	Holotype + Paratype: USNM 1396527 (1 slide).<br>Paratype: USNM 1396528 (1 slide).
Demidospermus cornicinus Kritsky & Gutierrez, 1998	*Bergiaria westermanni* (Lütken, 1874)	Argentina, Buenos Aires, Rio de La Plata	Holotype: USNM 1382359 .<br>Paratype: USNM 1382360 (4 slides).
Demidospermus curvovaginatus Mendoza-Palmero & Scholz, 2011	*Pimelodus* sp.	Peru, Loreto, Iquitos	Paratype: USNM 1399527 (1 slide).<br>Voucher: USNM 1399528 (2 slides).
		Peru, Santa Clara	Voucher: USNM 1399529 (1 slide).
Demidospermus ichthyocercus Monteiro, Kritsky & Brasil-Sato, 2010	*Pimelodus maculatus* Lacepède, 1803	Brazil, Minas Gerais, Santa maria	Holotype: INPA 516; 18.<br>Paratype: INPA 517a-i; USNM 1397347 (6 slides).
Demidospermus idolus Kritsky & Gutierrez, 1998	*Pimelodus albicans* (Valenciennes, 1840)	Argentina, Buenos Aires, Rio de La Plata	Holotype: USNM 1382355 (1 slide).<br>Paratype: USNM 1382356 (4 slides).
Demidospermus labrosi (=*Demidospermus cornicinus*) França, Isaac, Pavanelli & Takemoto, 2003	*Iheringichthys labrosus* (Lütken, 1874)	Brazil, Paraná, Rio Paraná, Baia, Lagoa dos Patos	Holotype: CHIOC 34594a.<br>Paratype: CHIOC 34587 (a-c), 34594 (b-c), 34595, 34596, 34597.

Urocleidoides (=*Demidospermus*) *lebedevi* (Kritsky & Thatcher, 1976)	*Pimelodus grosskopfi* Steindachner, 1879	Colombia, Valle, Cali, Juanchito, Rio Cauca	Holotype: USNM 1368845.<br>Paratype: USNM 1368846 (3 slides), 1368847 (3 slides); CHIOC 31222.
Demidospermus leptosynophallus Kritsky & Gutierrez, 1998	*Bergiaria westermanni* (Lütken, 1874)	Type locality: Argentina, Buenos Aires, Rio de La Plata	Holotype: USNM 1382353.<br>Paratype: USNM 1382354 (4 slides).
Demidospermus luckyi	*Pinirampus pirinampu* (Spix and Agassiz, 1829)	Brazil, Amazonas, Manaus, Janauacá Lake	Holotype: INPA 058
Demidospermus macropteri Mendoza-Franco & Scholz, 2009	*Calophysus macropterus* (Lichtenstein, 1819)	Peru, Loreto, Iquitos, UNAP Exper Fish Farm	Holotype: USNM 1396531.<br>Paratype: USNM 1396532 (2 slides).
Demidospermus majusculus Kritsky & Gutierrez, 1998	*Pimelodus albicans* (Valenciennes, 1840)	Argentina, Buenos Aires, Rio de La Plata	Holotype: USNM 1382357.<br>Paratype: USNM 1382358 (5 slides).
Demidospermus mandi Kritsky & Gutierrez, 1998) França, Isaac, Pavanelli & Takemoto, 2003	*Iheringichthys labrosus* (Lütken, 1874)	Brazil, Paraná, Rio Paraná, Baia, Lagoa dos Patos	Holotype: CHIOC 34586a.<br>Paratype: CHIOC 34586b, 34593 (a-b), 34591, 34592, 34589, 34590.
Demidospermus mortenthaleri Mendoza-Palmero, Scholz, Mendoza-Franco & Kuchta, 2012	*Brachyplatystoma juruense* (Boulenger, 1898)	Peru, Loreto, Iquitos, Santa Clara de Nanay	Paratype: USNM 1400329 (1 slide), 1400330 (1 slide), 1400331 (2 slides).
Demidospermus paranaensis Ferrari-Hoeinghaus, Bellay, Takemoto e Pavanelli,	*Loricariichthys platymetopon* Isbrücker & Nijssen, 1979	Brazil, Paraná, Upper Paraná River	Holotype: CHIOC 37255a.<br>Paratype: CHIOC

2010			37255b-e.
Demidospermus paravalenciennesi Gutiérrez & Suriano, 1992	*Syndontis clarias* (*Pimelodus maculatus* Lacepède, 1803)	Locality: Argentina, Buenos Aires, Rio de La Plata	Voucher: USNM 1382349 (3 slides).
		Brazil, Minas Gerais	Voucher: USNM 1397353 (3 slides).
Demidospermus peruvianus Mendoza-Palmero & Scholz, 2011	*Pimelodus ornatus* Kner, 1858	Peru, Loreto, Iquitos	Paratype: USNM 1399524 (2 slides)
	Pimelodus sp.		Voucher: USNM 1399525 (1 slide), 1399526 (1 slide).
Demidospermus pinirampi	*Pinirampus pirinampu* (Spix and Agassiz, 1829)	Brazil, Janauacá Lake, Manaus, Amazonas	Holotype: INPA 059
	Pimelodina flavipinnis Steindachner, 1876	Brazil, Reservoir of Lajeado, middle Tocantins River, municipality of Porto Nacional, Tocantins	Voucher: ZUEC PLA 69-72; INPA 728-736
Demidospermus prolixus Franceschini, Zago, Müller, Francisco, Takemoto & da Silva, 2017	*Loricaria prolixa* Isbrücker & Nijssen, 1978	Brazil, Sapucaí-Mirim River, São Paulo	Voucher: CHIBB 233L–236L
Demidospermus rhinelepisi Acosta, Scholz, Blasco-Costa, Alves & da Silva, 2017	*Rhinelepis aspera* Spix & Agassiz, 1829	Brazil. Aguapeí River, São Paulo	Paratypes: CHIBB 325-328L<br>Voucher: CHIBB 329-340L
Demidospermus spirophallus Franceschini, Zago, Müller, Francisco, Takemoto & da Silva, 2017	*Loricaria prolixa* Isbrücker & Nijssen, 1978	Brazil, Sapucaí-Mirim River, São Paulo	Voucher: CHIBB 226L–232L

Demidospermus striatus Mendoza-Palmero & Scholz, 2011	*Pimelodus* sp.	Peru, Loreto, Iquitos	Paratype: USNM 1399530 (2 slides).<br>Voucher: USNM 1399531 (2 slides).
Demidospermus uncusvalidus Gutierrez & Suriano, 1992	*Syndontis clarias* (*Pimelodus maculatus* Lacepède, 1803)	Argentina, Buenos Aires, Rio de La Plata	Voucher: USNM 1382352 (3 slides).
		Locality: Brazil, Minas Gerais	Voucher: USNM 1397354 (3 slides).
Demidospermus valenciennesi Gutierrez & Suriano, 1992	*Parapimelodus valenciennis* (Lütken, 1874)	Argentina, Buenos Aires, Rio de La Plata	Voucher: USNM 1382361 (10 slides).
Urocleidoides (=*Nanayella*) *megorchis* (Mizelle & Kritsky, 1969)	Type host: *Sorubim lima* (Bloch & Schneider, 1801)	Type locality: Brazil, Amazon River Basin	Holotype: USNM 1366584.
Vancleaveus cicinnus Kritsky, Thatcher and Boeger, 1986	*Phractocephalus hemioliopterus* (Bloch & Schneider, 1801)	Brazil, Tapajós River, National Park of Amazonia, Itaituba, PA	Voucher: ZUEC PLA 145–154, MZUSP 7961a–k, 7962a–b, 7963a–l
Vancleaveus janauacaensis Kritsky, Thatcher & Boeger, 1986	*Pterodoras granulosus* (Valenciennes, 1821)	Brazil, Amazonas, Janauacá Lake	Holotype: INPA PA 262-1.<br>Paratype: INPA 262-2, INPA 262-3.
		Locality Peru, Loreto, Iquitos, Itaya River	Voucher: USNM 1400353 (6 slides), 1400354 (1 slide).
