# Supplementary material for: Morphological phylogeny on the unnatural grouping of Demidospermus-like species (Monopisthocotyla, Dactylogyridae) with the proposal of new genera, genera resurrections, and descriptions of new species
Source: Parasite. 2025 Aug 5;32:49. doi: 10.1051/parasite/2025034 (PMC12324567; doi:10.1051/parasite/2025034)
Supplement: Supplementary file 2 — Supplementary Material S1.1: Commented list of characters used in the study. Each character is discussed, providing context and justification for its inclusion in the phylogenetic analysis. [file parasite-32-49-s2.pdf]

S1.1. Commented list of characters

0 – Tegument annulations (1 step, CI = 1.00, RI = 1.00): absent (0); conspicuous or inconspicuous (1). This character was reported by Ferrari-Hoeinghaus et al. (2010) [22] in describing *Demidospermus paranaensis* Ferrari-Hoeinghaus, Bellay, Takemoto & Pavanelli, 2010. Posteriorly, Franceschini et al. (2018) [25] reported the state incospicuous in *Demidospermus prolixus* Franceschini, Zago, Müller, Francisco, Takemoto & da Silva, 2017 and *Demidospermus spirophallus* Franceschini, Zago, Müller, Francisco, Takemoto & da Silva, 2017. Such character was observed as conspicuous in the holotype (CHIOC 37255a) and paratypes (CHIOC 37255b, 37255e) of *D. paranaensis* and in the voucher (USNM 1382363, 3 slides) of *Demidospermus anus* Suriano, 1983.

1 – Eyes (12 steps, CI = 0.42, RI = 0.67): absent (0); 1 pair, the posterior (1); 2 pairs, posterior and/or anterior fused (2); 2 pairs, posterior closer than anterior (3); 2 pairs, posterior larger than anterior and equidistant (4); 2 pairs, posterior larger and closer than anterior (5).

2 – Accessory granules in cephalic and anterior trunk regions (10 steps, CI = 0.10, RI = 0.50): present (0); absent (1).

3 – Head organs (16 steps, CI = 0.38, RI = 0.44): 2 pairs (0); 3 pairs (1); 4 pairs (2); 5 pairs (3); 7 pairs (4); 9 pairs (5); 6 pairs (6).

4 – Male copulatory organ (MCO) <shape> (3 steps, CI = 0.33, RI = 0.86): coiled (0); not coiled (1);

5 – Type of coiled MCO: a coil with counterclockwise rings (0) (2 steps, CI = 0.50, RI = 0.67); a corkscrew-like coil (1). A coiled MCO is here considered those which has one or more clearly defined rings. However, a very tightly rings is also considered coiled, as those corkscrew-like coiled MCO observed in the morphotype A of *Aphanoblastella travassosi* (Price, 1968) (USNM 1368746), *Aphanoblastella juizforense* Carvalho, Tavares and Luque 2009 [7], *Ameloblastella martinae* Mendoza-Palmero, Rossin, Irigoitia & Scholz, 2020 [36] and *Ameloblastella sakulocirra* sp. n. (ZUEC PLA XXXX, CHIBB-UNESP YYYY).

6 – Type of non–coiled MCO (5 steps, CI = 0.40, RI = 0.40): a curved tube, forming incomplete rings, C, G, J or U-shaped (0); a sigmoid or sinuous tube (1); a rough cylindrical tube (2). The state 0 is widely present in *Demidospermus*-like species which have more robust MCO, apparently not so distensible, whose morphology clearly describes the shape of C, J or U letters, sometimes assumed to form incomplete rings. The state 1 is present in some species of *Demidospermus s.s.* ([25], also in *D. anus* USNM 1382363 and *D. paranaensis* CHIOC 37255a, CHIOC 37255b-e), *Demidospermus centromochli* Mendoza-Franco & Scholz, 2009 *s.m.* (USNM 1396528), also in some species of *Nanayella* Acosta, Mendoza-Palmero, Silva & Scholz, 2019 [2]. State 2 was only observed in *Demidospermus brevicirrus* Mendoza-Palmero, Scholz, Mendoza-Franco & Kuchta, 2012 [37], also in THE specimens USNM 1400335, 1400336, 1400337 and 1400338.

7 – Smaller number of rings in the MCO preceding the accessory piece (8 steps, CI = 0.63, RI = 0.57): 1–1.5 (0); 2 (1); 3 (2); 11 (3); 13 (4); 4–4.5 (5).

8 – Larger number of rings in the MCO preceding the accessory piece (12 steps, CI = 0.55, RI = 0.38): 1–1.5 (0); 2–2.5 (1); 3 (2); 4–4.5 (3); 11 (4); 14 (5); 5 (6).

9 – MCO <distal part> (6 steps, CI = 0.33, RI = 0.00): contiguous or distally acute (0); expanded (1); containing extensions resembling barbs (2). The state 0 is the more generalized one, refers to MCO that ends

with no significant change in its thickness or to those with tapering ends. The state 1 is just present in *Demidospermus cornicinus* Kritsky & Gutierrez, 1998 and in *Sicohencotyle antomaia* gen. n. sp. n. The state 2 is observed in species like *Demidospermus araguaiaensis* Cepeda & Luque, 2010 (CHIOC 37326 and 37327), *Magnanchistrius majusculus* comb. n. (Kritsky & Gutierrez, 1998), *Sicohencotyle catus* (Mizelle & Kritsky, 1969) comb. n. Mizelle & Kritsky, 1969 (ZUEC PLA XXXX, CHIBB YYYY), *Urocleidoides amazonensis* Mizelle & Kritsky, 1969 (ZUEC PLA XXXX, CHIBB YYYY).

10 – Sac of the MCO (2 steps, CI = 0.50, RI = 0.00): absent (0); present (1). This character is only observed in specimens of *Ameloblastella antomaia* sp. n. (ZUEC PLA XXXX, CHIBB YYYY) and *Demidospermus ceccarellii* Cepeda & Luque, 2010 (CHIOC 37323, 37324 a–d and 37325 a–e).

11 – MCO base <shape> (12 steps, CI = 0.33, RI = 0.53): bifurcate (0); globose (1); doubly globose (2); expanded (3); rounded, simple, just differentiated from the MCO (4). The base of the MCO is defined here as rounded, simple or only differentiated from the MCO, when its width is not greater than three times the thickness of the proximal part of the MCO. Above that, it is considered expanded or globular, depending on the shape. If the shape is undefined, it is expanded, if it is elliptical, it is globular. When there are two of these globular structures, it is called doubly globular. The base of the MCO can be considered bifurcated when it has two distinct and non-globular parts, each of them greater than three times the thickness of the proximal part of the MCO.

12 – MCO base <expansions> (12 steps, CI = 0.25, RI = 0.63): absent (0); sclerotized fringe-like or irregular ornamentation around (1); 1 flap (2); 2 flaps (3). This character, when present, is seen as a structure attached to the base of the MCO, with clearly defined boundaries that evidence its distinct nature from the base. These extensions surround the base, so they are not extensions of the base, although they may be associated with them.

13 – Accessory piece <type> (11 steps, CI = 0.45, RI = 0.33): sheath-like, with lateral lobe (0); two sclerotized pieces, proximally or distally, articulated (1); rod-shaped (2); one sclerotized piece, sheath-like (3); sheath-like, spiraled (4); rounded, small (5).

14 – Accessory piece <articulation> (7 steps, CI = 0.29, RI = 0.62): directly articulated with the proximal region of the MCO or with the MCO base (0); articulated by means of a ligament (1); not articulated (2).

15 – Prostatic reservoir (8 steps, CI = 0.63, RI = 0.40): absent (0); 1, sac-shaped (1); 1, biloculated (2); 2 (3); 2 prostatic sac (4); large, strong, c-shaped (5).

16 – Gonads (1 step, CI = 1.00, RI = 1.00): tandem (0); overlapped (1).

17 – Vaginal aperture (8 steps, CI = 0.38, RI = 0.50): sinistral, next to the MCO level (0); sinistral, next to germarium level (1); sinistral, between the MCO and the germarium level (2); ventral, at level of the germarium (3).

18 – Vagina (9 steps, CI = 0.18, RI = 0.61): an atrium and a pre-atrium (0); an atrium (1); a pre-atrium (2). The shape of the vagina is not always well represented in the species descriptions. Sometimes, this representation lacks the relationship that the vagina maintains with the body wall, where it opens, and this makes it difficult to define its shape through original descriptions. A complete vagina is here considered as that has an atrium and a pre-atrium. An atrium corresponds to an expansion of the vaginal canal, which forms a space for the reception of sperm. Sometimes the atrium becomes funneled leading to a channel that opens to the

outside, this channel is defined as the pre-atrium. When the formation of the atrium is not observed and the vaginal canal opens directly to the outside, then it is considered that the vagina has only the pre-atrium.

19 – Vaginal atrium (5 steps, CI = 0.33, RI = 0.50): sclerotized (0); muscular (1); absent (2).

20 – Vaginal pre-atrium (12 steps, CI = 0.08, RI = 0.54): sclerotized (0); muscular (1); absent (2).

21 – Vaginal atrium <shape> (3 steps, CI = 0.67, RI = 0.67): cup shaped or tulip-like (0); infundibuliform or funnel shaped (1); urn-shaped (2). An infundibuliform or funnel-shaped atrium has a narrower bottom compared to the distal part of the atrium. Cup-shaped or tulip-shaped atriums do not show this remarkable difference between the extremities of the atrium, with the length of the bottom being approximately the same of the distal extremity of the atrium. However, both are observed in species with small vaginal atriums, unlike the urn-shaped atriums, which are always present in species that have large atriums.

22 – Vaginal atrium <length > (4 steps, CI = 033, RI = 0.60): not extend to the midline of the body (0); extends to or exceeds the midline of the body (1), as in *Paramphocleithrium ichthyocercus* comb. n. (Monteiro, Kritsky & Brasil-Sato, 2010) (INPA 516 and 517a-i).

23 – Vaginal atrium <grooves> (6 steps, CI = 0.14, RI = 0.63): absent (0); present (1).

24 – Vaginal atrium <fringes> (8 steps, CI = 0.13, RI = 0.13): absent (0); present (1). These structures are flexible extensions from the atrium wall that border the distal end of the atrium.

25 – Vaginal pre-atrium <grooves> (3 steps, CI = 0.33, RI = 0.00): absent (0); present (1).

26 – Structure within the vaginal atrium (3 steps, CI = 0.67, RI = 0.00): absent (0); a wrinkled ring at its bottom (1); claw-shaped internal structure (2). These structures are rigid or have little flexibility, seen as additional structures, attached to the vaginal atrium.

27 – Vaginal canal <sclerotization> (8 steps, CI = 0.25, RI = 0.50): sclerotized (0); not sclerotized (1); partially or weakly sclerotized or delicate (2). The sclerotization of structures can be easily perceived through staining techniques, such as Gomori's trichrome. Through this technique, sclerotized structures are stained in intense red, while non-sclerotized structures are stained in blue. Partially or weakly sclerotized structures acquire faint pinkish colors. Other preparations, such as Hoyer’s or Gray and Wess' medium or glycerin and picric acid (GAP), also contribute to this differentiation, since although they reveal the same shade for sclerotized structures, they present different intensities according to the level of sclerotization.

28 – Vaginal canal <shape> (13 steps, CI = 0.36, RI = 0.61): a curved or sinuous tube (0); a straight or just curved in extremities tube (1); a sigmoid, s-shaped tube (2); doubly sigmoid, proximally and distally s-shaped tube (3); a tube with loops (4); sinuous, proximally dorsal and distally ventral (5).

29 – Vaginal canal <distal part> (4 steps, CI = 0.50, RI = 0.33): contiguous (0); bifurcated (1); pileus-like or expanded (2). Character state 2 was observed in only two species of *Ameloblastella*, *Ameloblastella pirarara* Mathews, Domingues, Maia, Silva, Adriano & Aguiar, 2021 (ZUECPLA 140-144 MZUSP 7959a-b, MZUSP 7960a-b) and in *Ameloblastella unapi* Mendoza-Franco & Scholz, 2009 (USNM 1396529, 1396530), however species of other genera not examined on this study can present such state of character.

30 – Seminal receptacle (7 steps, CI = 0.29, RI = 0.29): absent (0); present with pouch-like shape, semi-spherical or globose (1); present with a sclerotized mass inside (2). Character state 2 was observed in only two species of *Ameloblastella*, *Ameloblastella pirarara* Mathews, Domingues, Maia, Silva, Adriano & Aguiar, 2021 (ZUECPLA 140-144 MZUSP 7959a-b, MZUSP 7960a-b) and in *Ameloblastella unapi* Mendoza-Franco & Scholz, 2009 (USNM 1396529, 1396530).

31 – Peduncle <length> (14 steps, CI = 0.23, RI = 0.55): short (from 1 to 7% compared do total length) (0); medium (from 8 to 15% compared do total length) (1); long (larger than 16% compared do total length) (2); absent (3). The peduncle size was measured from the narrowing following the trunk, close to the vitellaria, until the beginning of the haptor. Classes of length were defined according to the Sturges` rule.
32 – Haptoral patches (6 steps, CI = 0.17, RI = 0.62): present (0); absent (1).
33 – Haptoral bars in adult <number> (2 steps, CI = 0.50, RI = 0.63): 1 ventral, 1 dorsal, both not articulated (0); 1 ventral, 1 dorsal, both articulated (1); 1 ventral and 1 dorsal, only the ventral articulated (2); 1 ventral and 1 dorsal, only the dorsal articulated (3). Ventral and dorsal bars can articulate through their medial ends, without an element that fuses one to the other or can articulate by means of a sclerotized and narrowed piece in the middle.
34 – Ventral bar <shape> (4 steps, CI = 0.33, RI = 0.64): bowed or some variation between V, U and W (0), not articulated; slightly straight (1). In order to determine the extent of bar straightness, the distance between the ends was divided by the total length of the bar. Bars with values exceeding 0.9 were classified as slightly straight.
35 – Ventral bar <sclerotized narrowing in the middle> (2 steps, CI = 1.00, RI = 1.00): absent (0); present (1).
36 – Ventral bar <distal extremities> (15 steps, CI = 0.13, RI = 0.28): with tapering ends (0); with expanded ends (1); with contiguous end (2).
37 – Medio-anterior or medio-posterior projection in the ventral bar (2 steps, CI = 0.50, RI = 0.90): absent (0); present (1). This projection is a distinct piece, rather than a mere projection of the bar itself, as evidenced by its base, which sets its dimensions relative to the bar.
38 – Ventral bar <longitudinal groove> (10 steps, CI = 0.30, RI = 0.46): absent (0); present, continuously along the bar (1); present, not continuously at the ends of the bar (2); present, not continuously along the bar (3). These grooves are regular, easily detectable, longitudinal in relation to the bar and not to the worm body. Irregular grooves seen in different ways in different specimens were not considered.
39 – Ventral bar <transversal groove> (6 steps, CI = 0.17, RI = 0.00): absent (0); present (1).
40 – Dorsal bar <shape> (4 steps, CI = 0.40, RI = 0.50): bowed or some variation between V, U and W (0); slightly straight (1); two articulated bars (2).
41 – Dorsal bar <sclerotized narrowing in the middle> (3 steps, CI = 0.25, RI = 0.40): absent (0); present (1).
42 – Dorsal bar <distal extremities> (13 steps, CI = 0.15, RI = 0.35): with contiguous end (0); with expanded ends (1); with tapering ends (2).
43 – Medio-anterior or medio-posterior projection in the dorsal bar (4 steps, CI = 0.25, RI = 0.40): absent (0); present (1).
44 – Dorsal bar <longitudinal groove> (10 steps, CI = 0.33, RI = 0.50): absent (0); present, continuously along the bar (1); present, at the ends of the bar (2); present, in the two most proximal parts, starting from the middle (3).
45 – Haptoral hooks (3 steps, CI = 0.33, RI = 0.89): all approximately equal in shape (0); some unequal in shape (1).

46 – Hook pairs dilated composed by one unity (5 steps, CI = 0.40, RI = 0.67): absent (0); pairs 1 and 7 (1); pair 1 (2).

47 – Hook pairs dilated composed by two subunits (1 steps, CI = 1.00, RI = 1.00): absent (0); pairs 1, 2 e 7 (1).

48 – Hook 5 longer and/or with shank thinner than the others (4 steps, CI = 0.25, RI = 0.81): absent (0); present (1).

49 – Hook 5 with depressed, flattened or vestigial thumb (3 steps, CI = 0.33, RI = 0.88): absent (0); present (1).

50 – Hook 5 with underdeveloped or undeveloped point (3 steps, CI = 0.33, RI = 0.88): absent (0); present (1).

51 – Hook 6 longer and /or with shank thinner than the others (2 steps, CI = 0.50, RI = 0.93): absent (0); present (1).

52 – Hook 6 with depressed, flattened or vestigial thumb (2 steps, CI = 0.50, RI = 0.94): absent (0); present (1).

53 – Hook 6 with underdeveloped or undeveloped point (2 steps, CI = 0.50, RI = 0.93): absent (0); present (1).

54 – Hook 2 with shank significantly reduced (2 steps, CI = 0.80, RI = 0.64): absent (0); present (1).

55 – Hook 5 smaller than the others (2 steps, CI = 0.50, RI = 0.50): absent (0); present (1).

56 – Hook 2 with shank composed by two subunits, the proximal sometimes detached (3 steps, CI = 0.33, RI = 0.50): absent (0); present (1).

57 – Fold in the superficial root of dorsal anchor (1 steps, CI = 1.00, RI = 1.00): absent (0); present (1).

58 – Ventral anchor <tip of point> (2 steps, CI = 0.50, RI = 0.67): recurved (0); not recurved (1).

59 – Dorsal anchor <tip of point> (2 steps, CI = 0.50, RI = 0.67): recurved (0); not recurved (1).
