# Supplementary material for: Morphological phylogeny on the unnatural grouping of Demidospermus-like species (Monopisthocotyla, Dactylogyridae) with the proposal of new genera, genera resurrections, and descriptions of new species
Source: Parasite. 2025 Aug 5;32:49. doi: 10.1051/parasite/2025034 (PMC12324567; doi:10.1051/parasite/2025034)
Supplement: Supplementary file 3 — Supplementary Material S1.2: Script used for phylogenetic inference. This script allows for the replication of the analysis and serves as a reference for methodological approaches in similar studies. [file parasite-32-49-s3.pdf]

S1.2 Script for run TNT analysis

```
mxram 1000;
nstates NUM;
nstates NOGAPS;
piwe= ;
proc "datamatrix.nex";

xpiwe]=10;

xpiwe&homoplasy;

xpiwe (*0.25 <5/12;

hold 100000;
outgroup "outgroup";
sect: slack 15 ; xmult=hits 10 noupdate nocss replic 10 ratchet 10 fuse 1 drift 5 hold 100 noautoconst keepall ;
bbreak = tbr ;
nelsen * ;
tchoose { strict } ;
ttags = ;
rseed[;
hold 1000; sub 1;
bbreak: fillonly tbr;
hold 10000; sub 2;
bbreak: fillonly tbr;
hold 20000; sub 20;
bbreak: fillonly tbr;
bbreak = tbr ;
bsupport [;
ttags ) ;
ttags ;
export *+ "tree.tre" ;
```
